# TFEB‐dependent lysosome biogenesis is required for senescence

**DOI:** 10.15252/embj.2022111241

**Published:** 2023-03-27

**Authors:** Rachel Curnock, Katy Yalci, Johan Palmfeldt, Marja Jäättelä, Bin Liu, Bernadette Carroll

**Affiliations:** ^1^ School of Biochemistry University of Bristol Bristol UK; ^2^ Research Unit for Molecular Medicine, Department of Clinical Medicine Aarhus University Aarhus Denmark; ^3^ Cell Death and Metabolism Unit, Center for Autophagy, Recycling and Disease Danish Cancer Society Research Center Copenhagen Denmark; ^4^ Department of Cellular and Molecular Medicine, Faculty of Health Sciences University of Copenhagen Copenhagen Denmark

**Keywords:** autophagy, lysosome, senescence, TFEB, Autophagy & Cell Death

## Abstract

The accumulation of senescent cells is recognised as a driver of tissue and organismal ageing. One of the gold‐standard hallmarks of a senescent cell is an increase in lysosomal content, as measured by senescence‐associated β‐galactosidase (Senβ‐Gal) activity. The lysosome plays a central role in integrating mitogenic and stress cues to control cell metabolism, which is known to be dysregulated in senescence. Despite this, little is known about the cause and consequence of lysosomal biogenesis in senescence. We find here that lysosomes in senescent cells are dysfunctional; they have higher pH, increased evidence of membrane damage and reduced proteolytic capacity. The significant increase in lysosomal content is however sufficient to maintain degradative capacity of the cell to a level comparable to proliferating control cells. We demonstrate that increased nuclear TFEB/TFE3 supports lysosome biogenesis, is a hallmark of multiple forms of senescence and is required for senescent cell survival. TFEB/TFE3 are hypo‐phosphorylated and show constitutive nuclear localisation in senescence. Evidence suggests that several pathways may contribute to TFEB/TFE3 dysregulation in senescence.

## Graphical abstract

The transcription factor TFEB induces lysosome proliferation in senescent cells, counterbalancing reduced lysosomal efficiency and maintaining autophagic capacity.

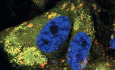

## Introduction

Senescence is defined as an irreversible or stable exit from the cell cycle and occurs in response to potentially transformative intrinsic or extrinsic stress such as oncogene activation, oxidative stress and DNA damage (Lopez‐Otin *et al*, 2013; Hernandez‐Segura *et al*, 2018). As such, senescence is an important tumour suppressor mechanism, and more recently, senescence has been shown to have physiological roles in wound healing and during tissue repair (Storer *et al*, 2013; Paramos‐de‐Carvalho *et al*, 2021). However, senescent cells have been observed to accumulate in many tissues, including lungs (Birch *et al*, 2015) muscle (Sousa‐Victor *et al*, 2014), skin (Victorelli *et al*, 2019), bone (Farr *et al*, 2017) and liver (Ogrodnik *et al*, 2017) with age. Genetic ablation of p16/INK4a‐positive senescent cells (Baker *et al*, 2011, 2016; Farr *et al*, 2017), as well as several pharmacological “senolytic,” or senescence‐killing interventions (Zhu *et al*, 2015; Chang *et al*, 2016) have been demonstrated to improve health span in several mouse models, reducing inflammation and improving tissue function, integrity and regeneration capacity. Trials are now underway to test whether senolytics, primarily targeting anti‐apoptotic pathways, improve symptoms of age‐related decline and disease in humans (Kirkland & Tchkonia, 2020).

Senescence is a dynamic and heterogenous program downstream of different stressors. All programs however ultimately lead to a characteristic and common set of hallmarks. These include activation of DNA damage response pathway, chromatin remodelling, activation of cyclin‐dependent kinase (CDK) inhibitors, p16/INK4a and p21, organelle biogenesis, a secretory phenotype (referred to as Senescence‐Associated Secretory Phenotype, SASP) and dysregulation of mTORC1 signalling (Campisi, 2013; Tchkonia *et al*, 2013; Carroll & Korolchuk, 2018; Hernandez‐Segura *et al*, 2018). The strength and robustness of these hallmarks differ across models (Hernandez‐Segura *et al*, 2018).

Despite not being able to divide, metabolic and growth pathways are highly active in senescence. The mammalian target of rapamycin complex 1 (mTORC1) drives many senescence phenotypes including mitochondrial biogenesis (Correia‐Melo *et al*, 2016) and translation of SASP factors (Herranz *et al*, 2015; Laberge *et al*, 2015), and its inhibition by rapamycin can slow senescence onset (Carroll & Korolchuk, 2018). We and others have demonstrated that mTORC1 is essentially constitutively active in senescence, refractory to starvation of growth factors, hormones and amino acids (Carroll *et al*, 2017). Our work indicates that changes in plasma membrane potential (Carroll *et al*, 2017) and cell‐ECM contacts (Rabanal‐Ruiz *et al*, 2021) may contribute to rewiring of growth factor signalling to mTORC1, while the Narita lab first described an elegant model whereby increased activity through the autophagy‐lysosome pathway generates increased amino acids to maintain mTORC1 activity (via a Tor‐Autophagy Spatial Coupling Mechanism, TASCC) (Narita *et al*, 2011). Indeed, one of the most defining hallmarks of senescence is the accumulation of lysosomes which is classically measured by staining for senescence‐associated β‐galactosidase activity (SA‐βGal) (Hernandez‐Segura *et al*, 2018). The lysosome is now appreciated to be a central hub for the integration of anabolic and catabolic processes in the cell (Ballabio & Bonifacino, 2020), providing a platform for the activation of mTORC1 (which is recruited via the Rag GTPases), as well as being the ultimate destination for autophagic cargo to be degraded (Laplante & Sabatini, 2012; Liu & Sabatini, 2020). Despite this important role for the lysosome in controlling the mTORC1‐autophagy pathway, and the gross changes in content and morphology of the lysosomal compartments in senescence, the cause and consequences of these changes for senescence acquisition and survival remain almost entirely unexplored.

In this study, we demonstrate that lysosomal compartments are increased in multiple forms of senescence, including oncogene‐induced senescence, replicative senescence and irradiation‐induced senescence. We reveal that lysosomes show increased signs of membrane damage and reduced proteolytic capacity but the increased biogenesis is able to compensate and ensure degradative capacity remains intact, supporting autophagy. We identify that the stress‐responsive transcription factors, TFEB/TFE3, are constitutively nuclear in all forms of senescence tested, and they are required for cell survival upon senescence induction. Mechanistically, we reveal that TFEB/TFE3 are hypo‐phosphorylated at S142 and S211, and provide evidence that multiple pathways, including p16/INK4a‐CDK4 and RagC‐mTORC1, may be dysregulated in senescence and contribute to the activation of TFEB/TFE3. Our findings support the idea that nuclear TFEB/TFE3 is a novel hallmark of senescence and is responsible for increased lysosome biogenesis, the gold‐standard marker for senescence *in vitro* and *in vivo*. These data build upon the model that complete metabolic rewiring is required to drive senescence phenotypes, including SASP. The fact that lysosomes render senescent cells more susceptible to cell death in response to lysosomotropic drugs suggests targeting lysosomes has senolytic potential.

## Results

**Figure 1:**
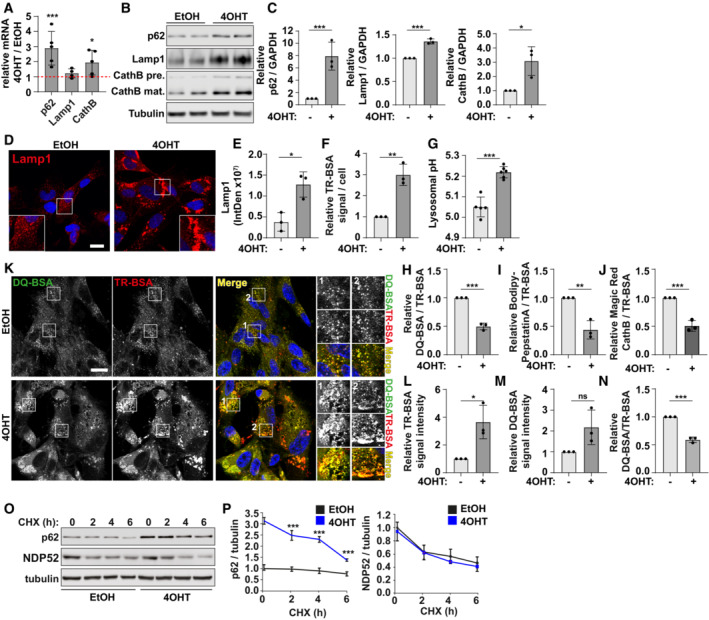
Senescence is associated with an increase in dysfunctional lysosomes AIMR90 ER:Ras^V12^ primary fibroblasts were cultured in the presence of EtOH (proliferating) or 100 nM 4OHT (for 6–8 days to induce Ras^V12^ expression and oncogene‐induced senescence, OIS). mRNA levels of 4OHT cells, normalised to *PUM1* and presented relative to EtOH control (*n* = 4 independent experimental repeats).B, CCells as in (A) were analysed by Western blot for Lamp1, p62 and Cathepsin B and quantified, normalised to GAPDH and expressed relative to EtOH (*n* = 3 independent experimental repeats).D, ECells as in (A) were fixed and immunostained for Lamp1, and signal intensity was quantified. Scale bars: 10 μm (*n* = 3 independent experimental repeats (at least 40 cells analysed per condition, from at least four fields of view, per repeat)).FCells as in (A) analysed by flow cytometry following incubation with Texas Red‐BSA (TR‐BSA) for 2 h. Graphs represent MFI, normalised to EtOH control (*n* = 3 independent experimental repeats).GCells as in (A) analysed by plate reader to measure lysosomal pH following incubation with LysoSensor Yellow/Blue (*n* = 5 independent experimental repeats).HCells as in (A) were incubated with DQ‐BSA and TR‐BSA for 2 h and analysed by flow cytometry. Graph represents ratio of DQ‐BSA/TR‐BSA MFI relative to EtOH Control (*n* = 3 independent experimental repeats).ICells as in (A) were incubated with BODIPY‐Pepstatin A (30 min) and TR‐BSA (2 h) and analysed by flow cytometry. Graph represents ratio of BODIPY‐Pepstatin A/TR‐BSA MFI relative to EtOH Control (*n* = 3 independent experimental repeats).JCells as in (A) were incubated with MagicRed Cathepsin B (2 h) and TR‐BSA (2 h). Cells were analysed by flow cytometry. Graph represents ratio of MagicRed Cathepsin B/TR‐BSA MFI relative to EtOH Control (*n* = 3 independent experimental repeats).K–NCells as in (A) were incubated with DQ‐BSA and TR‐BSA for 2 h, fixed and imaged by confocal microscopy. Relative TR‐BSA signal intensity was quantified. Relative DQ‐BSA signal intensity was quantified, and the ratio of DQ‐BSA/TR‐BSA was quantified. Scale bar: 20 μm (*n* = 3 independent experimental repeats (at least 240 cells analysed from eight fields of view per condition)).O, PCells as in (A) were incubated with 50 μg/ml cycloheximide (CHX) for the timepoints indicated. Degradation of autophagy receptors, p62 and NDP52 was assessed by Western blot and quantified (*n* = 3 independent experimental repeats). Data information: All graphs show individual data points, mean and error bars represent standard deviation. All data are analysed by 2‐tailed, nonpaired Student's *t*‐test (*< 0.05, **< 0.01, ***< 0.001). Source data are available online for this figure.

**Figure EV1:**
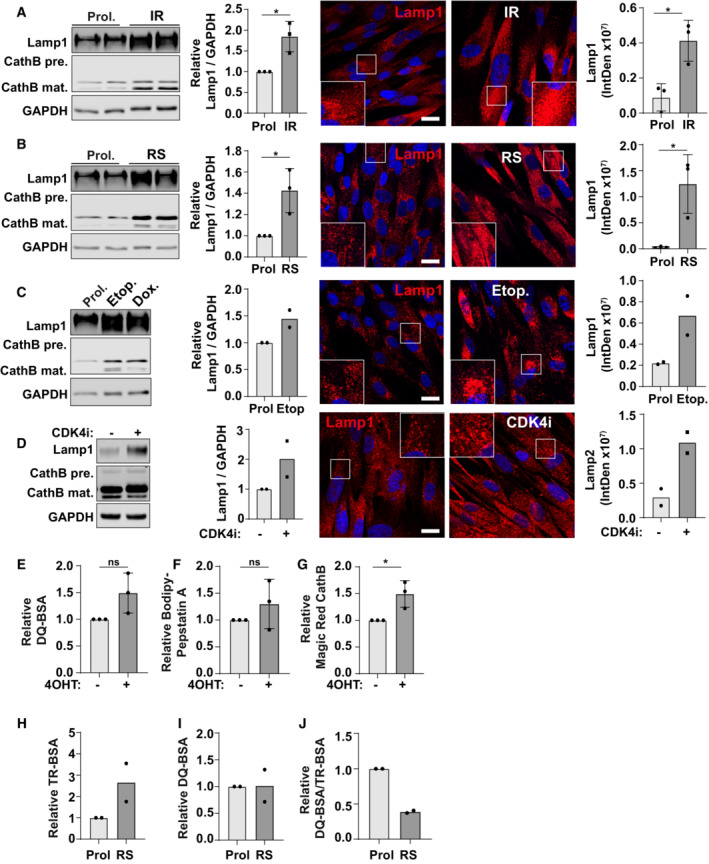
Multiple models of senescence are associated with an increase in dysfunctional lysosomes Senescence was induced in MRC5 primary fibroblasts by ionising γ‐radiation (IR; 20Gy) and analysed 10 days later by Western blot and immunofluorescence. Blots and images were quantified and represented relative to proliferating (Prol) controls. Scale bar: 20 μm (*n* = 3 independent experimental repeats (at least 40 cells analysed from at least four fields of view per repeat)).Assays as described in (A) were carried out on Replicative Senescent (RS) MRC5 cells. Scale bar: 20 μm (*n* = 3 independent experimental repeats (at least 40 cells analysed from at least four fields of view per repeat)).Assays as described in (A) were carried out on MRC5 fibroblasts treated with etoposide or doxorubicin for 7 days. Scale bar: 20 μm (*n* = 2 independent experimental repeats (at least 40 cells analysed from at least four fields of view per repeat)).Assays as described in (A) were carried out on MRC5 fibroblasts treated with CDK4 inhibitor (CDK4i) every 24 h for 7 days. Scale bar: 20 μm (*n* = 2 independent experimental repeats (at least 40 cells analysed from at least four fields of view per repeat)).Cells were incubated with DQ‐BSA (2 h), and signal was analysed by flow cytometry. Data are MFI represented relative to EtOH Control (*n* = 3 independent experimental repeats).Cells were incubated with BODIPY‐Pepstatin A for 30 min, and signal was analysed by flow cytometry. Data are MFI represented relative to EtOH Control (*n* = 3 independent experimental repeats).Cells were incubated with Magic Red Cathepsin B (2 h), and signal was analysed by flow cytometry. Data are MFI represented relative to EtOH Control (*n* = 3 independent experimental repeats).Proliferating and replicative senescent cells were incubated with TR‐BSA and analysed by flow cytometry. Data are MFI represented relative to EtOH Control (*n* = 2 independent experimental repeats).Proliferating and replicative senescent cells were incubated with DQ‐BSA and analysed by flow cytometry. Data are MFI represented relative to EtOH Control (*n* = 2 independent experimental repeats).Data from (H and I) represented as a ratio of DQ‐BSA/TR‐BSA, relative to EtOH Control (*n* = 2 independent experimental repeats). Data information: All graphs show individual data points, mean and error bars represent standard deviation. All data (where *n* = 3) are analysed by 2‐tailed, nonpaired Student's *t*‐test (*< 0.05). Source data are available online for this figure.

To investigate the biology of lysosomes in senescence, we initially confirmed previous reports that in an oncogene‐induced model of senescence (OIS, induced via 4OHT‐inducible expression of HRas^V12^), lysosomes accumulate as a result of increased transcription and protein expression (Fig 1A–F) (Narita *et al*, 2011). We confirmed that lysosomes accumulate in a number of other models of senescence, including ionising radiation (IR), replicative exhaustion (Replicative Senescence; RS), DNA damaging agents (etoposide, doxorubicin) and CDK4 inhibition (Fig EV1A–D; Narita *et al*, 2011; Carroll *et al*, 2017). LS‐MS and proteomic analysis of isolated lysosomes from proliferating and senescent cells confirmed that the preparations were enriched in lysosomal proteins, validating the methodology (Appendix Fig S1A and B, Dataset EV1). Compared to proliferating controls, the relative abundance of many lysosomal proteins was reduced in senescent lysosomal preps, including Lamp2 and Cathepsins C and D (Appendix Fig S1C and D, Dataset EV1). Those proteins upregulated in senescent lysosomal preps are enriched in membrane regulators, but are not common contaminants, suggesting there is a dysregulation of membrane trafficking pathways in senescent cells and/or an accumulation of undegraded material (Appendix Fig S1E, Dataset EV1). Together, these data may suggest that despite an increase in lysosomal content, the quality and therefore function of lysosomes is decreased in senescence.

**Figure EV2:**
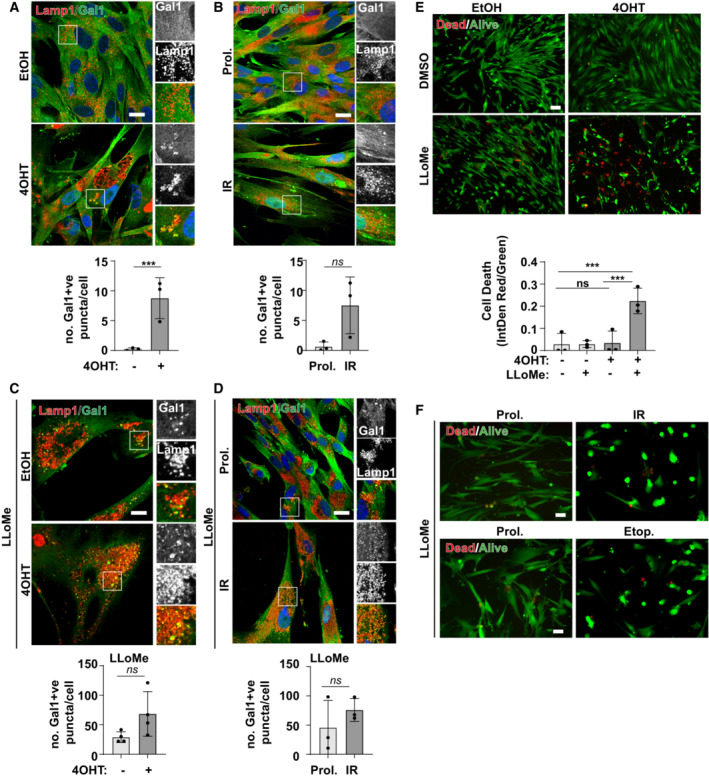
Targeting lysosomes in senescence promotes cell death EtOH and 4OHT‐treated fibroblasts were fixed, immunostained for Galectin 1 (Gal1) and Lamp1. The number of Gal1^+^ puncta was quantified. Scale bar: 10 μm (*n* = 3 independent experimental repeats (at least 40 cells analysed from at least four fields of view per repeat)).Proliferating and IR fibroblasts were fixed, immunostained for Galectin 1 (Gal1) and Lamp1. The number of Gal1^+^ puncta was quantified. Scale bar: 20 μm (*n* = 3 independent experimental repeats (at least 40 cells analysed from at least four fields of view per repeat)).EtOH and 4OHT‐treated fibroblasts were incubated in the presence/absence of LLoMe for 2 h, immunostained with antibodies against Gal1 and Lamp1, and the number of Gal1^+^ puncta was quantified. Scale bar: 20 μm (*n* = 4 independent experimental repeats (at least 40 cells analysed from at least four fields of view per repeat)).Proliferating and IR fibroblasts were incubated in the presence/absence of LloMe for 2 h, immunostained with antibodies against Gal1 and Lamp1, and the number of Gal1^+^ puncta was quantified. Scale bar: 20 μm (*n* = 3 independent experimental repeats (at least 40 cells analysed from at least four fields of view per repeat)).Cells as in (C) were incubated with cell‐permeable live/dead dye, imaged and cell death quantified. Scale bar: 100 μm (*n* = 3 independent experimental repeats (at least 300 cells analysed from at least six fields of view per repeat)).Senescent fibroblasts were incubated with LloMe and incubated with cell‐permeable live/dead dyes. Scale bar: 100 μm (*n* = 2 independent experimental repeats (at least 200 cells analysed from at least four fields of view per repeat)). Data information: All graphs show individual data points, mean and error bars represent standard deviation. All data (where *n* = 3) are analysed by 2‐tailed, nonpaired Student's *t*‐test (***< 0.001) except (E) which was analysed by one‐way ANOVA with Tukey's multiple comparison test (***< 0.001).

Consistent with this idea, lysosomes in senescence have increased luminal pH compared to proliferating controls (Fig 1G). We went on to investigate the proteolytic activity of lysosomes using a number of assays analysed by confocal microscopy and flow cytometry. Flow cytometry analysis of DQ‐BSA (assay for general proteolytic activity of lysosomes), Pepstatin A (assay for Cathepsin D expression) and Magic Red Cathepsin B (assay for Cathepsin B activity) revealed that only Magic Red Cathepsin B showed a significant increase in senescent cells (Fig EV1E–G), despite the significant (~3‐fold) increase in total lysosomal content (measured by Texas‐Red BSA, Fig 1F). When presented relative to lysosomal content, all three readouts indicate that there is a significant decrease in proteolytic capacity of lysosomes in senescent cells compared to controls (Fig 1H–J). These results were confirmed using confocal microscopy (Fig 1K–N) and further validated in replicative senescent cells which also show that the relative activity of lysosomes (by DQ‐BSA/Texas‐Red BSA) is reduced compared to proliferating controls (Fig EV1H–J). To be clear, DQ BSA/TR BSA indicate the fraction of degraded/endocytosed BSA, which decreases in senescence. While this could of course be a consequence of changes in endocytosis, our additional readouts suggest this is direct effect of changes in lysosomal activity. Consistent with the evidence that lysosomes are dysfunctional in senescent cells, there is an increase in Galectin1‐positive puncta, a marker for lysosomal membrane permeabilisation or damage in both OIS and IR models (Fig EV2A and B). Challenging senescent cells with the lysosomal permeabilisation agent, LLoMe increased Galectin1‐positive puncta and caused cell death, a phenotype consistent among multiple models of senescence (Fig EV2C–F).

Together, these data indicate that although there are more lysosomes in senescent cells, they are less active, show increased signs of membrane damage and are thus more susceptible to death in response to lysosomal damaging agents. These data also raise the possibility that lysosomes may represent a previously unrecognised target for senolytic development. Furthermore, these data indicate the large increase in expression of lysosomes can compensate for dysfunction of individual lysosomes in senescence. This is consistent with previous findings by us and others that autophagy flux, that is the turnover of autophagosomes by fusion with and degradation by lysosomes is intact (Narita *et al*, 2011; Carroll *et al*, 2017), and we show here that protein degradation of autophagy receptors occurs at similar or increased rate in senescence (Fig 1O and P).

Senescence can be induced by a range of different stressors, many of which are well known to activate the autophagy‐lysosome pathway, such as nutrient starvation and DNA damage. One of the key stress‐induced activators of the pathway is the TFEB/TFE3 family of transcription factors (Puertollano *et al*, 2018). The genes upregulated in senescence, such as p62, Cathepsin B and Lamp1 (Fig 1A; Young *et al*, 2009), are well‐known TFEB/TFE3‐responsive genes with TFEB/TFE3‐binding CLEAR (Co‐ordinated Lysosomal Expression and Regulation) motifs (Palmieri *et al*, 2011; Settembre *et al*, 2011). We hypothesised therefore that TFEB/TFE3 are active in senescence and are responsible for increased lysosome biogenesis.

**Figure 2:**
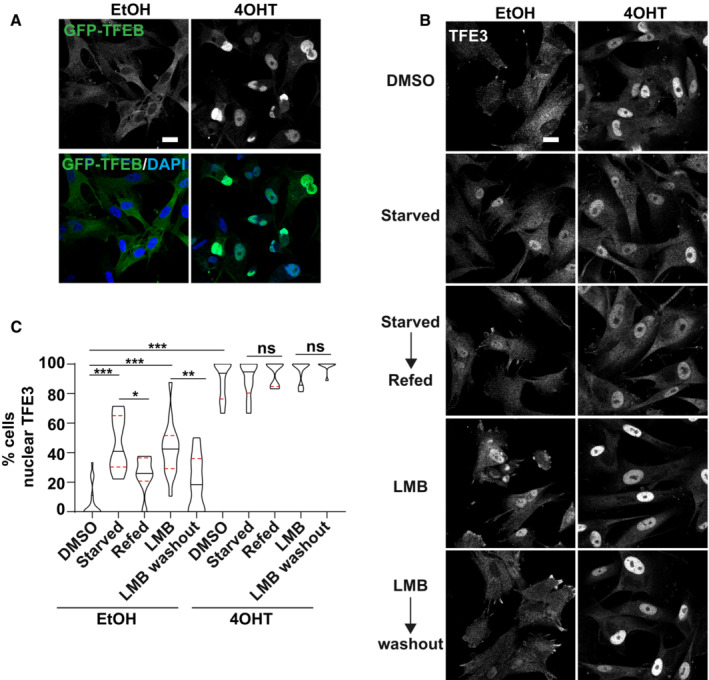
TFEB/TFE3 are activated in senescence ARepresentative images of GFP‐TFEB localisation in EtOH and 4OHT‐treated fibroblasts, in full nutrient conditions. Scale bar: 20 μm.B, CEtOH and 4OHT‐treated fibroblasts immunostained for endogenous TFE3 in the conditions indicated; starvation indicates serum starvation overnight and 1‐h amino acid starvation, refeeding was with full nutrient medium (including FCS) for 2 h. Cells were incubated with leptomycin B (LMB) for 3 h, and where indicated, wash out involved replacement of the media for a further 2 h. % cells with nuclear TFE3 was quantified. Scale bar: 20 μm (*n* = 3 independent experimental repeats (at least 150 cells analysed from at least five fields of view per repeat)). Data information: Violin blots include all datapoints from *n* = 3, lines represent median and upper and lower quartiles; Analysis: one‐way ANOVA with Tukey's multiple comparison test *P < 0.05; **P < 0.01; ***P < 0.001.

**Figure EV3:**
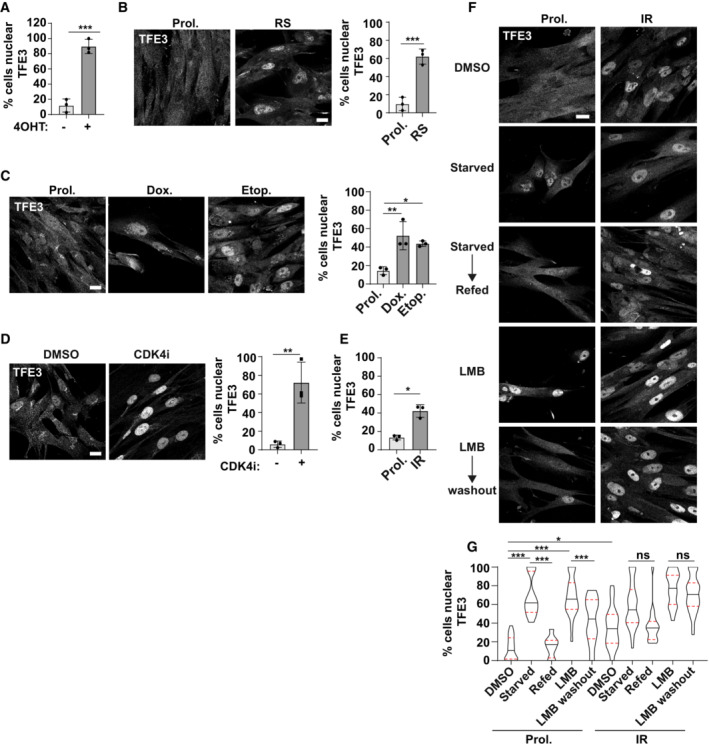
TFEB/TFE3 activation is a new hallmark of senescence Quantification of nuclear TFE3 in EtOH and 4OHT‐treated fibroblasts (*n* = 3 independent experimental repeats).Representative images and quantification of nuclear TFE3 in proliferating and replicative senescent (RS) fibroblasts. Scale bar: 20 μm (*n* = 3 independent experimental repeats (at least 100 cells analysed from at least five fields of view per repeat)).Representative images and quantification of nuclear TFE3 in proliferating versus etoposide or doxorubicin‐induced senescent fibroblasts. Scale bar: 20 μm (*n* = 3 independent experimental repeats (at least 100 cells analysed from at least five fields of view per repeat)).Representative images and quantification of nuclear TFE3 in proliferating versus CDK4i‐induced senescent fibroblasts. Scale bar: 20 μm (*n* = 3 independent experimental repeats (at least 100 cells analysed from at least five fields of view per repeat)).Quantification of nuclear TFE3 in proliferating and IR‐induced senescent fibroblasts (*n* = 3 independent experimental repeats (at least 100 cells analysed from at least five fields of view per repeat)).Proliferating and IR‐induced senescent fibroblasts immunostained for endogenous TFE3 in the conditions indicated; starvation indicates serum starvation overnight and 1‐h amino acid starvation; refeeding was with full nutrient medium (including FCS) for 2 h. Cells were incubated with leptomycin B (LMB) for 3 h and where indicated, wash out involved replacement of the media for a further 2 h. Scale bar: 20 μm.Quantification of (F); % cells with nuclear TFE3 was quantified (*n* = 3 independent experimental repeats (at least 150 cells analysed from at least five fields of view per repeat)). Data information: Violin blots in (G) include all datapoints from *n* = 3, lines represent median and upper and lower quartiles; Analysis: one‐way ANOVA with Tukey's multiple comparison test *P < 0.05; ***P < 0.001. All other panels; graphs show individual data points, mean and error bars represent standard deviation. Analysed by 2‐tailed, unpaired *t*‐test *P < 0.05; **P < 0.01; ***P < 0.001.

TFEB/TFE3 activity is regulated by shuttling between the cytoplasm and nucleus in response to stress and nutrient conditions. Here we show for the first time that oncogene‐induced senescence is associated with a significant increase in the nuclear localisation of GFP‐TFEB (Fig 2A) and endogenous TFE3 (Fig EV3A and B). Importantly, a significant increase in cells with nuclear TFE3 was observed for all senescence models tested, including replicative senescence, and in response to DNA‐damaging agents, CDK4 inhibitors and ionising radiation (Fig EV3C–E). Importantly, we found that the subcellular localisation of TFE3 in response to nutrient status is perturbed in senescence, both OIS and irradiated models (Figs 2B and C, and EV3F and G). Specifically, in proliferating cells, starvation resulted in increased nuclear localisation of TFE3 which was released back to the cytoplasm upon refeeding (Figs 2B and C, and EV3F and G). In senescence however, TFE3 remains nuclear and shows no significant change upon nutrient starvation/refeeding (Figs 2B and C, and EV3F and G). Further to this, using an assay to monitor the normal, dynamic flux of TFE3 under basal conditions, we observed that blocking the CRM1‐dependent nuclear export of TFE3 (by treatment with leptomycin B (LMB); Li *et al*, 2018; Napolitano *et al*, 2018) leads to its accumulation over a 2‐h time period in proliferating cells (Figs 2B and C, and EV3F and G). Following the wash‐out of LMB, TFE3 was released from the nucleus (Figs 2B and C, and EV3F and G). However, there was no significant change in the subcellular localisation of TFE3 in senescent cells under the same conditions (Figs 2B and C, and Fig EV3F and G). There is however an increase in nuclear TFE3 intensity in senescence in the presence of LMB, which is maintained after LMB washout. These data may indicate that TFE3 is entering the nucleus, but the export is inhibited or significantly reduced (Figs 2B and C, and EV3F and G). These data indicate there was little to no flux of TFE3 in steady‐state conditions or in response to starvation. Instead, it remained almost constitutively nuclear.

**Figure 3:**
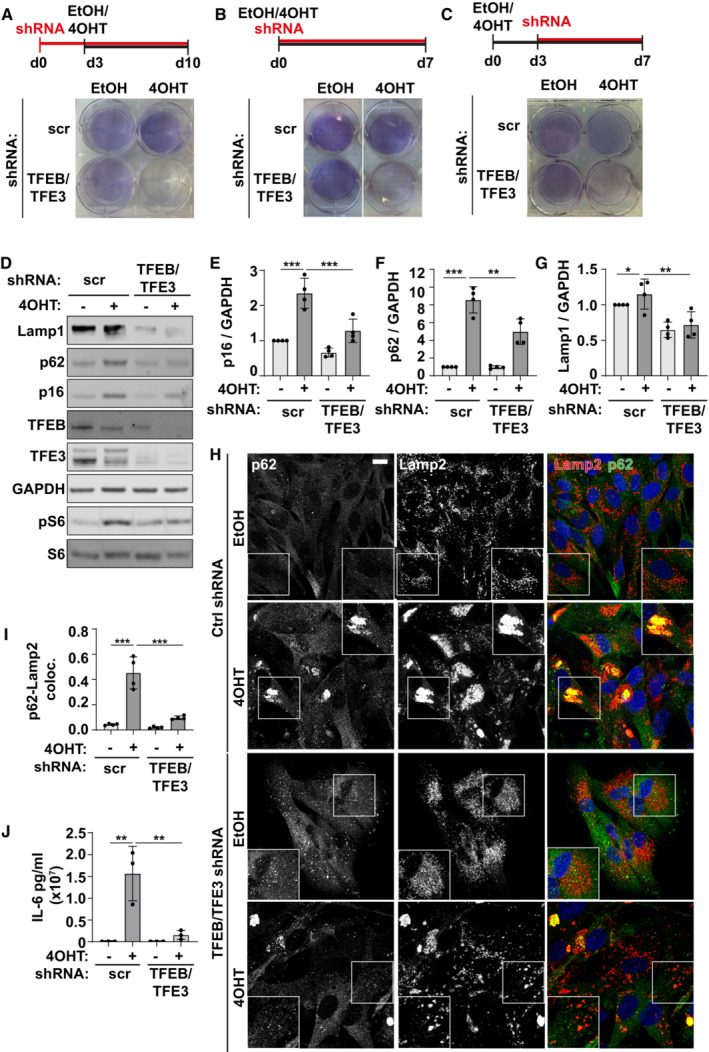
TFEB/TFE3 supports senescence‐associated phenotypes and senescent cell survival AFibroblasts were transduced with TFEB and TFE3 shRNA for 3 days followed by induction of senescence by addition of 4OHT or EtOH (control). Cells were fixed and stained with crystal violet (*n* = 2 independent experimental repeats).BFibroblasts were transduced with TFEB and TFE3 shRNA simultaneously with induction of senescence by 4OHT. Cells were fixed and stained with crystal violet (*n* = 2 independent experimental repeats).CFibroblasts were transduced with TFEB and TFE3 shRNA 3 days after induction of senescence by 4OHT. Cells were fixed and stained with crystal violet (*n* = 4 independent experimental repeats).DCells treated as in (C) were analysed by Western blotting.E–GQuantification of blots shown in (D) (*n* = 4 independent experimental repeats).HRepresentative images of cells treated as in (C, D) before being fixed and immunostained with antibodies against Lamp2 and p62. Scale bar: 20 μm.IQuantification of colocalisation (Mander's coefficient) between p62 and Lamp2 from (H) (*n* = 4 independent experimental repeats (at least 40 cells analysed from at least four fields of view per repeat)).JQuantification of ELISA for IL‐6 from cells treated as in (C–I) (*n* = 3 independent experimental repeats). Data information: All graphs show individual data points, mean and error bars represent standard deviation. All data were analysed by one‐way ANOVA with Tukey's multiple comparison test (*< 0.05, **< 0.01, ***< 0.001). Source data are available online for this figure.

**Figure EV4:**
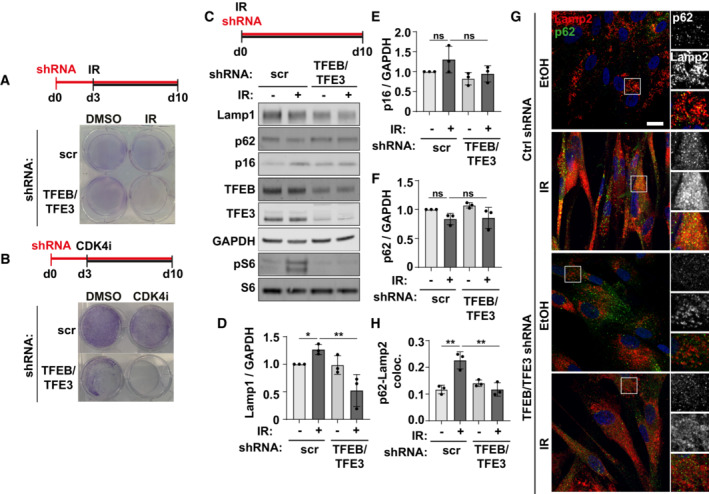
TFEB/TFE3 drives lysosomal biogenesis in multiple models of senescence AFibroblasts were transduced with TFEB and TFE3 shRNA before induction of senescence by IR. Cells were fixed and stained with crystal violet (*n* = 1).BFibroblasts were transduced with TFEB and TFE3 shRNA before induction of senescence by CDK4i. Cells were fixed and stained with crystal violet (*n* = 1).CFibroblasts were transduced with TFEB and TFE3 shRNA immediately following induction of senescence by IR (or incubated with proliferating controls) and analysed by Western blot 10 days later.D–FQuantification of blots shown in (C) as indicated (*n* = 3 independent experimental repeats).GCells treated as in (C) were fixed and immunostained for Lamp2 and p62. Scale bar: 20 μm.HQuantification of p62‐Lamp2 colocalisation (Mander's coefficient) from (G) (*n* = 3 independent experimental repeats (at least 40 cells analysed from at least four fields of view per repeat)). Data information: All graphs show individual data points, mean and error bars represent standard deviation. All data were analysed by one‐way ANOVA with Tukey's multiple comparison test (*< 0.05, **< 0.01). Source data are available online for this figure.

Evidence therefore indicates that the TFEB/TFE3 family of transcription factors contributes to increased expression of the autophagy‐lysosome pathway in multiple models of senescence. To test this, we transduced cells with shRNA targeting both TFE3 and TFEB. Interestingly, the simultaneous depletion of both TFEB and TFE3 either prior to, or simultaneously with, induction of oncogene‐induced senescence led to extensive cell death (Fig 3A and B), while control cells showed reduced growth and evidence of increased stress (e.g. loss of HMGB1 protein, increased DNA damage foci and reduced nuclear CDK4 signal) (Appendix Fig S2A–G). Similarly, the simultaneous TFEB/TFE3 knock‐down caused cell death when senescence was induced by irradiation and a CDK4 inhibitor (Fig EV4A and B). These data strongly suggest the increased expression of the autophagy‐lysosome pathway is required for senescent cell survival. Gene knock‐down 2/3 days after senescence induction allowed sufficient survival of cells to permit further analysis (Fig 3C). We observed that loss of TFE3/TFEB significantly reduced senescence markers p16, p62 and LAMP1 by Western blot (Fig 3D–G). There was no reduction however in DNA damage foci or any evidence of a reversal in cell cycle arrest (Appendix Fig S2B and C). The striking, tight spatial coupling of Lamp2 and p62 (TASCC; Narita *et al*, 2011) was significantly reduced, although not completely resolved by TFEB/TFE3 knock‐down (Fig 3H and I). At the same time, reduced expression and activity of the autophagy‐lysosome pathway led to a reduction in mTORC1 activity, measured by phosphorylation of S6 (Fig 3D) and reduced IL‐6 secretion (Fig 3J). These data are consistent with the previously described model whereby increased activity of the autophagy‐lysosome pathway in senescence contributes to the liberation of nutrients and supports mTORC1 activity and synthesis of SASP factors (Narita *et al*, 2011; Herranz *et al*, 2015; Laberge *et al*, 2015; Carroll *et al*, 2017). These results were confirmed using a second independent shRNA against TFEB and TFE3 in OIS (Appendix Fig S3A–F) and validated in irradiation (Fig EV4C–G) and CDK4 inhibitor‐induced models of senescence (Appendix Fig S3G–L). Interestingly, irradiated cells tolerated TFEB/TFE3 knock‐down better than other models (i.e. knock‐down was carried out simultaneously with irradiation without significant cell death) (Fig EV4C). This is consistent with irradiation showing weaker senescence phenotypes of interest such as Lamp1, p16 and p62 expression (Fig EV4C–F) and a lower percentage of cells with nuclear TFE3 compared to other models (Fig EV3E). Consistent with the other models tested however, irradiation was associated with increased p62‐Lamp2 colocalisation that was resolved upon loss of TFEB/TFE3 (Fig EV4G and H) supporting the idea that these transcription factors drive increased activity through the autophagy‐lysosome pathway. Furthermore, in all models tested, mTORC1 activity, which has been shown previously to be supported via autophagy‐lysosome degradation, is reduced in cells treated with TFEB/TFE3 shRNA (Figs 3D and EV4C, Appendix Fig S3A and G). Altogether, these data strongly suggest that TFEB/TFE3‐dependent increase in expression of the autophagy‐lysosome pathway is a hallmark of senescence and contributes to senescence acquisition and survival.

**Figure 4:**
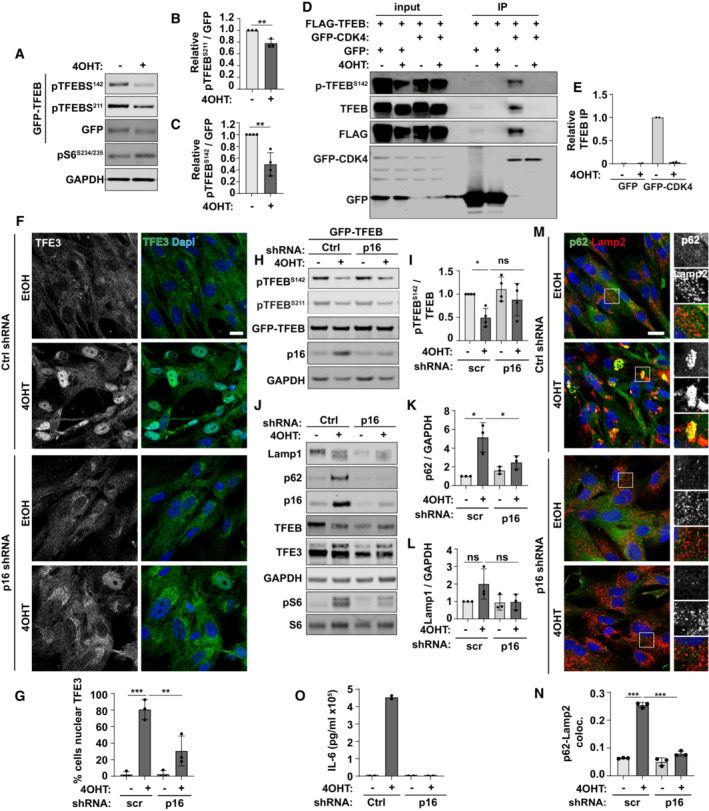
Senescence‐associated p16^INK4a^ contributes to nuclear accumulation of TFEB/TFE3 AFibroblasts stably expressing GFP‐TFEB were treated 4OHT to induce senescence (or EtOH control). Cells were lysed and subject to Western blotting for antibodies as indicated.BQuantification of (A) (*n* = 4 independent experimental repeats).CQuantification of (A) (*n* = 3 independent experimental repeats).DWestern blot of GFP immunoprecipitation of control (EtOH) and senescent (4OHT) cells expressing GFP or GFP‐CDK4 and FLAG‐TFEB.EQuantification of (D) (*n* = 2 independent experimental repeats).FFibroblasts were transduced with p16 shRNA simultaneously with induction of senescence by 4OHT. Cells were fixed and immunostained for endogenous TFE3.GQuantification of (F) (*n* = 3 independent experimental repeats (at least 150 cells analysed from at least five fields of view per repeat)).HFibroblasts stably expressing GFP‐TFEB were transduced with p16 shRNA simultaneously with induction of senescence by 4OHT. Cells were lysed and subject to Western blotting.IQuantification of (H) (*n* = 4 independent experimental repeats).JFibroblasts were transduced with p16 shRNA simultaneously with induction of senescence by 4OHT. Cells were lysed and subject to Western blotting.K, LQuantification of (J) (*n* = 3 independent experimental repeats).MFibroblasts were transduced with p16 shRNA simultaneously with induction of senescence by 4OHT. Cells were fixed and immunostained with antibodies against p62 and Lamp2, Scale bar: 20 μm.NQuantification of (M). *n* = 3 independent experimental repeats (at least 40 cells analysed from at least four fields of view per repeat).OFibroblasts were transduced with p16 shRNA simultaneously with induction of senescence by 4OHT. Cells were incubated with serum‐free media overnight which was collected and subject to ELISA analysis for IL‐6 (*n* = 3 independent experimental repeats). Data information: All graphs show individual data points, mean and error bars represent standard deviation. (B and C): Analysed by 2‐tailed, unpaired *t*‐test *P < 0.05; **P < 0.01; ***P < 0.001. All other panels analysed by one‐way ANOVA with Tukey's multiple comparison test (*< 0.05, **< 0.01, ***< 0.001). Source data are available online for this figure.

The subcellular localisation and activity of TFEB/TFE3 is controlled via phosphorylation by a number of kinases, most notably mTORC1 but also ERK, CDK4, GSK3β, Akt and PKCβ, and dephosphorylation via the phosphatase calcineurin (Puertollano *et al*, 2018). We observed a significant decreased in phosphorylation of TFEB at two residues, serine 211 and serine 142 (Fig 4A–C), which is consistent with increased nuclear accumulation of TFEB/TFE3 (Fig 2A–C). Neither calcium chelation nor calcineurin inhibition was able to significantly rescue the senescence‐associated nuclear accumulation of TFEB/TFE3 suggesting increased phosphatase activity is not responsible for this phenotype (Appendix Fig S4A). Thus, we turned our focus to kinases known to regulate TFEB/TFE3. We were particularly interested in mTORC1 and CDK4 in the context of senescence. Cyclin‐dependent kinases (CDK) 4/6 phosphorylation of TFEB/TFE3 at serine 142 promotes its nuclear export and regulates cell cycle‐dependent changes in lysosomal content (Yin *et al*, 2020). The CDK4/6 inhibitor, p16/INK4a is a key regulator of the cell cycle arrest that is a defining feature of the senescence programme (Baker *et al*, 2011; Carroll & Korolchuk, 2018). Given this and the observed failure of TFEB/TFE3 to leave the nucleus following LMB treatment and washout led us to hypothesise that increased p16/INK4a expression could inhibit the CDK4/6 phosphorylation of TFEB in senescence and prevent its export from the nucleus. Consistent with this hypothesis, senescence was associated with reduced interaction of GFP‐CDK4 and FLAG‐TFEB (Fig 4D and E). We reasoned therefore that knock‐down of p16/INK4a may release CDK4 inhibition, promoting export of TFEB/TFE3 from the nucleus of senescent cells and rescue the autophagy‐lysosome phenotypes. Since the expression of p16/INK4a is important for the induction of the whole senescence programme, rather than treating cells with shRNA prior to the induction of senescence, we instead treated shRNA and 4OHT simultaneously (Fig 4F–O, Appendix Fig S5A–D), as well as p16 shRNA several days after induction (Appendix Fig S5E–H). Knock‐down of p16/INK4a by two independent shRNAs led to a significant reduction in nuclear TFE3 (Fig 4F and G and Appendix Fig S5A and B) but interestingly did not significantly restore phosphorylation of TFEB (Fig 4H and I). p16/INK4a shRNA was however accompanied by a reduction in lysosomal phenotypes in senescence, including a reduction in p62 and Lamp2 protein levels (Fig 4J–L), reduced p62‐Lamp2 co‐localisation (Fig 4M and N and Appendix Fig S5C and D) and a significant reduction in secretion of the SASP factor, IL‐6 (Fig 4O). Knock‐down of p16/INK4a after induction caused a significant albeit smaller reduction in nuclear TFE3 (Appendix Fig S5E and F). Quantifying these latter data by measuring the intensity of nuclear versus cytoplasmic TFE3 showed more clearly that knock‐down of p16/INK4a reduces the proportion of TFEB/TFE3 from the nucleus (Appendix Fig S5G). Importantly, the levels of IL‐6 were reduced upon p16/INK4a knock‐down (Appendix Fig S5H). We confirmed knockdown of p16/INK4a restored CDK4 kinase activity, as measured by increased phosphorylation of another target, Rb (Appendix Fig S5I and J). Knockdown of p16/INK4a also reduced nuclear TFE3 (Appendix Fig S4B and C) and reduced p62‐Lamp2 co‐localisation in irradiated cells (Appendix Fig S4D and E) although the effects on total protein levels show only a partial rescue in this model (Appendix Fig S4F–H).

**Figure EV5:**
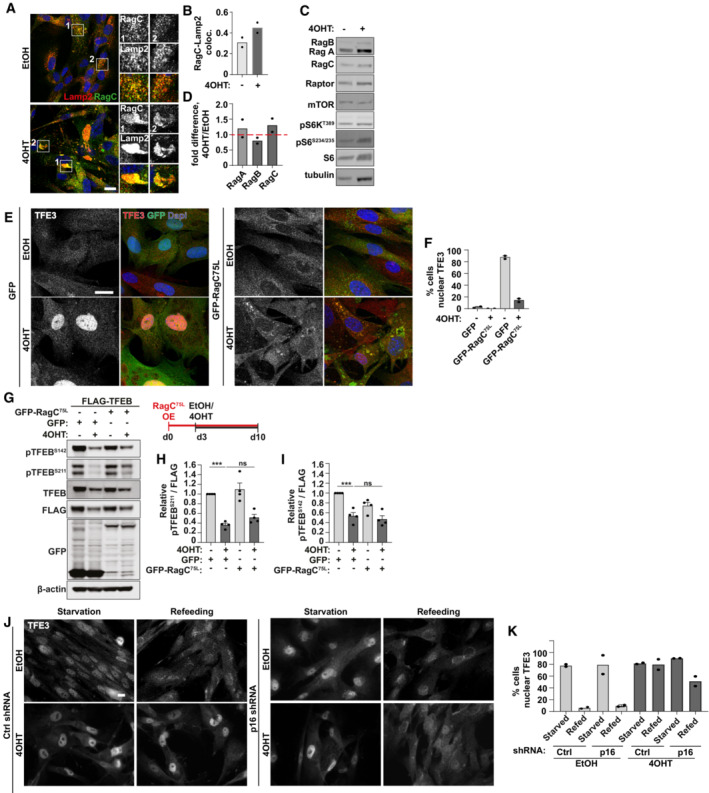
Defects in RagC‐mTORC1 axis contribute to nuclear accumulation of TFEB/TFE3 Fibroblasts treated with EtOH or 4OHT were fixed and immunostained for Lamp2 and RagC. Scale bar: 20 μm.Quantification of (B) (Mander's coefficient between Lamp2 and RagC) (*n* = 2 independent repeats (at least 40 cells analysed from at least four fields of view per repeat)).Cells as in (A) were lysed and subject to Western blot.Quantification of (C); expression of RagA, RagB and RagC, normalised to tubulin and relative to EtOH control (*n* = 2 independent experimental repeats).Fibroblasts stably expressing GFP or GFP‐RagC^75L^ were treated with EtOH or 4OHT as indicated, fixed and immunostained for endogenous TFE3. Scale bar: 20 μm.Quantification of (E) (*n* = 2 independent experimental repeats (at least 50 cells analysed in each condition from at least five fields of view per repeat)).Fibroblasts stably expressing GFP or GFP‐RagC^75L^ and FLAG‐TFEB were treated with EtOH or 4OHT as indicated. Cells were lysed and subject to Western blotting.Quantification of (G) (*n* = 4 independent experimental repeats).Quantification of (G) (*n* = 4 independent experimental repeats).Fibroblasts were transduced with p16 shRNA simultaneously with induction of senescence by 4OHT. Cells were subject to starvation (serum‐free media overnight, 1 h amino acid‐free media) or starved and refed (starvation protocol as above, refeeding with full nutrient media for 2 h). Cells were fixed and immunostained for endogenous TFE3. Scale bar: 100 μm.Quantification of (J) (*n* = 2 independent repeats (at least 100 cells analysed in each condition from at least five fields of view per repeat)). Data information: All graphs show individual data points, and error bars represent standard deviation. Data analysed by one‐way ANOVA with Tukey's multiple comparison test (***< 0.001). Source data are available online for this figure.

Although senescent cells treated with p16/INK4a shRNA showed rescue of TFEB/TFE3 nuclear localisation and a functional resolution of lysosomal‐related phenotypes in senescence, there was no significant rescue of phosphorylation at S142 or S211. These data suggest that multiple pathways contribute to TFEB/TFE3 dysregulation and therefore we turned our attention to mTORC1. It has been comprehensively demonstrated both here and previously that mTORC1 is active towards canonical substrates, such as S6K/S6 and 4EBP1 in senescence (Fig 4A) (Narita *et al*, 2011; Carroll *et al*, 2017), mTOR has been shown to localise to lysosomes in senescence (Narita *et al*, 2011; Carroll *et al*, 2017) and mTORC1‐dependent protein translation drives SASP (Herranz *et al*, 2015; Laberge *et al*, 2015). Indeed, inhibition of mTORC1 by rapamycin has been widely shown to reduce the burden of senescence and senescence‐associated phenotypes *in vitro* and *in vivo*. More recently however, several reports have demonstrated that mTORC1‐dependent phosphorylation and cytoplasmic retention of TFEB/TFE3 occurs via a non‐canonical mechanism involving the RagC‐dependent recruitment of TFEB/TFE3 to the lysosome (Martina & Puertollano, 2013; Puertollano *et al*, 2018; Napolitano *et al*, 2020). We show here that the lysosomal localisation and protein expression levels of endogenous Rag GTPases are not significantly altered in senescence (Fig EV5A–D). Overexpression of active, RagC^75L^ was however able to rescue the nuclear accumulation of TFEB/TFE3 (Fig EV5E and F) which may indicate that nucleotide loading (or cycling) of RagC may be perturbed. Interestingly however, like p16/INK4a shRNA, there was no significant rescue of TFEB/TFE3 phosphorylation upon RagC^75L^ overexpression (Fig EV5G–I).

Together, these results are intriguing and difficult to reconcile with the current model of TFEB/TFE3 regulation. We observed that both p16/INK4a shRNA and RagC^75L^ overexpression were able to significantly reduce the percentage of cells with nuclear localisation of TFEB/TFE3, and in the case of p16/INK4a shRNA, this partially restored nutrient‐responsive flux of TFE3 between the nucleus (starved) and cytoplasm (fed) (Fig EV5J and K). It is not clear however what is preventing robust re‐phosphorylation of cytoplasmic TFEB/TFE3 in senescence in these conditions and requires further investigation.

**Figure 5:**
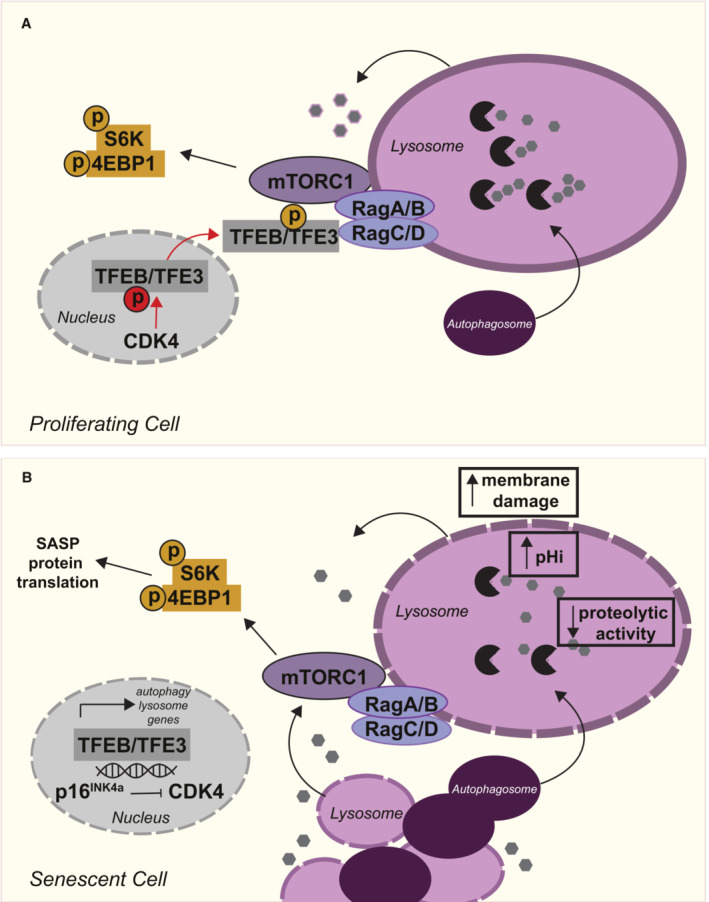
Summary In proliferating cells, TFEB‐dependent expression of autophagy‐lysosome genes is regulated by multiple phosphorylation events that regulate the subcellular localisation of TFEB/TFE3.Cellular senescence is associated with an increase in the autophagy‐lysosome pathway at the mRNA and protein level. We demonstrate that lysosomes in senescence are less efficient; they have increased pH and show signs of damage. Our data indicate that compensatory upregulation of lysosome biogenesis is driven by TFEB/TFE3. Experimental evidence suggests that dysregulation of multiple pathways may contribute to the hypo‐phosphorylation and constitutive nuclear localisation of TFEB/TFE3 in the nucleus, including p16‐CDK4 and RagC‐mTORC1.

Altogether, our data indicate that lysosomes in senescence are less active and show increased damage markers, but there is increased lysosome biogenesis which supports catabolic capacity comparable to control cells. We have demonstrated that the transcription factors, TFEB/TFE3 are constitutively nuclear in all forms of senescence, and therefore represent a new fundamental hallmark of senescence (Fig 5). Knockdown of these transcription factors reduced markers of the autophagy‐lysosome pathway and senescence phenotypes (including mTORC1 activity and secretion of IL6). Importantly, we have shown for the first time that targeting the lysosomes, either via lysosomal membrane damaging agents or knock‐down of TFEB/TFE3 causes senescent cell death, and thus may represent a novel target in the development of senolytics. We further observed that TFEB/TFE3 are dephosphorylated, and their subcellular localisation does not respond to changes in nutrient availability. Mechanistically, our evidence suggests that dysregulation of multiple pathways contributes to the nuclear accumulation of TFEB/TFE3, including p16/INK4a‐CDK4 and RagC‐mTORC1 (Fig 5).

## Discussion

Increased lysosomal content is an established hallmark of senescence both *in vitro* and *in vivo* (Lopez‐Otin *et al*, 2013; Hernandez‐Segura *et al*, 2018). Given the ever‐growing recognition of the importance of the lysosome for integrating mitogenic and stress signals (Ballabio & Bonifacino, 2020; Liu & Sabatini, 2020), remarkably little is known about the causes and consequences of the gross changes in lysosomal compartments in senescence (Carroll & Korolchuk, 2018). We show here that despite an increase in the mRNA (Young *et al*, 2009) and protein levels of lysosomal proteins, the proteolytic activity of lysosomes in senescent cells is reduced. Increased lysosomal biogenesis is however able to compensate to maintain the proteolytic capacity of senescent cells to a level remarkably similar to proliferating cells. This suggests that cells contain mechanisms that are able act as a rheostat to sense the minimal catabolic requirements and respond accordingly to ensure catabolism is maintained within extremely tight set‐points. These mechanisms are maintained in senescence, and increased lysosome biogenesis ensures continued protein degradation and autophagic flux. We and others have shown that autophagic flux is required in senescence for the liberation of amino acids to maintain mTORC1 signalling and mTORC1‐dependent senescence phenotypes such as translation of SASP factors (e.g. IL6) (Demidenko & Blagosklonny, 2008; Narita *et al*, 2011; Carroll *et al*, 2017). We have shown previously that inhibition of autophagy in the absence of exogenous nutrients causes senescent cell death (Carroll *et al*, 2017), which we can further confirm in this report, where loss of TFEB/TFE3‐dependent biogenesis of the autophagy‐lysosome pathway also promotes senescent cell death. Together it points to a complete re‐wiring of senescent cell metabolism leading to a reliance on the autophagy‐lysosome pathway.

At present, it is not clear why lysosomes in senescent cells are less active, despite the gross increase in biogenesis. The increased incidence of galectin‐positive lysosomes indicates that the lysosomal membranes may be damaged, although the reason for this remains unknown. Lipid availability could be a limiting factor to producing robust lysosomal membranes in senescence; indeed, lipid demand is likely to be enhanced in senescence due to the increased cell and nuclear size as well as increased mitochondrial biogenesis. Alternatively, senescence is widely associated with oxidative stress which could in theory cause protein and/or lipid damage. Notably, oxidative stress could also directly influence TFEB/TFE3 activation which have recently been shown to be redox sensitive (Martina *et al*, 2021), similar to other autophagy regulatory proteins (Carroll *et al*, 2018; Kataura *et al*, 2022). Furthermore, lysosomal stress impairs RagC‐dependent recruitment TFEB/TFE3 to lysosomes, promoting its nuclear accumulation (Nakamura *et al*, 2020; Goodwin *et al*, 2021). Another alternative explanation for increased lysosomal damage comes from our proteomic analysis which suggests that isolated senescent lysosomes accumulate non‐lysosomal proteins. Accumulation of undegraded cargo could cause membrane damage and further compound the dysregulation of lysosomal function. Further investigation of the enriched cargo in senescent lysosomes may reveal new insights into senescent cell biology.

Our data strongly indicate that biogenesis of the autophagy‐lysosome pathway in senescence is driven by TFEB/TFE3. Increased nuclear TFEB/TFE3 was observed in all models of senescence tested indicating this could be considered a new hallmark of senescence. It remains to be seen whether the same is true *in vivo* but given the current lack of effective biomarkers for senescence, this warrants future investigation. We identified that the subcellular localisation of TFEB/TFE3 becomes insensitive to nutrient availability in senescence, showing constitutive nuclear localisation in both fed and starved conditions. These data add to the growing evidence from ourselves and others that mitogenic signalling pathways, including mTORC1, are insensitive to changes in amino acids, growth factors and hormones in senescence, a phenotype that, as already mentioned, supports senescent cell metabolism and survival (Zhang *et al*, 2000; Narita *et al*, 2011; Carroll *et al*, 2017; Rabanal‐Ruiz *et al*, 2021).

Despite our best efforts, the mechanisms driving the nuclear accumulation of TFEB/TFE3 remain to be fully uncovered, lest to say they are hypo‐phosphorylated at multiple sites. Given the central role of mTORC1 signalling in driving a number of senescence phenotypes, it is interesting to note here that TFEB/TFE3, as non‐canonical mTORC1 substrates, are not phosphorylated in senescence. Our evidence suggests that knockdown of p16/INK4a and overexpression of active RagC can promote re‐localisation of TFEB to the cytoplasm and relieve senescence‐associated lysosomal phenotypes (Figs 4 and EV5). We could postulate that senescence‐associated defects in RagC nucleotide loading promote increased TFEB/TFE3 nuclear accumulation and the loss of CDK4 activity acts as a compounding factor, preventing phosphorylation and nuclear export. The net effect ultimately being constitutive localisation of TFEB/TFE3 in the nucleus. Neither intervention however was able to restore TFEB/TFE3 phosphorylation indicating additional mechanisms are preventing phosphorylation in the cytoplasm. As the molecular mechanisms regulating TFEB/TFE3 continue to be revealed, it will inevitably become clear in time exactly how these transcription factors are activated in senescence, and whether TFEB and/or TFE3 preferentially contribute to different senescence states and phenotypes. Indeed, oncogene‐induced senescence is strongly driven by p16/INK4a‐dependent exit from the cell cycle, while irradiation‐induced cell cycle arrest is more reliant on p53‐dependent p21/Rb pathway (van Deursen, 2014). In our assays, OIS is associated with a more robust nuclear accumulation of TFEB/TFE3 and greater increase in expression of autophagy‐lysosome genes. Furthermore, the impact of p16/INK4a shRNA was greater in OIS models compared to IR‐induced senescence. These indicate that different pathways may potentially contribute to TFEB/TFE3‐depedent lysosome biogenesis in senescence induced by different stressors.

An observation of note from this study was that the chronic loss of TFEB/TFE3 in proliferating, control cells displayed evidence of stress and hallmarks reminiscent of senescence, such as a loss of HMGB1, increased γH2AX‐positive DNA damage foci and reduced nuclear CDK4 (Appendix Fig S2). These seemingly contradictory roles, that is that TFEB/TFE3 may contribute, as well as protect against stress and senescence acquisition is consistent with the complex role autophagy has been noted to play in this context. Indeed, autophagy has been shown to be required for the mitotic transition to senescence (Young *et al*, 2009), while chronic inhibition of autophagy can promote senescence *in vivo* (Cassidy *et al*, 2020). The loss of HMGB1 occurs quickly (within 3 days) upon loss of TFEB/TFE3 and to a greater extent than that seen in senescence (Appendix Fig S2A). Such a dramatic loss of a chromatin‐binding protein like HMGB1 would have widespread effects on transcription. Consistent with previous conclusions, our data shows that TFEB/TFE3 control the expression of DNA damage response and cell cycle progression genes (Brady *et al*, 2018; Pisonero‐Vaquero *et al*, 2020) such as CDK4 (Appendix Fig S2), p16 and p21 (Brady *et al*, 2018; Pisonero‐Vaquero *et al*, 2020), and future investigations of TFEB/TFE3 knock‐down in primary cells may provide additional insights into how TFEB/TFE3 controls expression of specific subsets of genes (Brady *et al*, 2018).

## Materials and Methods

### Cell culture and drug treatments

HEK293T and primary human fibroblasts (MRC5) were cultured in DMEM (D6546) supplemented with 2 mM glutamine, 10% FBS and 100 U/ml penicillin–streptomycin at 37°C, 5% CO_2_. Human primary fibroblasts (IMR90) stably expressing inducible HRasV12 were cultured in phenol red‐free DMEM (31‐053‐028) supplemented with 2 mM glutamine, 20% FBS, 1 mM sodium pyruvate, 1× non‐essential amino acids and 100 U/ml penicillin–streptomycin at 37°C, 5% CO_2_ and 3% O_2_.

2.5 μM LY2835219 (CDK4 inhibitor/DMSO; Cambridge Bioscience CAY17740) for 3 h. Cells were incubated with 50 nM leptomycin B (LMB; #L2913) or 200 nM Torin1 (Tocris, #4247) for 2 h, followed where indicated by refeeding/washout which involved changing the media for fresh, complete media for a further 2 h. Cells were treated with 10 μM cyclosporin A (Tocris) for 2 h.

### Senescence induction

Four senescence models were used in this study; induced by (i) oncogene activation (oncogene‐induced senescence, OIS), (ii) ionising radiation (IR), (iii) DNA damaging agents, and (iv) replicative exhaustion (RepSen). To induce OIS, HRasV12 expression in IMR90 cells was induced by treatment of cells with 100 nM hydroxy‐tamoxifen for 6–8 days. MRC5 cells were exposed to 20Gy γ‐radiation (Gravatom RX30 irradiator with Cs137 source) to induce IR senescence. Immediately following exposure, media was replaced with fresh complete growth media which was changed every 3–4 days for 10–12 days. MRC5 were cultured until replication exhaustion and replicative senescence were defined by < 0.5 population doublings for at least 4 weeks. DNA damaging agents, doxorubicin (250 nM; Tocris #2252) and etoposide (100 μM; Tocris #1226) were incubated with MRC5 cells for 7 days.

### Viral production and shRNA

shRNAs in were purchased from Sigma‐Aldrich (human TFE3: TRCN0000232151; human TFE3: TRCN0000232154; human TFEB: TRCN0000440038; human TFEB: TRCN0000437246; human CDKN2A: TRCN0000281415; human CDKN2A: TRCN0000179873). GFP, GFP‐TFEB and FLAG‐TFEB were described previously (Curnock *et al*, 2019). RagC75L was subcloned from pRK5‐HA‐GST RagC 75L (Addgene #19305) and human CDK4 (synthesised as a gBlock gene fragment by IDT) to the lentiviral pXLG‐GFP plasmid using BamHI and KpnI restriction enzymes. Primers used were as follows: BamHI‐RagC TAAGCAGGATCCatgtccctgcagtacggggcggag; KpnI‐RagC TAAGCAGGTACCctagatggcgtttcgtggcgtgcca; BamHI‐CDK4 TAAGCAGGATCCatggctacctctcgatatgagcca; KpnI‐CDK4 TAAGCAGGTACCtcactccggattaccttcatcct.

HEK293T cells were transfected with plasmid containing gene of interest and packaging vectors pAX2 and pMDG2 for 48–60 h using polyethylenimine (PEI). Viruses were harvested, filtered and incubated with fibroblasts overnight. Media was changed the following day and cells cultured for the indicated amount of time.

### Nuclear fractionation and GFP immunoprecipitation

Cell nuclei were isolated using a standard REAP protocol as described previously (Hewitt *et al*, 2016). Briefly, cells from 10 cm dish were washed twice with ice‐cold PBS before being scraped into ice‐cold PBS and centrifuged briefly to pellet cells. The cell pellet was triturated 5× 0.1% NP‐40/PBS (whole cell lysate), followed by another brief spin. The supernatant was removed to a fresh tube and stored (cytoplasmic fraction). The pellet was resuspended in 0.1% NP‐40/PBS and triturated once. The sample was centrifuged briefly again. The nuclear pellet was resuspended in 0.1% NP40/PBS and sonicated. The samples were centrifuged 8,000 *g* for 30 s at 4°C before mixing with 10 μl GFP‐Trap beads and incubated with constant rotation for 2 h at 4°C. The IP buffer was supplemented with protease and phosphatase inhibitors. Beads were washed 3× IP buffer before analysis by Western blot.

### Immunoblotting

Immunoblotting was carried out as described previously (Carroll *et al*, 2017). Briefly, cells were lysed in RIPA buffer (50 mM Tris–HCl, pH 7.4, 150 mM NaCl, 1% NP‐40, 0.5% sodium deoxycholate, and 0.1% SDS, supplemented with Halt protease and phosphatase inhibitors; 1861280; Thermo Fisher Scientific) on ice. Lysates were cleared by centrifugation and protein concentration was measured using DC protein assay (500‐0112; Bio‐Rad Laboratories), and equal amounts of protein (20–30 μg) were subjected to SDS–PAGE (4–12% NuPAGE gradient gels) and immunoblotted. The following primary antibodies were used: rabbit anti‐p16 (18769, 1:1,000), rabbit anti‐Cathepsin B (31718, 1:2,000) rabbit anti‐NDP52 (D1E4A, 60732, 1:1,000), rabbit anti‐optineurin, rabbit anti‐HMGB1 (D3E5, 6893, 1:1,000), rabbit anti‐TFEB (E5P9M, 83010), rabbit anti‐CDK4 (D9G3E, 12790, 1:1,000) rabbit anti‐p21 (2947, 1:1,000), rabbit anti‐LC3B (3868, 1:2,000) and mouse anti‐GAPDH (97166, 1:1,000), all purchased from Cell Signalling Technology. Other antibodies used in this study include rabbit anti‐TFE3 (HPA023881, Atlas Antibodies, 1:1,000), rabbit anti‐phospho TFEB^S142^ (ABE1971, Sigma‐Aldrich, 1:1,000), rabbit anti‐CRM1 (Bethyl Laboratories, A300‐469A, 1:500) mouse anti‐α‐tubulin (12G10, 1:10,000; Developmental Studies Hybridoma Bank, DSHB), mouse anti‐Lamp1 (H4A3, DSHB, 1:2,000), guinea pig anti‐p62 (610832, Becton Dickinson, 1:2,000). Secondary antibodies conjugated to Alexa Fluor 680 or 800 were all used at 1:20,000 for 1 h at RT. Blots were imaged on LiCOR Odyssey xL (LiCOR) and quantified using ImageLite software (LiCOR).

### Immunofluorescence

Immunofluorescence was performed as previously described. Briefly, cells were fixed in 4% formaldehyde/PBS for 10 min at room temperature. Cells were permeabilised with 0.5% Triton X‐100 for 10 min/PBS at room temperature and blocked in 5% normal goat serum/PBS‐Tween 0.05% for 1 h at room temperature. Primary antibodies were diluted in blocking serum and incubated overnight at 4°C. Primary antibodies used in this study: mouse anti‐Lamp1 (for human cells, H4A3, DSHB, 1:2,000), mouse anti‐Lamp2 (H4B4, DSHB, 1:2,000), mouse anti‐p62 (GP62‐C, Progen Biotechnik, 1:500), mouse anti‐phospho γH2AX (JBW301, 05‐636, Sigma‐Aldrich, 1:5,000), rabbit anti‐TFE3 (HPA023881, Atlas Antibodies, 1:500), rabbit anti‐CDK4 (D9G3E, 12790, 1:200), rabbit anti‐HMGB1 (D3E5, 6893, 1:200) and rabbit anti‐Galectin 1 (ab25138, Abcam). Cells were washed and incubated with the appropriate Alexa Fluor‐conjugated secondary antibodies (1:1,000; Thermo Fisher Scientific) for 1 h at room temperature. Coverslips were mounted using Prolong Gold with DAPI. Confocal images were collected on Leica SP5‐II AOBS confocal laser scanning microscope using 63× HCX Plan‐Apo/1.3‐NA oil objective. Cell viability probes (Thermo Scientific, #R37601) were imaged on Leica DMI6000 epifluorescence microscope. All analysis were performed using ImageJ (Fiji) software. In all cases, a constant threshold was applied to all cells within an experiment. For presentation purposes, images in the manuscript are maximum projections of z‐stacks and brightness/contrast has been adjusted in ImageJ (again, this is constant across all images within an experiment). Where particle analysis was employed, a constant threshold was applied to images and quantified using “Analyse particle” plugin. Particle size was defined as 5‐infinity. In all cases, at least 20 cells (or 4 fields of view) per condition per experimental repeat were quantified. Scoring of TFEB/TFE3‐positive nuclei was carried out by eye after images were blinded to the scorer.

### RNA extraction, reverse transcription, and quantitative PCR

Total RNA was extracted from cells using RNeasy Mini Kit (Qiagen). cDNA was synthesised and real‐time quantitative RT–PCR was performed using the SuperScript III Platinum SYBR Green One‐Step quantitative RT–PCR Kit (Thermo Fisher Scientific). The quantification of gene expression was performed in triplicate. Amplification of the sequence of interest was normalised with a housekeeping gene, PUM1. Quantification was performed using comparative threshold cycle (Ct) method of analysis. The value was expressed as a fold change relative to RNA from proliferating cells.

### Analysis of lysosome activity

Lysosomal proteolytic activity was assessed by flow cytometry using the fluorogenic protease substrate DQ Green BSA and the cathepsin D probe BODIPY FL pepstatin A. Proteolytic activity was normalised to lysosomal content using Texas Red‐BSA. Cells were seeded into 6 well culture plate and 48 h later cells were incubated in complete media containing 10 μg/ml of DQ Green BSA for 4 h, 1 μM of BODIPY FL pepstatin A for 30 min, Magic Red Cathepsin B for 2 h or 100 μg/ml Texas Red‐BSA for 4 h. Unlabelled cells were used to assess autofluorescence. Following labelling, cells were washed in PBS (without Ca^2+^ or Mg^2+^) and then incubated for 20 min with Accutase to detach cells. Resuspended cells were subsequently transferred to a 5 ml round‐bottomed polystyrene tubes (BD Biosciences). Cells were reconstituted in 100 μl CO_2_‐independent media supplemented with 5% FCS and DRAQ7 (1/100). Fluorescence signals from 10,000 cells were measured using a BD LSR Fortessa X20 or ACEA Novocyte 3000 analyser. Data were analysed using Flowjo software (Flowjo, Ashland, OR). Lysosomal proteolytic activity was visualised by incubating cells with 10 μg/ml of DQ Green BSA and 100 μg/ml Texas Red‐BSA for 4 h prior to fixation in 4% formaldehyde/PBS and subsequent examination by confocal microscopy.

To measure lysosomal pH, cells were seeded into a 96‐well plate before incubation with 1 μM LysoSensor Yellow/Blue for 5 min. Cells were washed once and then media was either replaced for 10 min with DMEM (21063‐029) supplemented with 1 mM sodium pyruvate and 10% FBS for test wells, or buffer (25 mM HEPES, 115 mM KCl, 1.2 mM MgCl2, 10 mM glucose, 10 μM nigericin) calibrated to pH 4.5, 5.0, 5.5, 6.0, 6.5 and 7.0 to generate a standard curve (Liu *et al*, 2018). The plate was read in a SectraMax ID3 with Excitation and emission filters: 329/440 and 380/540.

### Lysosome isolation and LC–MS

Cells were cultured in SILAC media (L‐Lysine‐2HCl, ^13^C6 ^15^N2 and L‐Arginine‐HCl, ^13^C6 ^15^N4) (Thermo Fisher Scientific) for at least 3 weeks prior to experiments. Lysosomes were isolated using the Iron‐Dextran (FeDex) protocol as previously described (Diettrich *et al*, 1998). Cells were cultured in 15 cm diameter plates until ~80% confluent. Cells were incubated with fresh FeDex solution (volume batch optimised) overnight. The next day, *in vivo* cross‐linking was carried out by incubating cells with 1 mg/ml DSP for 10 min at 37C, 5% CO2, followed by quenching for 10 min with Tris–HCl (pH 8.0). Cells were then washed once in cold PBS on ice before being scrapped into 2 ml SCA buffer (20 mM Hepes‐KOH, pH 7.5, 250 mM sucrose, 1.5 mM MgCl2, 1 mM EGTA, 1 mM EDTA, 1 × protease/phosphatase inhibitor, 1 mM DTT). Cells were centrifuged for 5 min at 300 *g*. Cells were lysed using Gentle MACs Dissociator (Miltenyi Biotec) and centrifuged 2× 10 min 1,000 *g* at 4°C. The light membrane fraction (LMF) supernatant was applied to a previously equilibrated LS MiniMachs column (130‐042‐401, Miltenyi Biotec) on a magnetic stand. Following three washes with 1 ml SCA buffer (without protease inhibitors), the lysosomes were eluted by removal of the column from the magnet and addition of SCA buffer (with plunger). Samples were subjected to a final centrifugation at 20,000 *g* for 20 min. The pelleted lysosomal fractions were subjected to LC–MS‐based proteomics.

### LC–MS‐based proteomics

The SILAC‐labelled protein samples were analysed by SDS–PAGE and the gel lane was cut into four fractions to reduce the proteome complexity. The LC–MS proteomic procedures were performed as previously described (Edhager *et al*, 2018). The gel pieces were treated by in‐gel trypsin digestion followed by peptide C18 spin column purification. Identification and quantification of the peptide mixtures were performed by nano‐liquid chromatography, nanoLC (Easy‐nanoLC 1200, Thermo Scientific) coupled to tandem mass spectrometry, MS/MS (Q‐Exactive Plus, Thermo Scientific, Bremen). Precolumn (Acclaim PepMap 100, 75 μm × 2 cm, nanoViper, Thermo Scientific) and analytical column (EASY‐Spray column, PepMap, C18, 2 μm, 100 Å, 75 μM × 25 cm), respectively, were used to trap and separate peptides using a 90 min gradient (5–40% acetonitrile, 0.1% formic acid). All spectra were collected in positive mode. Mass resolution was set at 70,000 (at 200 *m*/*z*) and 17,500 in full scan (MS1) and fragment scan (MS2), respectively. Scan range was 392–1,800 *m*/*z*. Data‐dependent analysis (DDA) was applied using higher energy collisional dissociation (HCD) fragmentation with normalised collision energy of 29. Up to 10 of the most intense peaks in MS1 were fragmented, and dynamic exclusion was set to 14 s, and unassigned and +1 charge states were excluded.

Identification and quantification of the SILAC‐labelled proteins were carried out in MaxQuant (version 1.5.3.30). Mass spectra were searched against a sequence database; fasta file with 20,149 reviewed *Homo sapiens* sequences downloaded from Uniprot.org 12/12/2016.

Ratio between heavy (containing either +8 Da Lysine (K) or +10 Da Arginine (R)) and light SILAC peptides was calculated and applied for relative quantification. Carbamidomethylation (CAM) of cysteines was set as static modification and oxidation of methionine and as variable modification. Peptide and protein false discovery rate (FDR) of identification were set to 0.02 and 0.01, respectively.

## Author contributions

**Bernadette Carroll:** Conceptualization; formal analysis; supervision; funding acquisition; investigation; visualization; writing – original draft; project administration; writing – review and editing. **Rachel Curnock:** Formal analysis; investigation; visualization; writing – review and editing. **Katy Yalci:** Formal analysis; visualization; writing – review and editing. **Johan Palmfeldt:** Formal analysis; investigation; visualization; writing – review and editing. **Marja Jäättelä:** Supervision; writing – review and editing. **Bin Liu:** Formal analysis; investigation; visualization; writing – review and editing.

## Disclosure and competing interests statement

The authors declare that they have no conflict of interest.

## Supporting information



Appendix

Expanded View Figures PDF

Dataset EV1

Source Data for Expanded View

PDF+

Source Data for Figure 1

Source Data for Figure 3

Source Data for Figure 4

## Data Availability

Data from proteomic analysis of isolated lysosomes submitted to PRIDE (accession: PXD034358; Name: Lysosomal alterations in senescence)
