# Supplementary material for: TFEB‐dependent lysosome biogenesis is required for senescence
Source: EMBO J. 2023 Mar 27;42(9):e111241. doi: 10.15252/embj.2022111241 (PMC10152146; doi:10.15252/embj.2022111241)
Supplement: Supplementary file 1 — Appendix [file EMBJ-42-e111241-s001.pdf]

TFEB-dependent lysosome biogenesis is required for senescence

Rachel Curnock, Katy Yalci, Johan Palmfeldt, Marja Jaattela, Bin Lui, Bernadette Carroll

Appendix Contents

	PAGE NUMBER
Appendix Figure S1	2
Appendix Figure S2	4
Appendix Figure S3	6
Appendix Figure S4	8
Appendix Figure S5	10

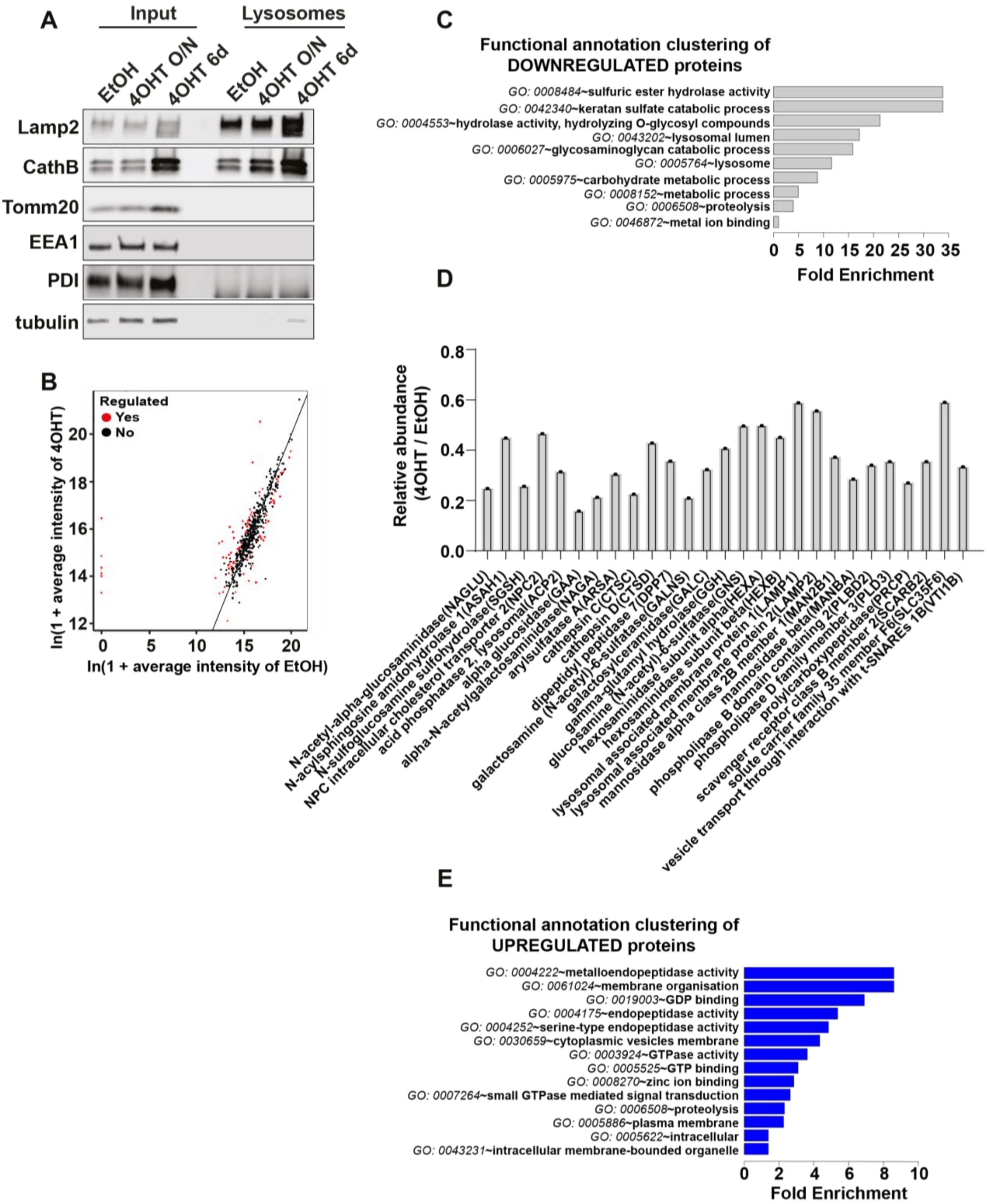
A
Input
Lysosomes
EtOH
4OHT O/N
4OHT 6d
EtOH
4OHT O/N
4OHT 6d
Lamp2
CathB
Tomm20
EEA1
PDI
tubulin
B
Regulated
Yes
No
ln(1 + average intensity of 4OHT)
ln(1 + average intensity of EtOH)
12
14
16
18
20
0
5
10
15
20
C
Functional annotation clustering of
DOWNREGULATED proteins
GO: 0008484~sulfuric ester hydrolase activity
GO: 0042340~keratan sulfate catabolic process
GO: 0004553~hydrolase activity, hydrolyzing O-glycosyl compounds
GO: 0043202~lysosomal lumen
GO: 0006027~glycosaminoglycan catabolic process
GO: 0005764~lysosome
GO: 0005975~carbohydrate metabolic process
GO: 0008152~metabolic process
GO: 0006508~proteolysis
GO: 0046872~metal ion binding
0 5 10 15 20 25 30 35
Fold Enrichment
D
Relative abundance
(4OHT / EtOH)
0.0
0.2
0.4
0.6
0.8
N-acetyl-alpha-glucosaminidase(NAGLU)
N-acylsphingosine amidohydrolase 1(ASAH1)
N-sulfoglucosamine sulfohydrolase(SGSH)
NPC intracellular cholesterol transporter 2(NPC2)
acid phosphatase 2, lysosomal(ACP2)
alpha glucosidase(GAA)
alpha-N-acetylgalactosaminidase(NAGA)
arylsulfatase A(ARSA)
cathepsin C(CTSC)
cathepsin D(CTSD)
dipeptidyl peptidase 7(DPP7)
galactosamine (N-acetyl)-6-sulfatase(GALNS)
galactosylceramidase(GALC)
gamma-glutamyl hydrolase(GGH)
glucosamine (N-acetyl)-6-sulfatase(GNS)
hexosaminidase subunit alpha(HEXA)
hexosaminidase subunit beta(HEXB)
lysosomal associated membrane protein 1(LAMP1)
lysosomal associated membrane protein 2(LAMP2)
mannosidase alpha class 2B member 1(MAN2B1)
mannosidase beta(MANBA)
phospholipase B domain containing 2(PLBD2)
phospholipase D family member 3(PLD3)
prolylcarboxypeptidase(PRCP)
scavenger receptor class B member 2(SCARB2)
solute carrier family 35 member F6(SLC35F6)
vesicle transport through interaction with t-SNAREs 1B(VTI1B)
E
Functional annotation clustering of
UPREGULATED proteins
GO: 0004222~metalloendopeptidase activity
GO: 0061024~membrane organisation
GO: 0019003~GDP binding
GO: 0004175~endopeptidase activity
GO: 0004252~serine-type endopeptidase activity
GO: 0030659~cytoplasmic vesicles membrane
GO: 0003924~GTPase activity
GO: 0005525~GTP binding
GO: 0008270~zinc ion binding
GO: 0007264~small GTPase mediated signal transduction
GO: 0006508~proteolysis
GO: 0005886~plasma membrane
GO: 0005622~intracellular
GO: 0043231~intracellular membrane-bounded organelle
0 2 4 6 8 10
Fold Enrichment

Appendix Figure S1: Lysosomes from senescent cells are associated with an accumulation of non-lysosomal proteins.

A Representative blot of lysosomal isolations from EtOH and 4OHT-treated cells using FedEX. O/N = overnight; 6d = 6 days.

B The mean intensity of each protein present in isolated lysosomes from EtOH-treated cells plotted against the mean intensity from lysosomes in 4OHT-treated cells (both transformed by ln(1+x)). Nine proteins were identified in 4OHT only (data points to the far most left of the plot). The line shown in the plot is y=x; meaning both conditions have the same intensities. Red coloured data points are the differentially regulated proteins in the analysis. (n=3 independent experimental repeats)

C Functional groups of proteins downregulated in lysosomes isolated from 4OHT-treated cells. (n=3 independent experimental repeats)

D Relative abundance of common lysosomal proteins in lysosomal preps isolated from 4OHT-treated cells, relative to EtOH controls. (n=3 independent experimental repeats)

E Functional groups of proteins upregulated in lysosomes isolated from 4OHT-treated cells. (n=3 independent experimental repeats)

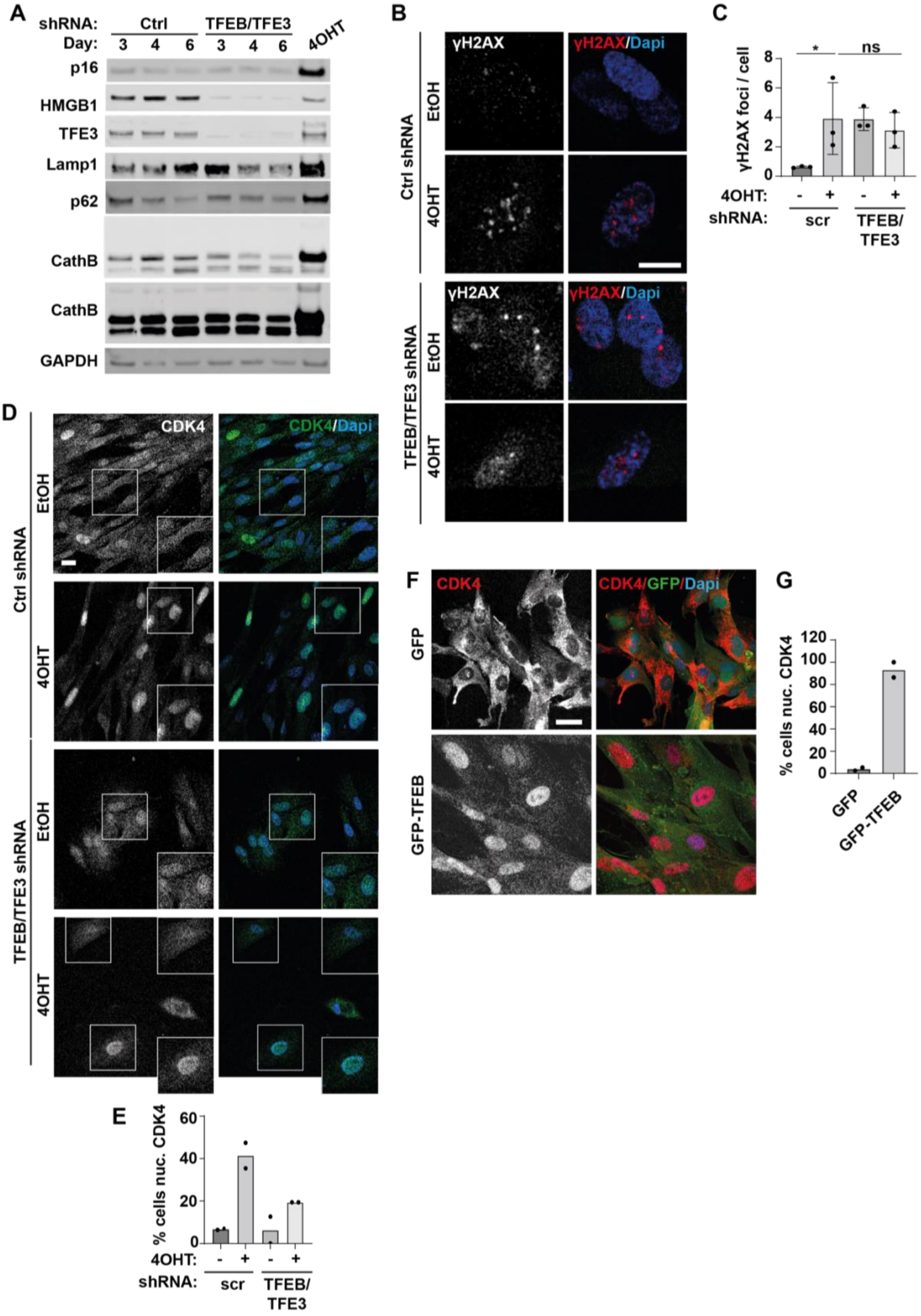
A
shRNA: Ctrl TFEB/TFE3
Day: 3 4 6 3 4 6 4OHT
p16
HMGB1
TFE3
Lamp1
p62
CathB
CathB
GAPDH
B
Ctrl shRNA
TFEB/TFE3 shRNA
EtOH
4OHT
γH2AX
γH2AX/Dapi
C
γH2AX foci / cell
*
ns
4OHT: - + - +
shRNA: scr TFEB/TFE3
D
CDK4
CDK4/Dapi
Ctrl shRNA
TFEB/TFE3 shRNA
EtOH
4OHT
E
% cells nuc. CDK4
4OHT: - + - +
shRNA: scr TFEB/TFE3
F
CDK4
CDK4/GFP/Dapi
GFP
GFP-TFEB
G
% cells nuc. CDK4
GFP
GFP-TFEB

Appendix Figure S2: TFEB/TFE3 shRNA induces signs of stress.

A Proliferating fibroblasts were transduced with TFEB and TFE3 shRNA for the indicated amount of time. Cells were lysed and subject to Western blotting.

B Fibroblasts were transduced with TFEB and TFE3 shRNA 3days after induction of senescence by 4OHT, fixed and immunostained for DNA damage foci (γH2AX). Scale bar: 10µm.

C Quantification of B. (n=3 independent experimental repeats (at least 100 cells analysed from at least 5 fields of view per repeat)

D Fibroblasts were transduced with TFEB and TFE3 shRNA 3days after induction of senescence by 4OHT, fixed and immunostained for endogenous CDK4. Scale bar: 20µm;

E Quantification of D. (n=2 independent experimental repeats (at least 100 cells analysed from at least 5 fields of view per repeat)

F Immunostaining for endogenous CDK4 in cells expressing GFP or GFP-TFEB. Scale bar: 20µm.

G Quantification of F. (n=2 independent experimental repeats (at least 30 cells analysed from at least 3 fields of view per repeat)

Data information: All graphs show individual data points, mean and error bars represent standard deviation. All data (where n=3) analysed by one-way ANOVA with Tukey's multiple comparison test (* <0.05)

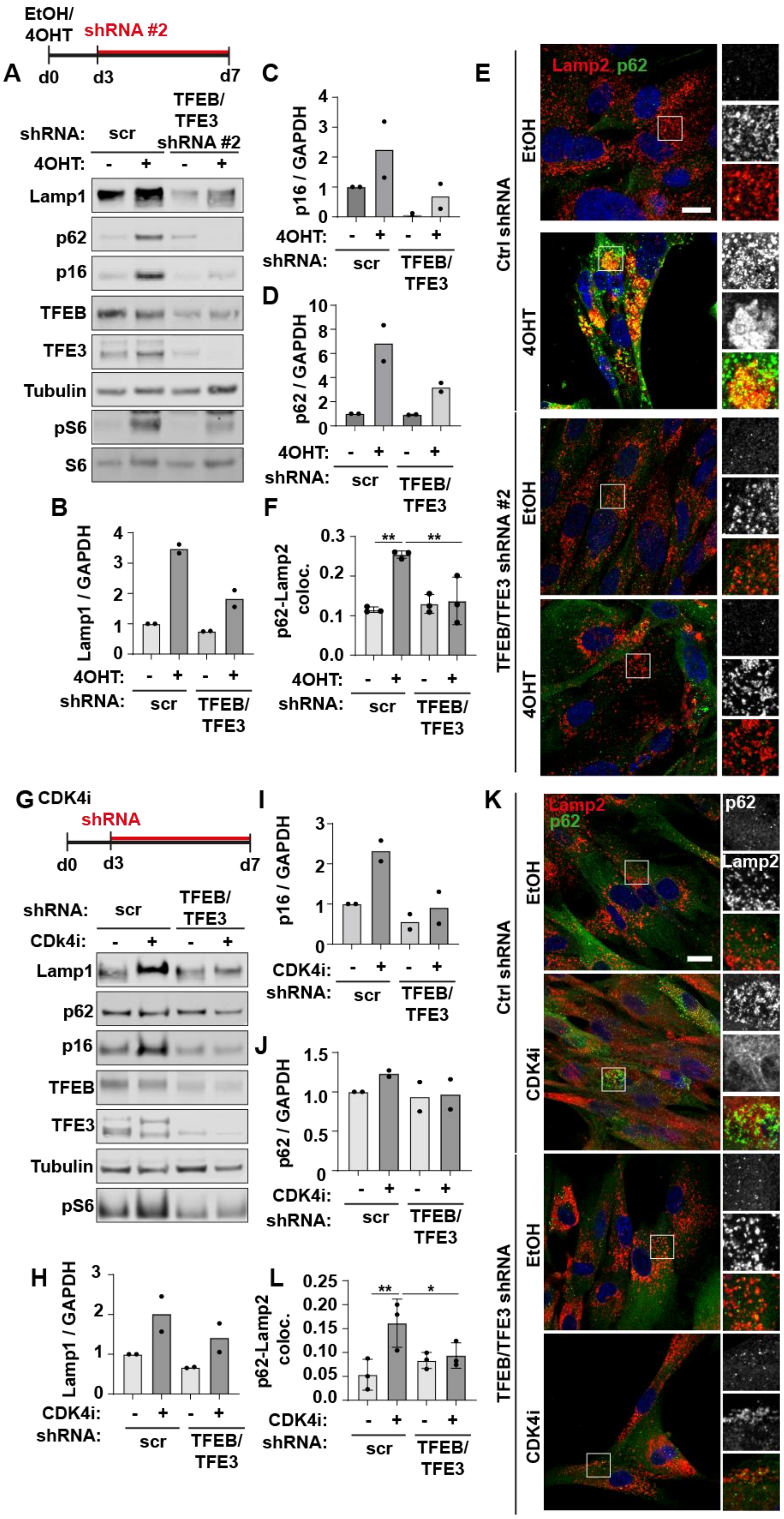

A
EtOH/
4OHT
shRNA #2
d0
d3
d7
shRNA:
scr
TFEB/
TFE3
shRNA #2
4OHT:
Lamp1
p62
p16
TFEB
TFE3
Tubulin
pS6
S6
B
Lamp1 / GAPDH
4OHT:
shRNA:
scr
TFEB/
TFE3
C
p16 / GAPDH
4OHT:
shRNA:
scr
TFEB/
TFE3
D
p62 / GAPDH
4OHT:
shRNA:
scr
TFEB/
TFE3
F
p62-Lamp2
coloc.
**
**
4OHT:
shRNA:
scr
TFEB/
TFE3
E
Lamp2 p62
Ctrl shRNA
EtOH
4OHT
TFEB/TFE3 shRNA #2
EtOH
4OHT
G
CDK4i
shRNA
d0
d3
d7
shRNA:
scr
TFEB/
TFE3
CDK4i:
Lamp1
p62
p16
TFEB
TFE3
Tubulin
pS6
H
Lamp1 / GAPDH
CDK4i:
shRNA:
scr
TFEB/
TFE3
I
p16 / GAPDH
CDK4i:
shRNA:
scr
TFEB/
TFE3
J
p62 / GAPDH
CDK4i:
shRNA:
scr
TFEB/
TFE3
L
p62-Lamp2
coloc.
**
*
CDK4i:
shRNA:
scr
TFEB/
TFE3
K
Lamp2
p62
p62
Lamp2
Ctrl shRNA
EtOH
CDK4i
TFEB/TFE3 shRNA
EtOH
CDK4i

Appendix Figure S3: TFEB/TFE3 drives lysosomal biogenesis in senescence

A Fibroblasts were transduced with a second TFEB and TFE3 shRNA (compared to Fig.3) 3days after induction of senescence by 4OHT. Cells were lysed and analysed by Western blotting.

B,C,D Quantification of blots as indicated. (n=2 independent experimental repeats)

E Cells treated as in A were fixed and immunostained for Lamp2 and p62. Scale bar: 20µm;

F Quantification of E; p62-Lamp2 colocalisation (Mander's coefficient). (n=3 independent experimental repeats)

G Fibroblasts were transduced with TFEB and TFE3 shRNA (same as in Fig.3) 3days after induction of senescence by CDK4i and analysed by Western blot. (n=2 independent experimental repeats)

H,I,J Quantification of blots as indicated. (n=2 independent experimental repeats)

K Cells treated as in G were fixed and immunostained for Lamp2 and p62. Scale bar: 20µm; (n=3 independent experimental repeats)

L Quantification of p62-Lamp2 colocalisation (Mander's coefficient). (n=3 independent experimental repeats)

Data information: All graphs show individual data points, mean and error bars represent standard deviation. All data (where n=3) analysed by one-way ANOVA with Tukey's multiple comparison test (* <0.05, ** <0.01)

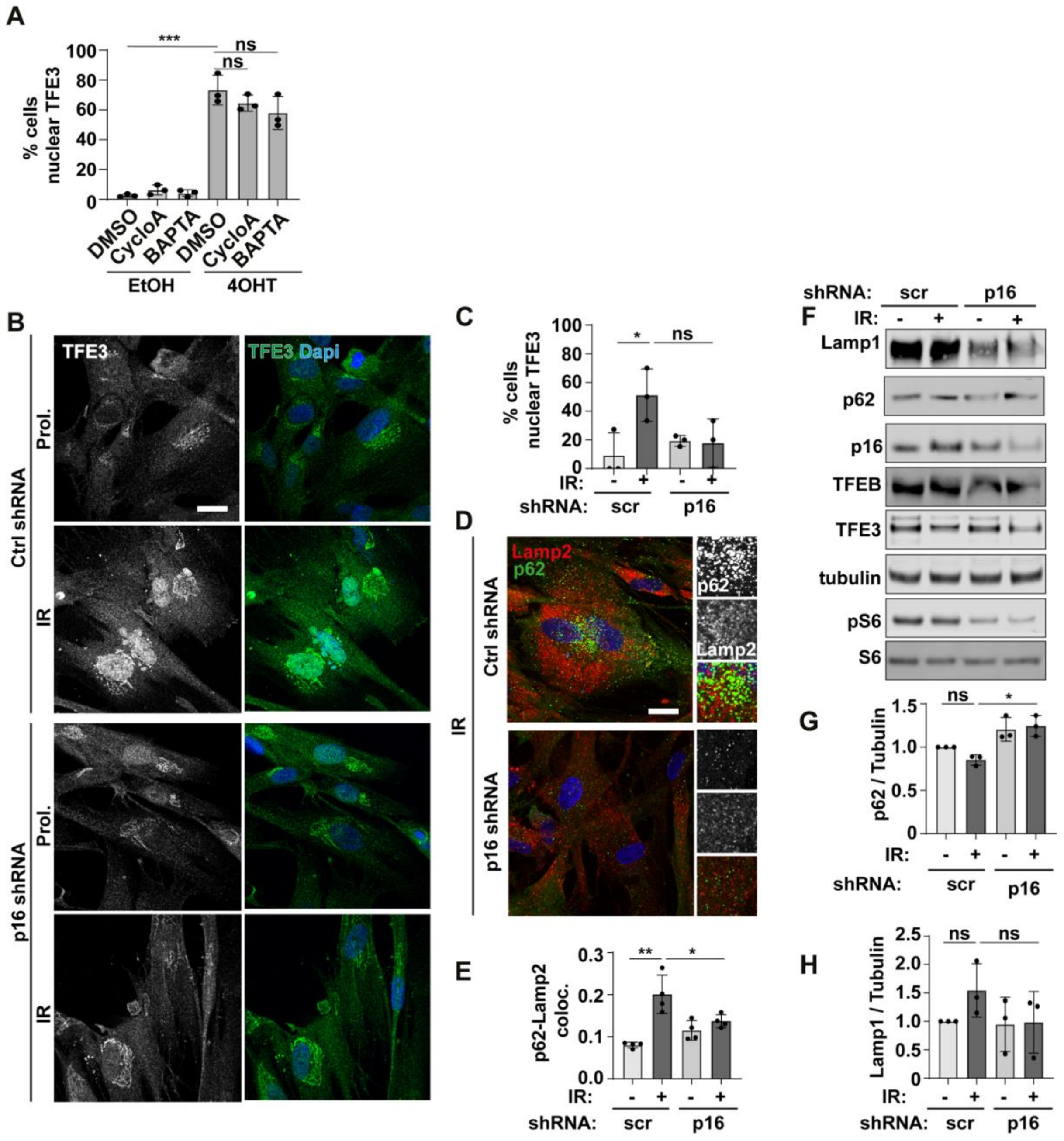

Appendix Figure S4: Senescence-associated p16^{INK4a} contributes to nuclear accumulation of TFEB/TFE3.

A EtOH and 4OHT cells were treated with cyclosporin A or BAPTA-AM (2 hours) as indicated, fixed and immunostained for endogenous TFE3. Nuclear TFE3 was quantified. (n=3 independent repeats (at least 60 cells analysed from at least 4 fields of view per repeat)

B Fibroblasts were treated with p16 shRNA immediately following ionising radiation (IR). Cells were fixed and immunostained for endogenous TFE3. Scale bar: 20µm.

C Quantification of B (n=3 independent repeats (at least 100 cells analysed from at least 8 fields of view per repeat).

D Cells treated as in B, fixed and immunostained for Lamp2 and p62. Scale bar: 20µm

E Quantification of D, colocalisation (Mander's coefficient) of Lamp2 and p62 (n=4 independent experimental repeats (at least 40 cells analysed from at least 4 fields of view per repeat).

F Cells treated as in B, lysed and subject to Western blotting.

G, H Quantification of F (n=3 independent experimental repeats)

Data information: All graphs show individual data points, and error bars represent standard deviation. Data analysed (where n=3) by one-way ANOVA with Tukey's multiple comparison test (* <0.05, ** <0.01, *** <0.001)

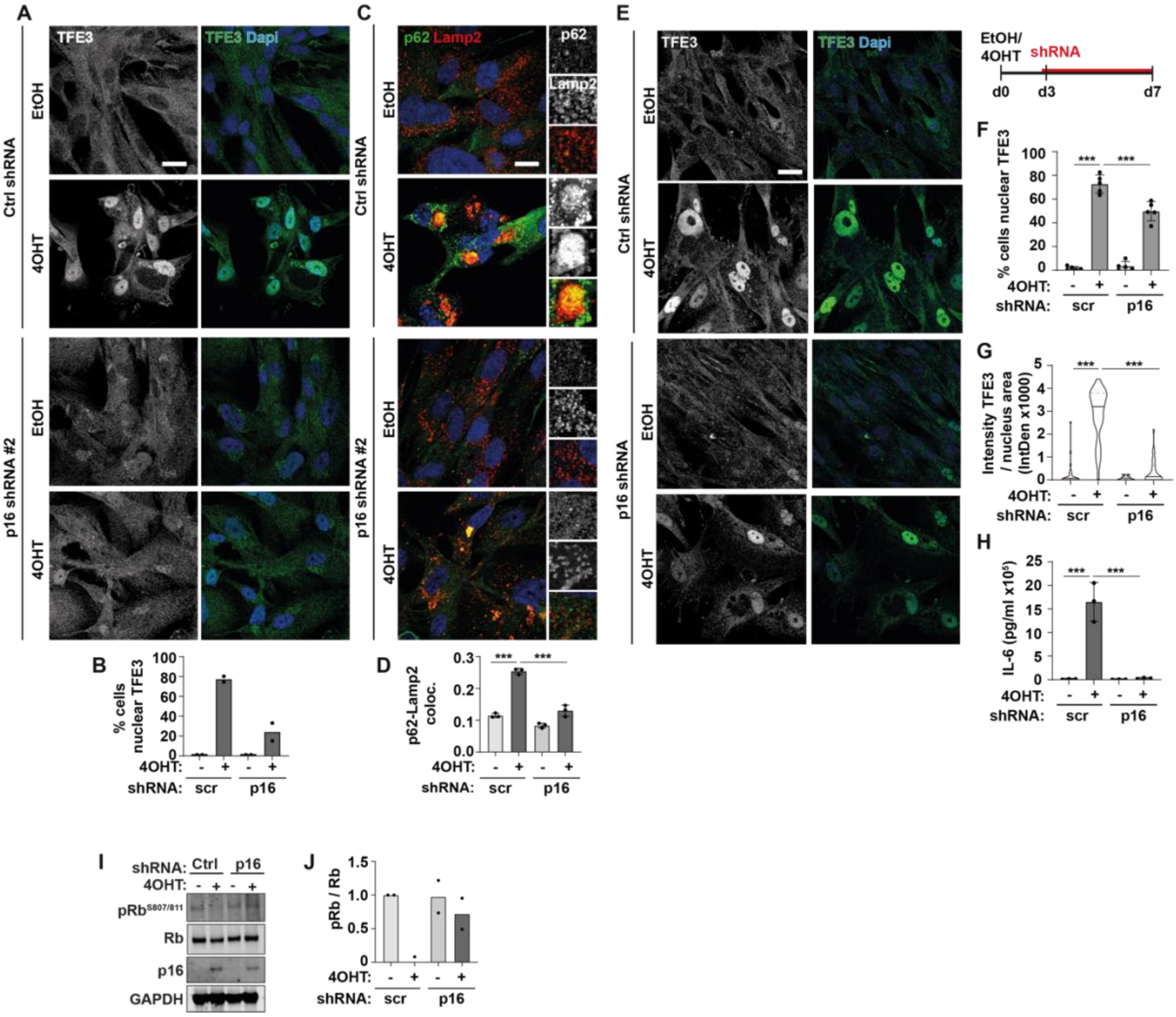

Appendix Figure S5: Senescence-associated p16^{INK4a} contributes to nuclear accumulation of TFEB/TFE3.

A Fibroblasts were transduced with a second p16 shRNA (compared to Figure 4) simultaneously with induction of senescence by 4OHT. Cells were fixed and immunostained for endogenous TFE3.

B Quantification of A. (n=2independent experimental repeats (at least 150 cells analysed from at least 5 fields of view per repeat).

C Fibroblasts were treated as in A, fixed and immunostained for p62 and Lamp2.

D Quantification of C, co-localisation (Mander's coefficient) of p62 and Lamp2 (n=3 independent experimental repeats (at least 40 cells analysed from at least 4 fields of view per repeat).

E Fibroblasts were transduced with p16 shRNA (the same one used in Figure 4) 3days after the induction of senescence by 4OHT. Cells were fixed and immunostained for endogenous TFE3.

F Quantification of E (n=5 independent experimental repeats (at least 80 cells analysed from at least 4 fields of view per repeat).

G Quantification of E, including all data points from 5 independent experimental repeats to demonstrate the spread of data (n=5 independent experimental repeats).

H Cells treated as in E were incubated with serum-free media overnight of day 6 to 7. Media was collected the next morning and subject to an ELISA assay to measure IL-6 secretion.

I Fibroblasts were transduced with p16 shRNA (same as Figure 4) simultaneously with induction of senescence by 4OHT. Cells were lysed and subject to Western blotting.

J Quantification of I (n=2 independent experimental repeats)

Data information: All bar graphs show individual data points, and error bars represent standard deviation. Violin blots include all datapoints from n=5, lines represent median and upper and lower quartiles; Data analysed by one-way ANOVA with Tukey's multiple comparison test (*** <0.001).
