# Supplementary material for: TFEB‐dependent lysosome biogenesis is required for senescence
Source: EMBO J. 2023 Mar 27;42(9):e111241. doi: 10.15252/embj.2022111241 (PMC10152146; doi:10.15252/embj.2022111241)
Supplement: Supplementary file 2 — Expanded View Figures PDF [file EMBJ-42-e111241-s009.pdf]

Expanded View Figures

Figure EV1. Multiple models of senescence are associated with an increase in dysfunctional lysosomes.

A Senescence was induced in MRC5 primary fibroblasts by ionising γ-radiation (IR; 20Gy) and analysed 10 days later by Western blot and immunofluorescence. Blots and images were quantified and represented relative to proliferating (Prol) controls. Scale bar: 20 μm (n = 3 independent experimental repeats (at least 40 cells analysed from at least four fields of view per repeat)).

B Assays as described in (A) were carried out on Replicative Senescent (RS) MRC5 cells. Scale bar: 20 μm (n = 3 independent experimental repeats (at least 40 cells analysed from at least four fields of view per repeat)).

C Assays as described in (A) were carried out on MRC5 fibroblasts treated with etoposide or doxorubicin for 7 days. Scale bar: 20 μm (n = 2 independent experimental repeats (at least 40 cells analysed from at least four fields of view per repeat)).

D Assays as described in (A) were carried out on MRC5 fibroblasts treated with CDK4 inhibitor (CDK4i) every 24 h for 7 days. Scale bar: 20 μm (n = 2 independent experimental repeats (at least 40 cells analysed from at least four fields of view per repeat)).

E Cells were incubated with DQ-BSA (2 h), and signal was analysed by flow cytometry. Data are MFI represented relative to EtOH Control (n = 3 independent experimental repeats).

F Cells were incubated with BODIPY-Pepstatin A for 30 min, and signal was analysed by flow cytometry. Data are MFI represented relative to EtOH Control (n = 3 independent experimental repeats).

G Cells were incubated with Magic Red Cathepsin B (2 h), and signal was analysed by flow cytometry. Data are MFI represented relative to EtOH Control (n = 3 independent experimental repeats).

H Proliferating and replicative senescent cells were incubated with TR-BSA and analysed by flow cytometry. Data are MFI represented relative to EtOH Control (n = 2 independent experimental repeats).

I Proliferating and replicative senescent cells were incubated with DQ-BSA and analysed by flow cytometry. Data are MFI represented relative to EtOH Control (n = 2 independent experimental repeats).

J Data from (H and I) represented as a ratio of DQ-BSA/TR-BSA, relative to EtOH Control (n = 2 independent experimental repeats).

Data information: All graphs show individual data points, mean and error bars represent standard deviation. All data (where n = 3) are analysed by 2-tailed, nonpaired Student's t-test (*< 0.05).
Source data are available online for this figure.

 © 2023 The Authors

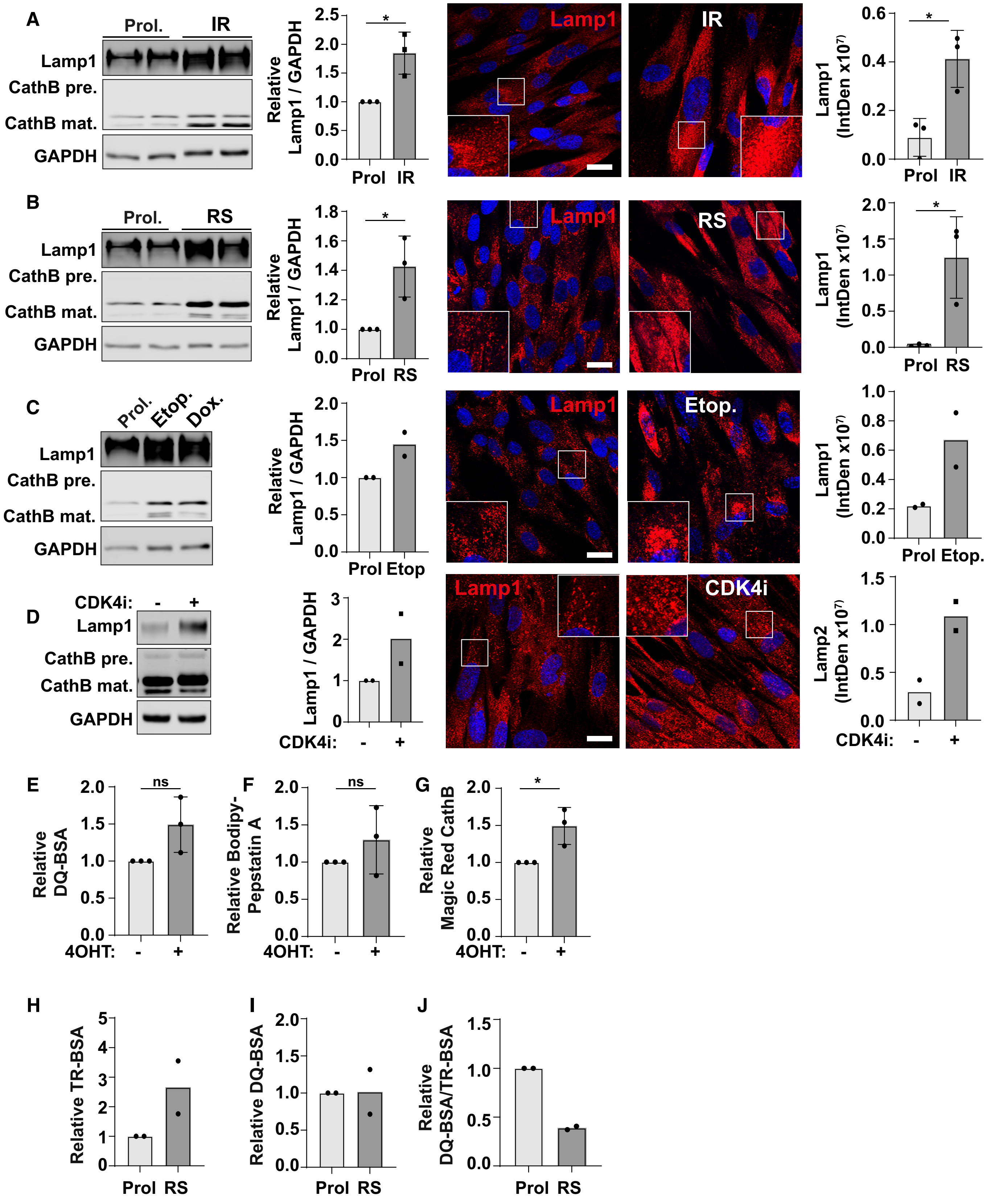
A
Prol.
IR
Lamp1
CathB pre.
CathB mat.
GAPDH
Relative Lamp1 / GAPDH
Prol IR
Lamp1
IR
Lamp1 (IntDen x10^7)
B
RS
C
Prol.
Etop.
Dox.
Prol Etop
Etop.
D
CDK4i:
CDK4i
Lamp2 (IntDen x10^7)
E
Relative DQ-BSA
4OHT:
F
Relative Bodipy-Pepstatin A
G
Relative Magic Red CathB
H
Relative TR-BSA
I
J
Relative DQ-BSA/TR-BSA
ns
*

Figure EV1.

Figure EV2. Targeting lysosomes in senescence promotes cell death.

A EtOH and 4OHT-treated fibroblasts were fixed, immunostained for Galectin 1 (Gal1) and Lamp1. The number of $Gal1^+$ puncta was quantified. Scale bar: 10 μm (n = 3 independent experimental repeats (at least 40 cells analysed from at least four fields of view per repeat)).

B Proliferating and IR fibroblasts were fixed, immunostained for Galectin 1 (Gal1) and Lamp1. The number of $Gal1^+$ puncta was quantified. Scale bar: 20 μm (n = 3 independent experimental repeats (at least 40 cells analysed from at least four fields of view per repeat)).

C EtOH and 4OHT-treated fibroblasts were incubated in the presence/absence of LLoMe for 2 h, immunostained with antibodies against Gal1 and Lamp1, and the number of $Gal1^+$ puncta was quantified. Scale bar: 20 μm (n = 4 independent experimental repeats (at least 40 cells analysed from at least four fields of view per repeat)).

D Proliferating and IR fibroblasts were incubated in the presence/absence of LloMe for 2 h, immunostained with antibodies against Gal1 and Lamp1, and the number of $Gal1^+$ puncta was quantified. Scale bar: 20 μm (n = 3 independent experimental repeats (at least 40 cells analysed from at least four fields of view per repeat)).

E Cells as in (C) were incubated with cell-permeable live/dead dye, imaged and cell death quantified. Scale bar: 100 μm (n = 3 independent experimental repeats (at least 300 cells analysed from at least six fields of view per repeat)).

F Senescent fibroblasts were incubated with LloMe and incubated with cell-permeable live/dead dyes. Scale bar: 100 μm (n = 2 independent experimental repeats (at least 200 cells analysed from at least four fields of view per repeat)).

Data information: All graphs show individual data points, mean and error bars represent standard deviation. All data (where n = 3) are analysed by 2-tailed, nonpaired Student's t-test (***< 0.001) except (E) which was analysed by one-way ANOVA with Tukey's multiple comparison test (***< 0.001).

 © 2023 The Authors

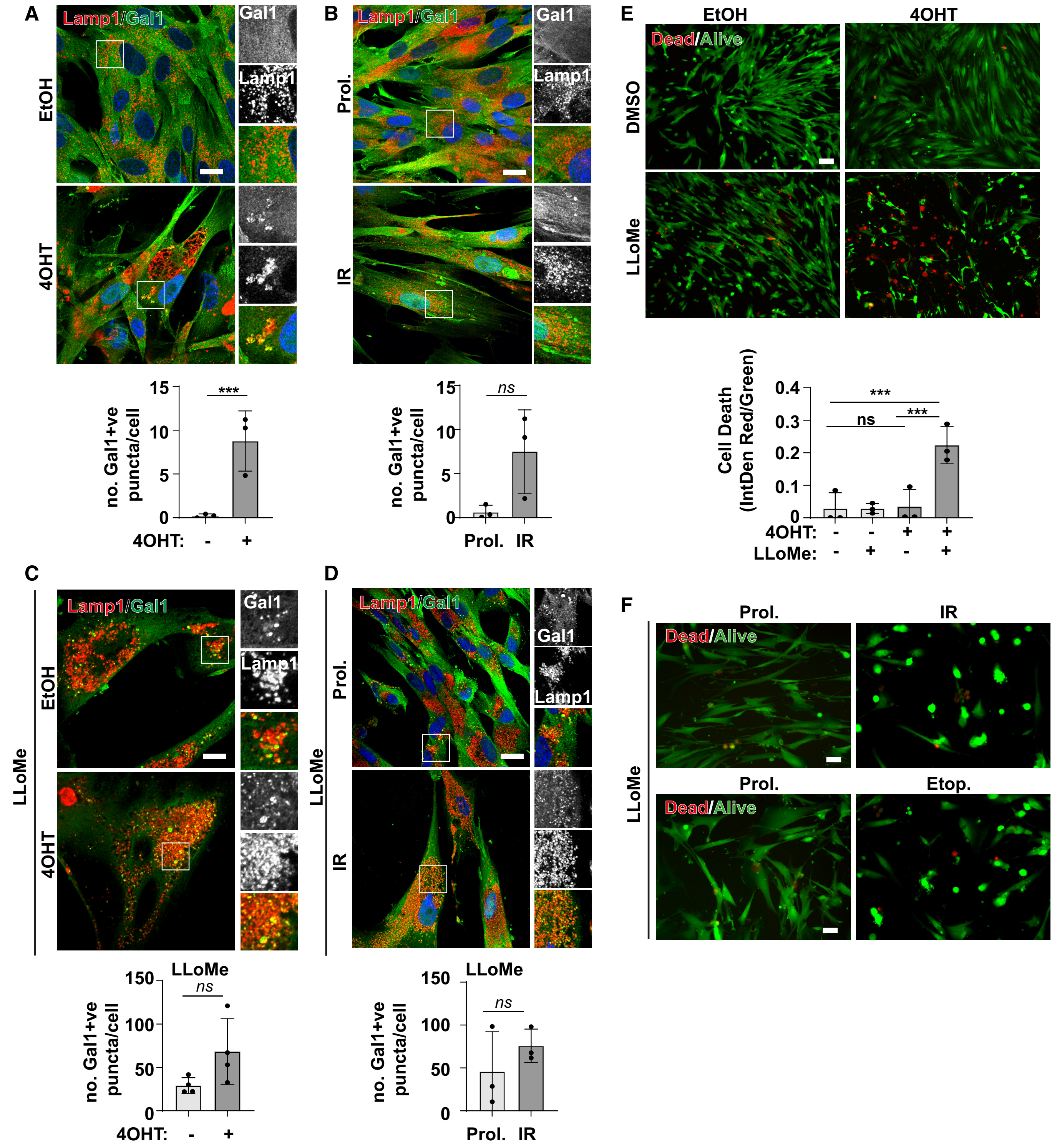
A
Lamp1/Gal1
Gal1
Lamp1
EtOH
4OHT
15
10
5
0
no. Gal1+ve puncta/cell
***
4OHT: - +
B
Lamp1/Gal1
Gal1
Lamp1
Prol.
IR
15
10
5
0
no. Gal1+ve puncta/cell
ns
Prol. IR
E
EtOH
4OHT
Dead/Alive
DMSO
LLoMe
Cell Death (IntDen Red/Green)
0.4
0.3
0.2
0.1
0
***
ns
***
4OHT: - - + +
LLoMe: - + - +
C
Lamp1/Gal1
Gal1
Lamp1
EtOH
4OHT
LLoMe
150
100
50
0
no. Gal1+ve puncta/cell
ns
4OHT: - +
D
Lamp1/Gal1
Gal1
Lamp1
Prol.
IR
LLoMe
150
100
50
0
no. Gal1+ve puncta/cell
ns
Prol. IR
F
Prol.
IR
Dead/Alive
LLoMe
Prol.
Etop.
Dead/Alive

Figure EV2.

Figure EV3. TFEB/TFE3 activation is a new hallmark of senescence.

A Quantification of nuclear TFE3 in EtOH and 4OHT-treated fibroblasts ($n = 3$ independent experimental repeats).

B Representative images and quantification of nuclear TFE3 in proliferating and replicative senescent (RS) fibroblasts. Scale bar: 20 μm ($n = 3$ independent experimental repeats (at least 100 cells analysed from at least five fields of view per repeat)).

C Representative images and quantification of nuclear TFE3 in proliferating versus etoposide or doxorubicin-induced senescent fibroblasts. Scale bar: 20 μm ($n = 3$ independent experimental repeats (at least 100 cells analysed from at least five fields of view per repeat)).

D Representative images and quantification of nuclear TFE3 in proliferating versus CDK4i-induced senescent fibroblasts. Scale bar: 20 μm ($n = 3$ independent experimental repeats (at least 100 cells analysed from at least five fields of view per repeat)).

E Quantification of nuclear TFE3 in proliferating and IR-induced senescent fibroblasts ($n = 3$ independent experimental repeats (at least 100 cells analysed from at least five fields of view per repeat)).

F Proliferating and IR-induced senescent fibroblasts immunostained for endogenous TFE3 in the conditions indicated; starvation indicates serum starvation overnight and 1-h amino acid starvation; refeeding was with full nutrient medium (including FCS) for 2 h. Cells were incubated with leptomycin B (LMB) for 3 h and where indicated, wash out involved replacement of the media for a further 2 h. Scale bar: 20 μm.

G Quantification of (F); % cells with nuclear TFE3 was quantified ($n = 3$ independent experimental repeats (at least 150 cells analysed from at least five fields of view per repeat)).

Data information: Violin blots in (G) include all datapoints from $n = 3$, lines represent median and upper and lower quartiles; Analysis: one-way ANOVA with Tukey's multiple comparison test *$P < 0.05$; ***$P < 0.001$. All other panels; graphs show individual data points, mean and error bars represent standard deviation. Analysed by 2-tailed, unpaired t-test *$P < 0.05$; **$P < 0.01$; ***$P < 0.001$.

 © 2023 The Authors

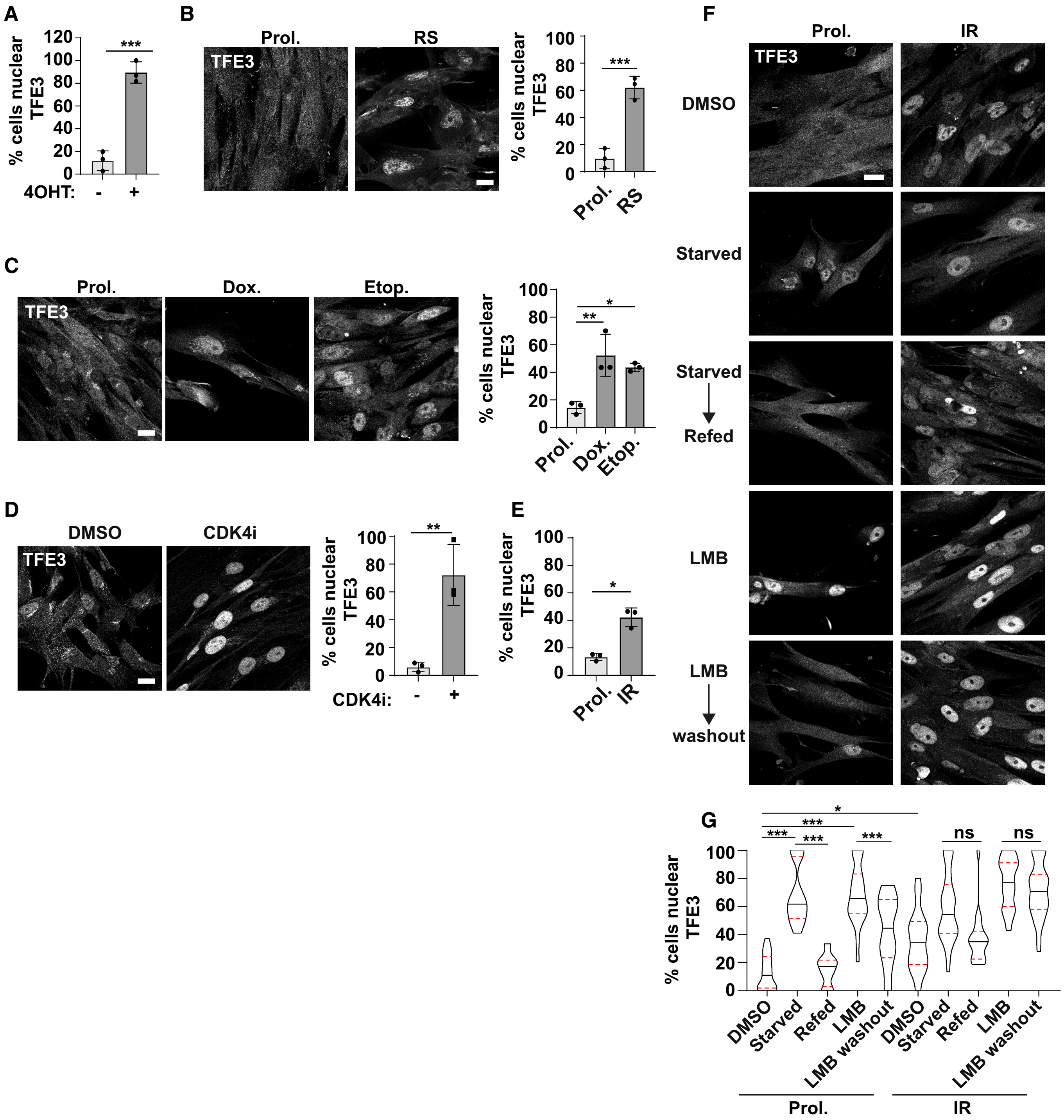

A
% cells nuclear TFE3
4OHT: - +
***
B
Prol.
RS
TFE3
% cells nuclear TFE3
Prol.
RS
***
C
Prol.
Dox.
Etop.
TFE3
% cells nuclear TFE3
**
*
D
DMSO
CDK4i
TFE3
% cells nuclear TFE3
CDK4i: - +
E
% cells nuclear TFE3
Prol.
IR
F
Prol.
IR
TFE3
DMSO
Starved
Starved → Refed
LMB
LMB → washout
G
% cells nuclear TFE3
DMSO
Starved
Refed
LMB
LMB washout
Prol.
IR
ns

Figure EV3.

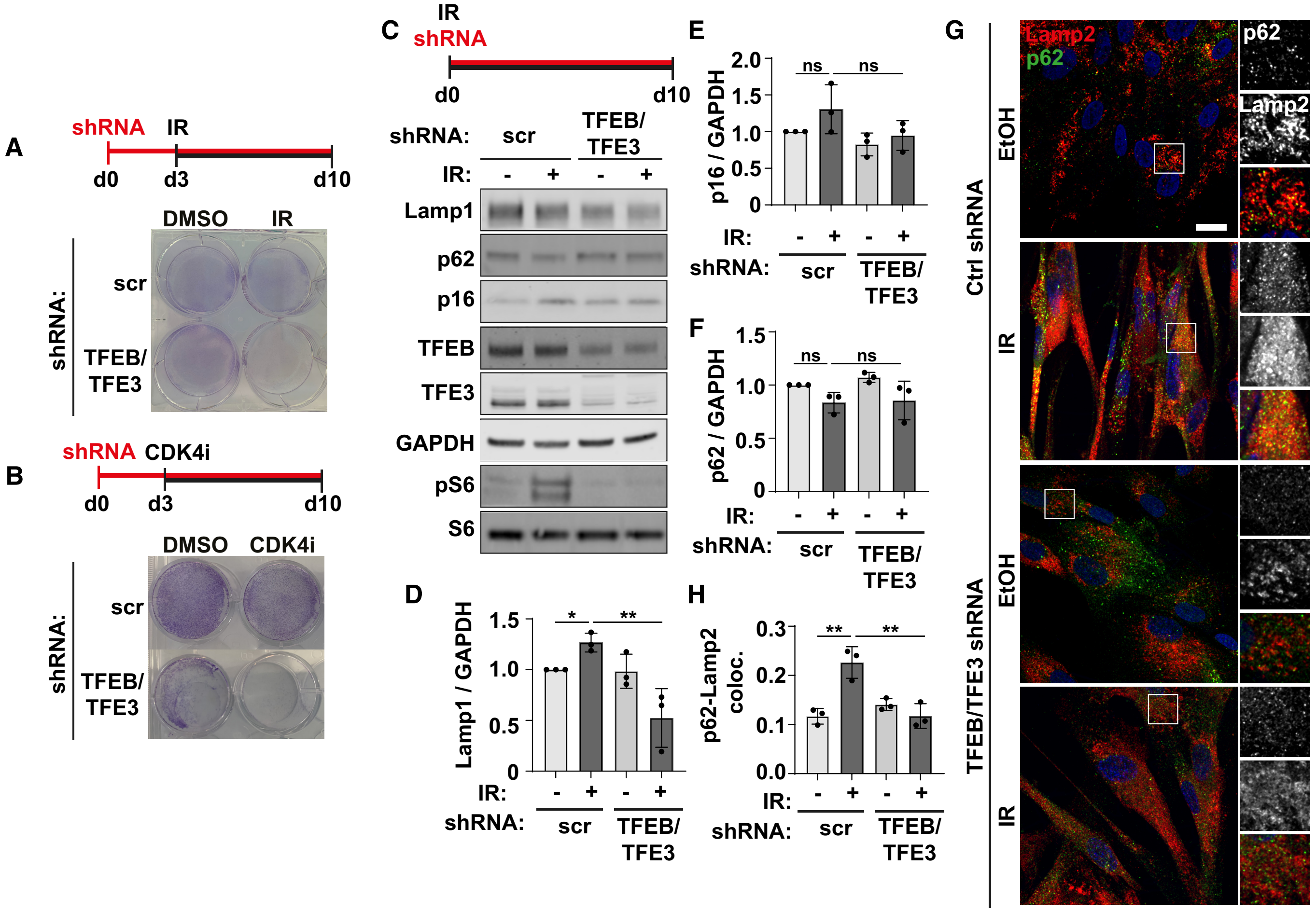

Figure EV4. TFEB/TFE3 drives lysosomal biogenesis in multiple models of senescence.

A Fibroblasts were transduced with TFEB and TFE3 shRNA before induction of senescence by IR. Cells were fixed and stained with crystal violet ($n = 1$).

B Fibroblasts were transduced with TFEB and TFE3 shRNA before induction of senescence by CDK4i. Cells were fixed and stained with crystal violet ($n = 1$).

C Fibroblasts were transduced with TFEB and TFE3 shRNA immediately following induction of senescence by IR (or incubated with proliferating controls) and analysed by Western blot 10 days later.

D–F Quantification of blots shown in (C) as indicated ($n = 3$ independent experimental repeats).

G Cells treated as in (C) were fixed and immunostained for Lamp2 and p62. Scale bar: 20 µm.

H Quantification of p62-Lamp2 colocalisation (Mander's coefficient) from (G) ($n = 3$ independent experimental repeats (at least 40 cells analysed from at least four fields of view per repeat)).

Data information: All graphs show individual data points, mean and error bars represent standard deviation. All data were analysed by one-way ANOVA with Tukey's multiple comparison test (*< 0.05, **< 0.01).
Source data are available online for this figure.

 © 2023 The Authors

Figure EV5. Defects in RagC-mTORC1 axis contribute to nuclear accumulation of TFEB/TFE3.

A Fibroblasts treated with EtOH or 4OHT were fixed and immunostained for Lamp2 and RagC. Scale bar: 20 μm.
B Quantification of (B) (Mander's coefficient between Lamp2 and RagC) (*n* = 2 independent repeats (at least 40 cells analysed from at least four fields of view per repeat)).
C Cells as in (A) were lysed and subject to Western blot.
D Quantification of (C); expression of RagA, RagB and RagC, normalised to tubulin and relative to EtOH control (*n* = 2 independent experimental repeats).
E Fibroblasts stably expressing GFP or GFP-RagC^{75L} were treated with EtOH or 4OHT as indicated, fixed and immunostained for endogenous TFE3. Scale bar: 20 μm.
F Quantification of (E) (*n* = 2 independent experimental repeats (at least 50 cells analysed in each condition from at least five fields of view per repeat)).
G Fibroblasts stably expressing GFP or GFP-RagC^{75L} and FLAG-TFEB were treated with EtOH or 4OHT as indicated. Cells were lysed and subject to Western blotting.
H Quantification of (G) (*n* = 4 independent experimental repeats).
I Quantification of (G) (*n* = 4 independent experimental repeats).
J Fibroblasts were transduced with p16 shRNA simultaneously with induction of senescence by 4OHT. Cells were subject to starvation (serum-free media overnight, 1 h amino acid-free media) or starved and refed (starvation protocol as above, refeeding with full nutrient media for 2 h). Cells were fixed and immunostained for endogenous TFE3. Scale bar: 100 μm.
K Quantification of (J) (*n* = 2 independent repeats (at least 100 cells analysed in each condition from at least five fields of view per repeat)).

Data information: All graphs show individual data points, and error bars represent standard deviation. Data analysed by one-way ANOVA with Tukey's multiple comparison test (***< 0.001).
Source data are available online for this figure.

© 2023 The Authors

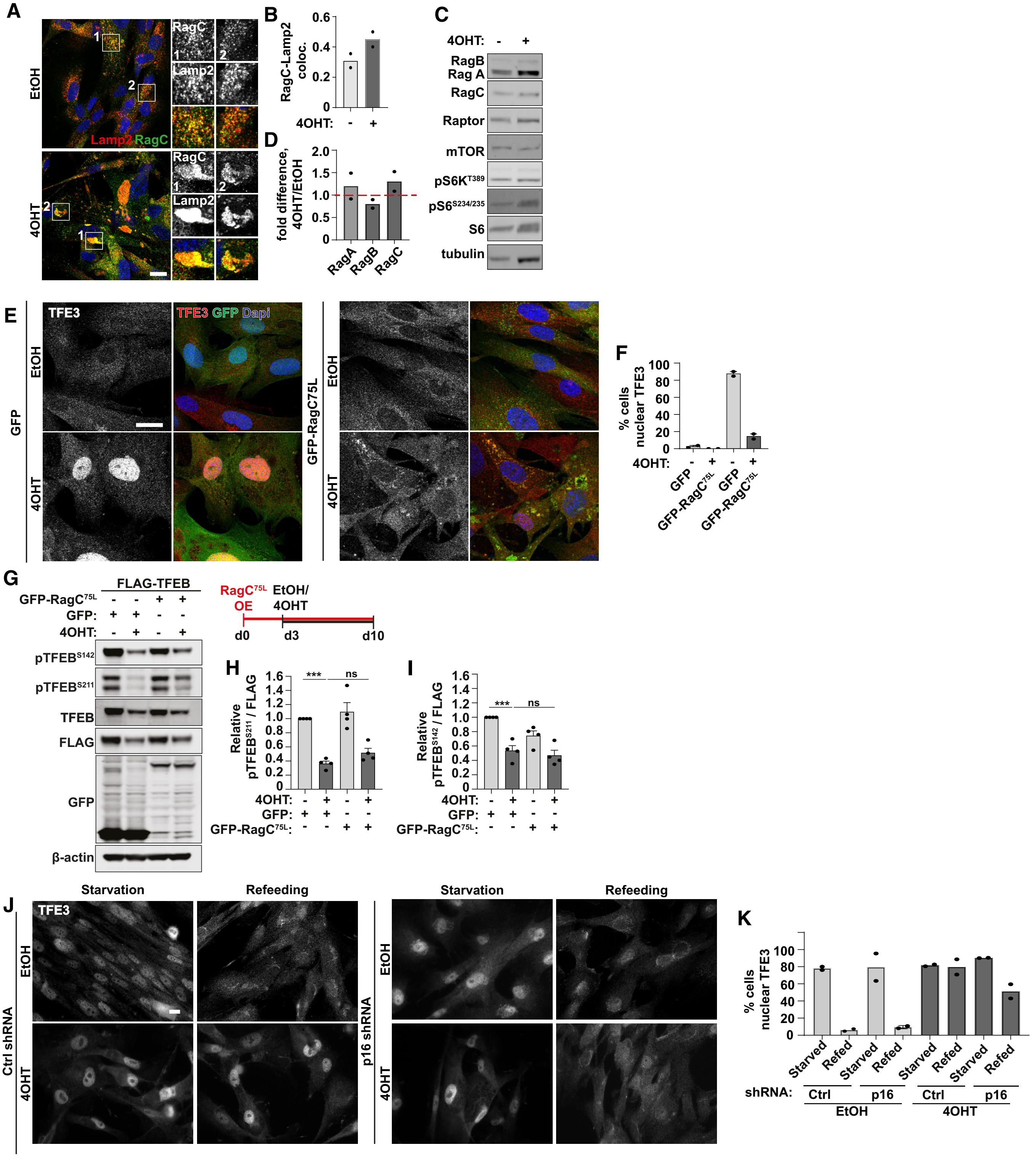

Figure EV5.

 © 2023 The Authors
