# Supplementary figures and images for: TFEB‐dependent lysosome biogenesis is required for senescence
Source: EMBO J. 2023 Mar 27;42(9):e111241. doi: 10.15252/embj.2022111241 (PMC10152146; doi:10.15252/embj.2022111241)

# Appendix S4

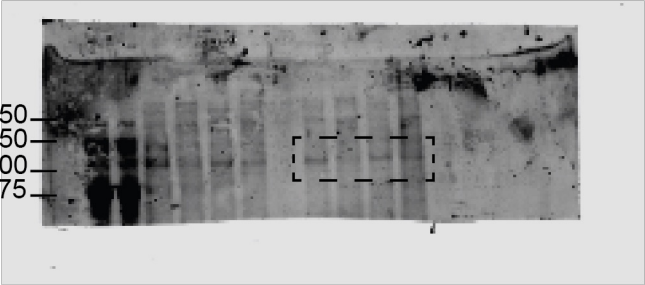

p-Rb<sup>S807/811</sup>

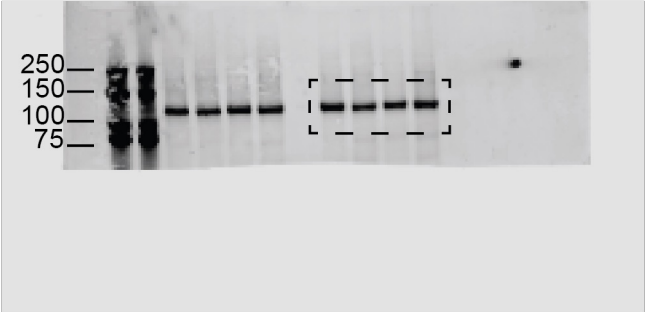

Rb

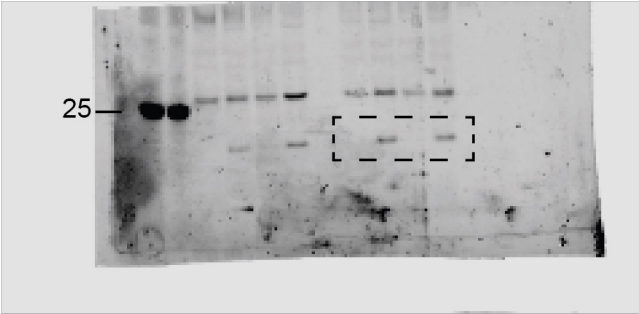

p16

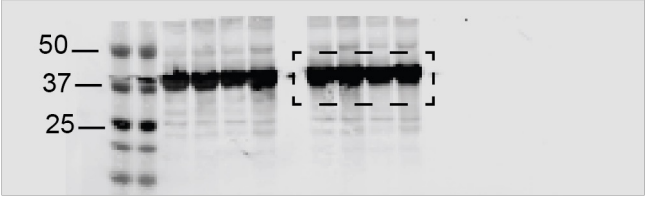

GAPDH

Supplement: Supplementary file 4 — Source Data for Expanded View [file EMBJ-42-e111241-s004.zip › Source data PDFs/Appendix S5.pdf]

# Figure EV5

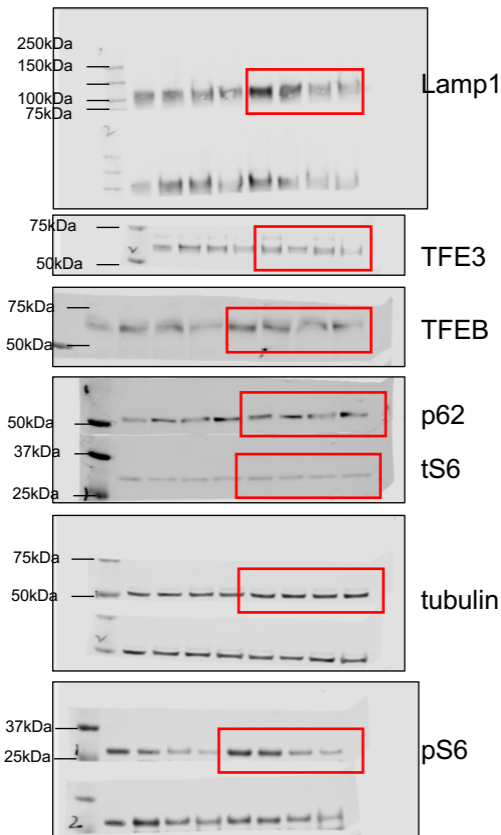

Supplement: Supplementary file 4 — Source Data for Expanded View [file EMBJ-42-e111241-s004.zip › Source data PDFs/Appendix S4.pdf]

# Appendix S1A

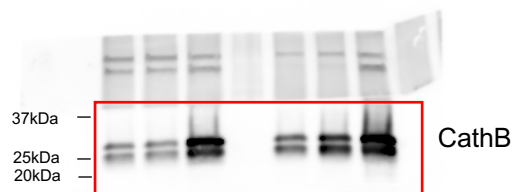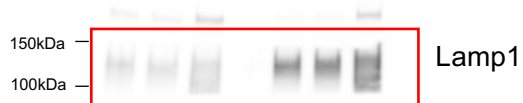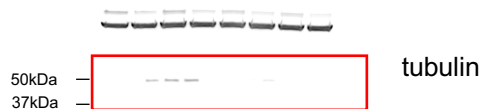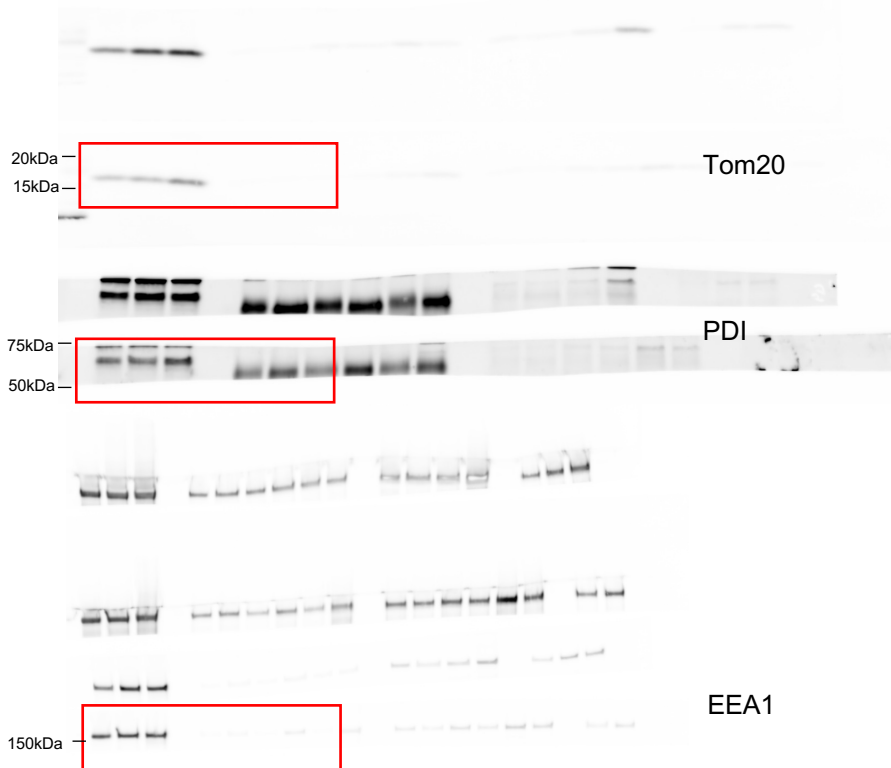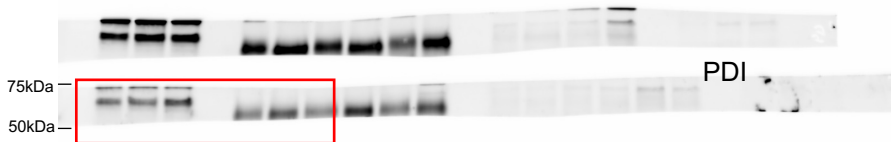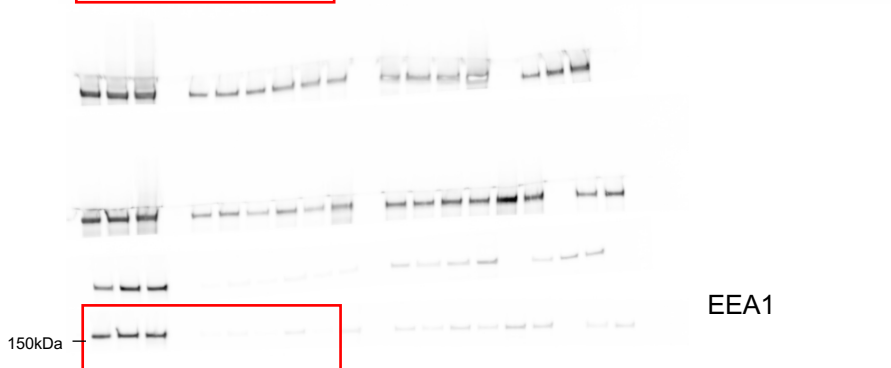

Supplement: Supplementary file 4 — Source Data for Expanded View [file EMBJ-42-e111241-s004.zip › Source data PDFs/Appendix S1.pdf]

# Appendix S3

## S3A

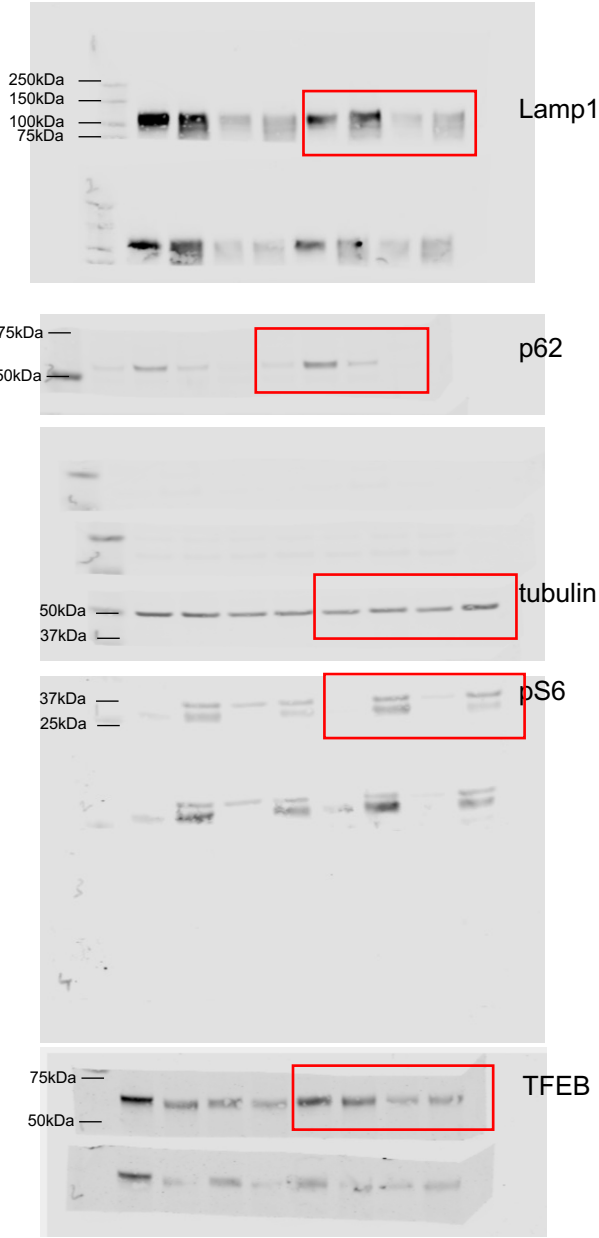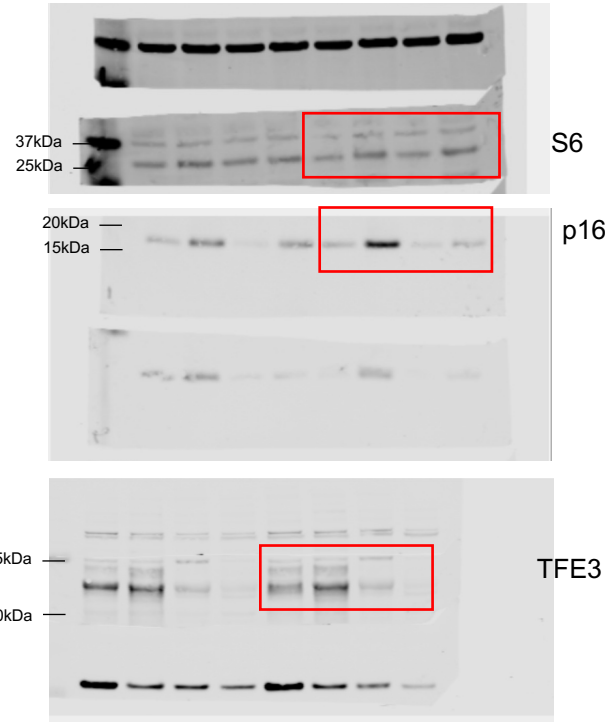

## S3G

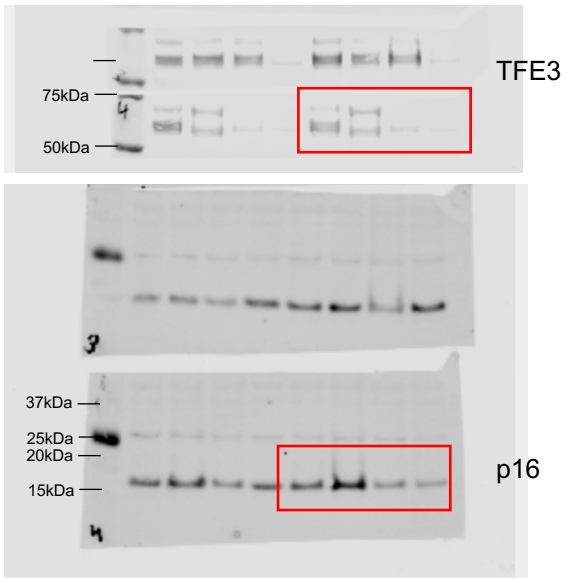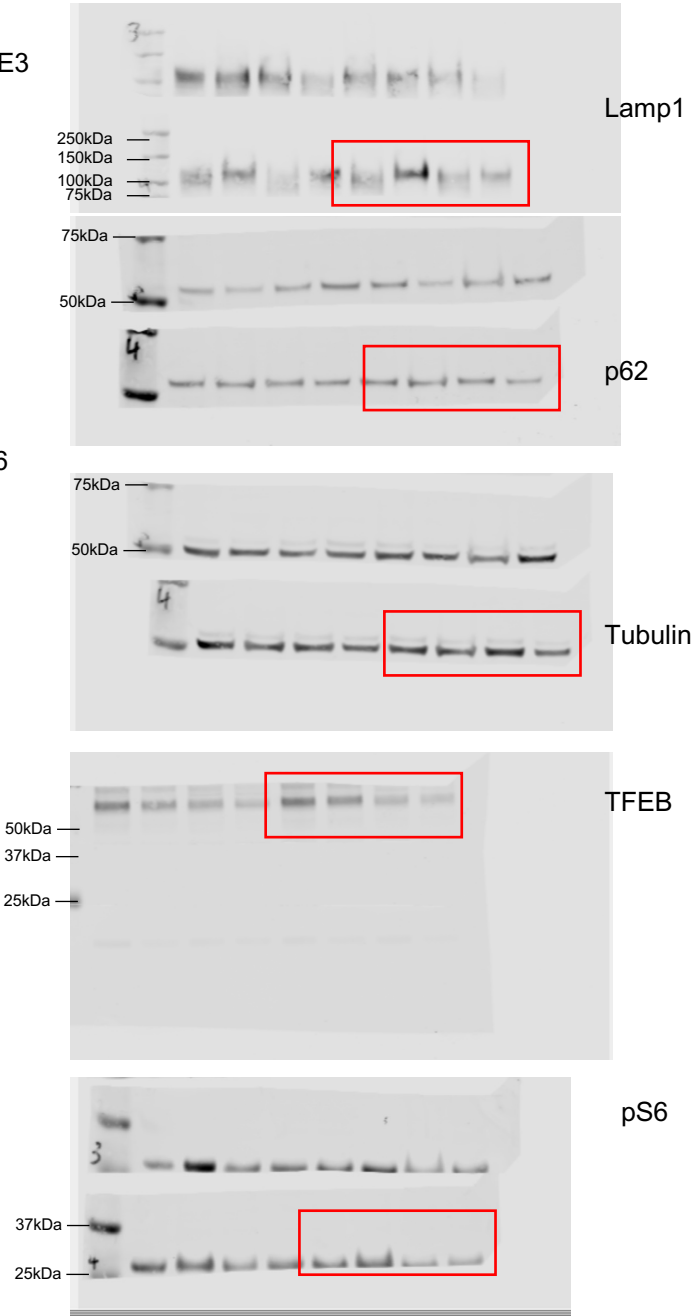

Supplement: Supplementary file 4 — Source Data for Expanded View [file EMBJ-42-e111241-s004.zip › Source data PDFs/Appendix S3.pdf]

# Appendix S2

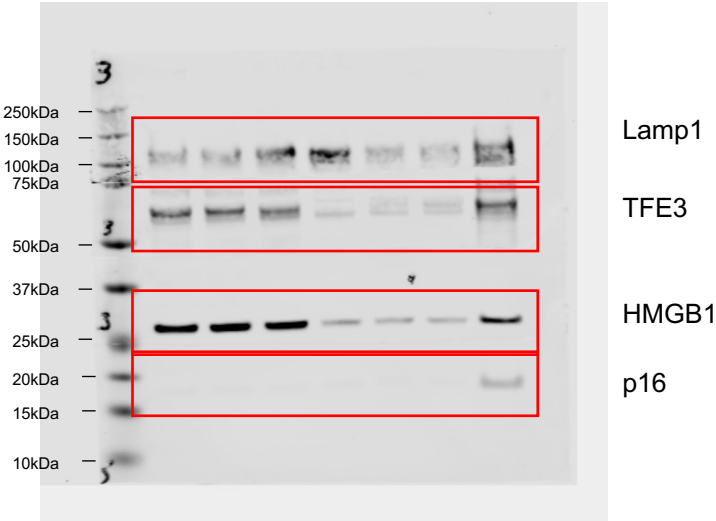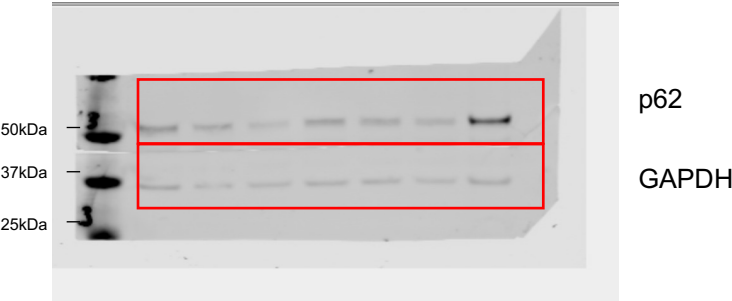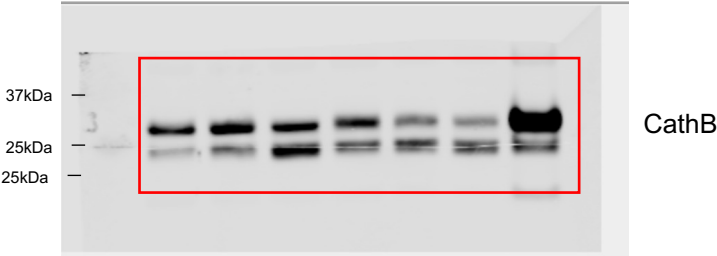

Supplement: Supplementary file 4 — Source Data for Expanded View [file EMBJ-42-e111241-s004.zip › Source data PDFs/Appendix S2.pdf]

# Figure EV4

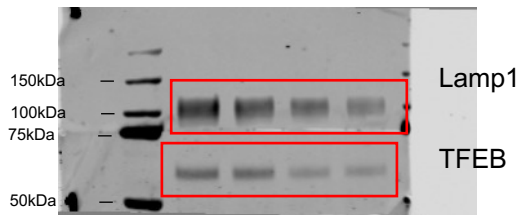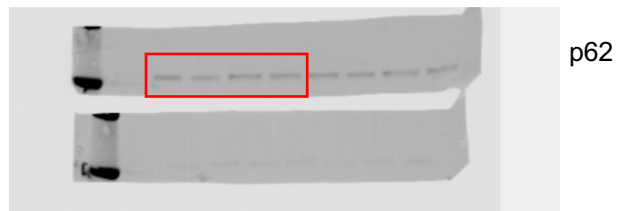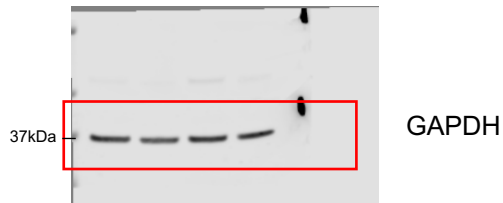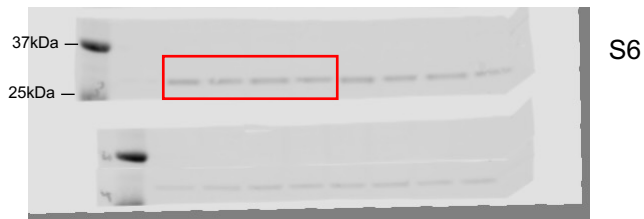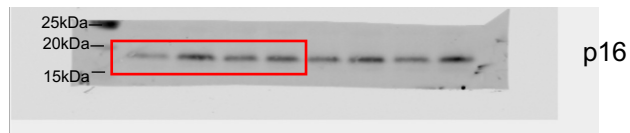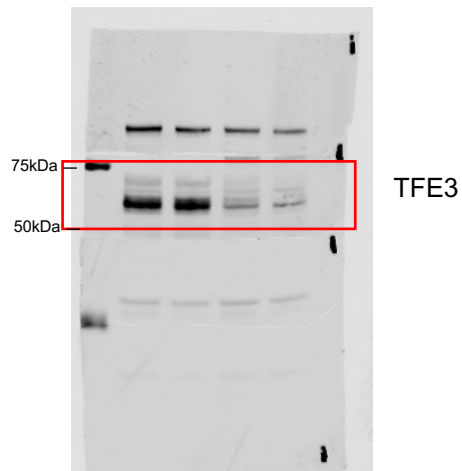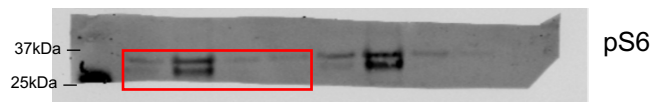

Supplement: Supplementary file 4 — Source Data for Expanded View [file EMBJ-42-e111241-s004.zip › Source data PDFs/Figure EV4.pdf]

# Figure EV6

Fig.EV6C

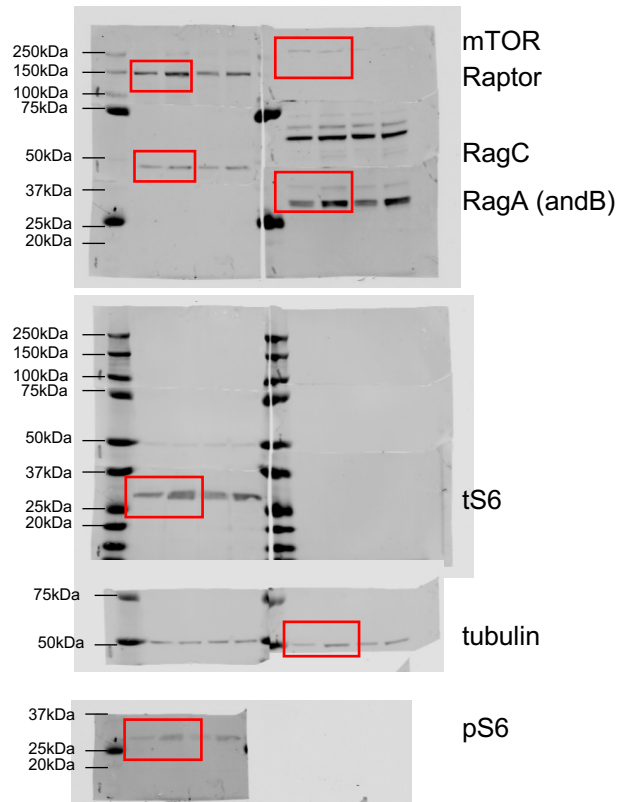

Fig.EV6G

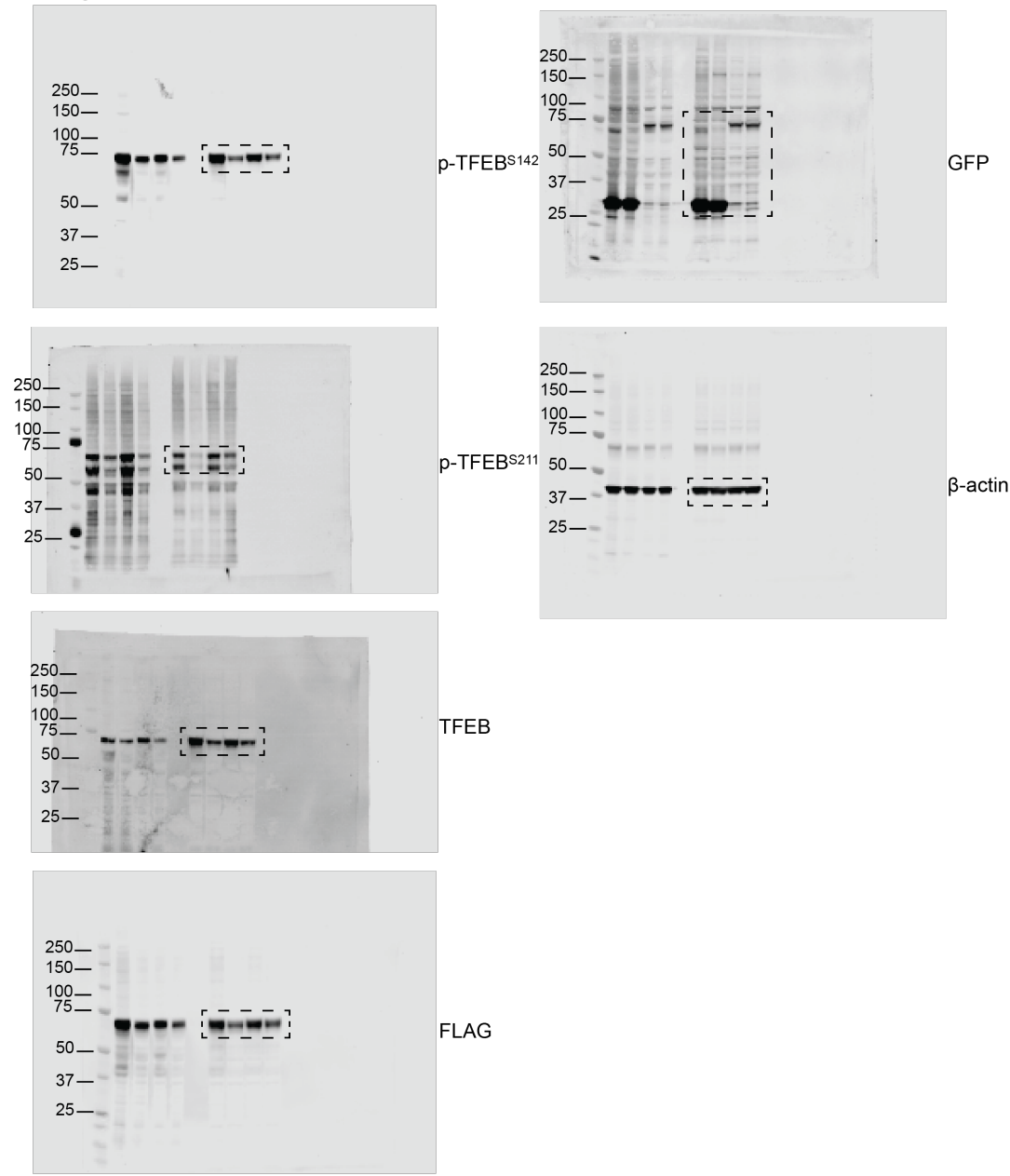

Supplement: Supplementary file 4 — Source Data for Expanded View [file EMBJ-42-e111241-s004.zip › Source data PDFs/Figure EV5.pdf]

Figure EV1

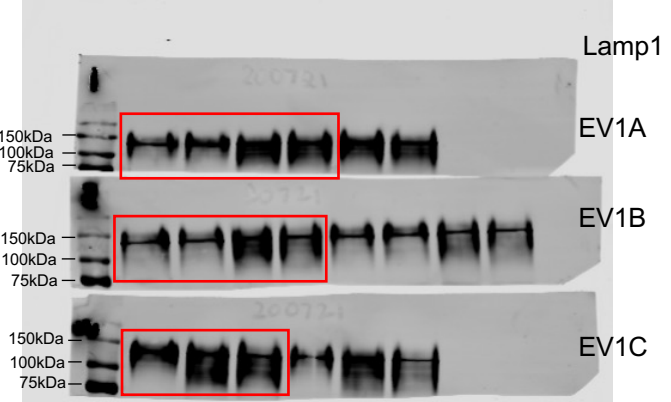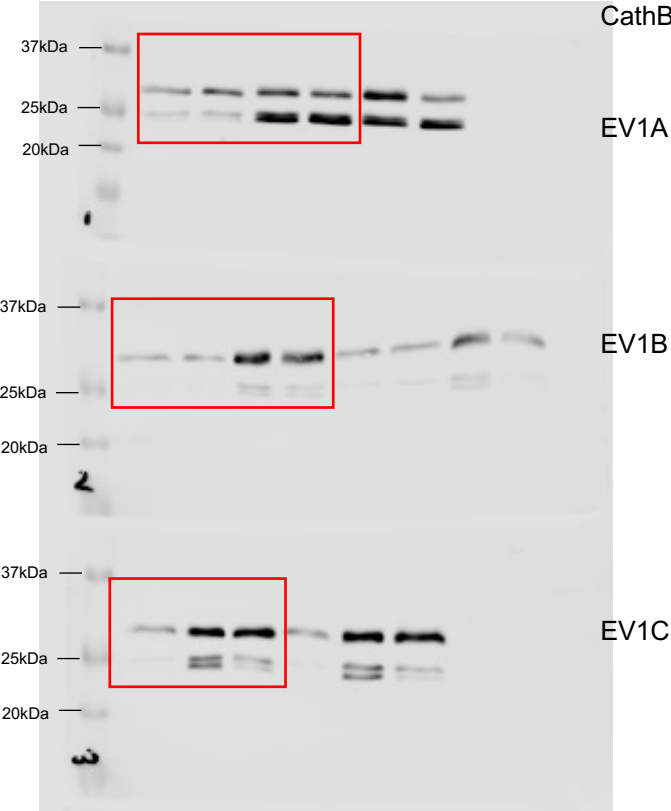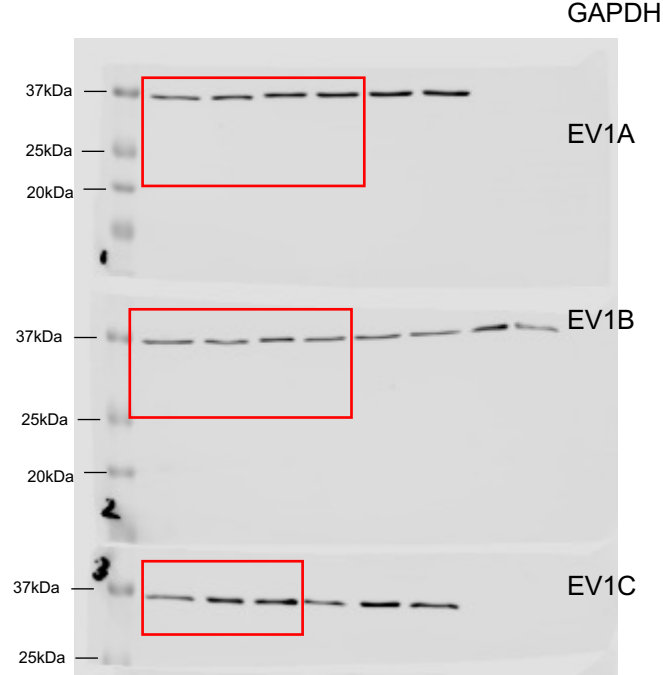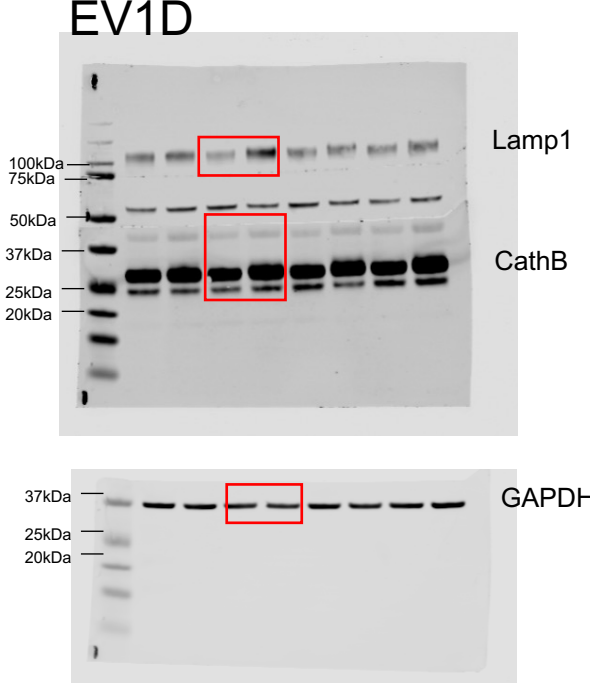

Supplement: Supplementary file 4 — Source Data for Expanded View [file EMBJ-42-e111241-s004.zip › Source data PDFs/Figure EV1.pdf]

Figure 1

Fig.1B

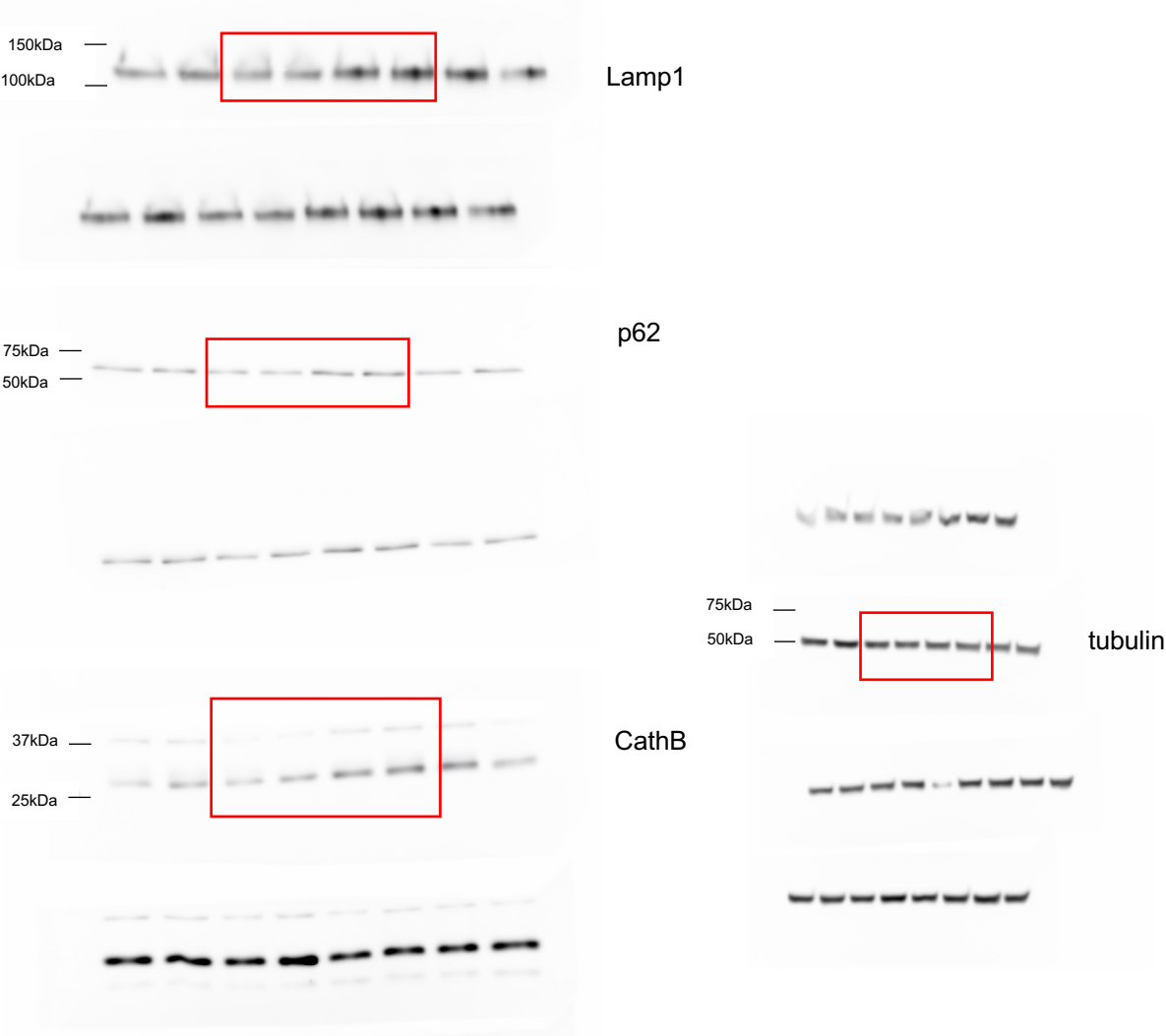

Fig.1O

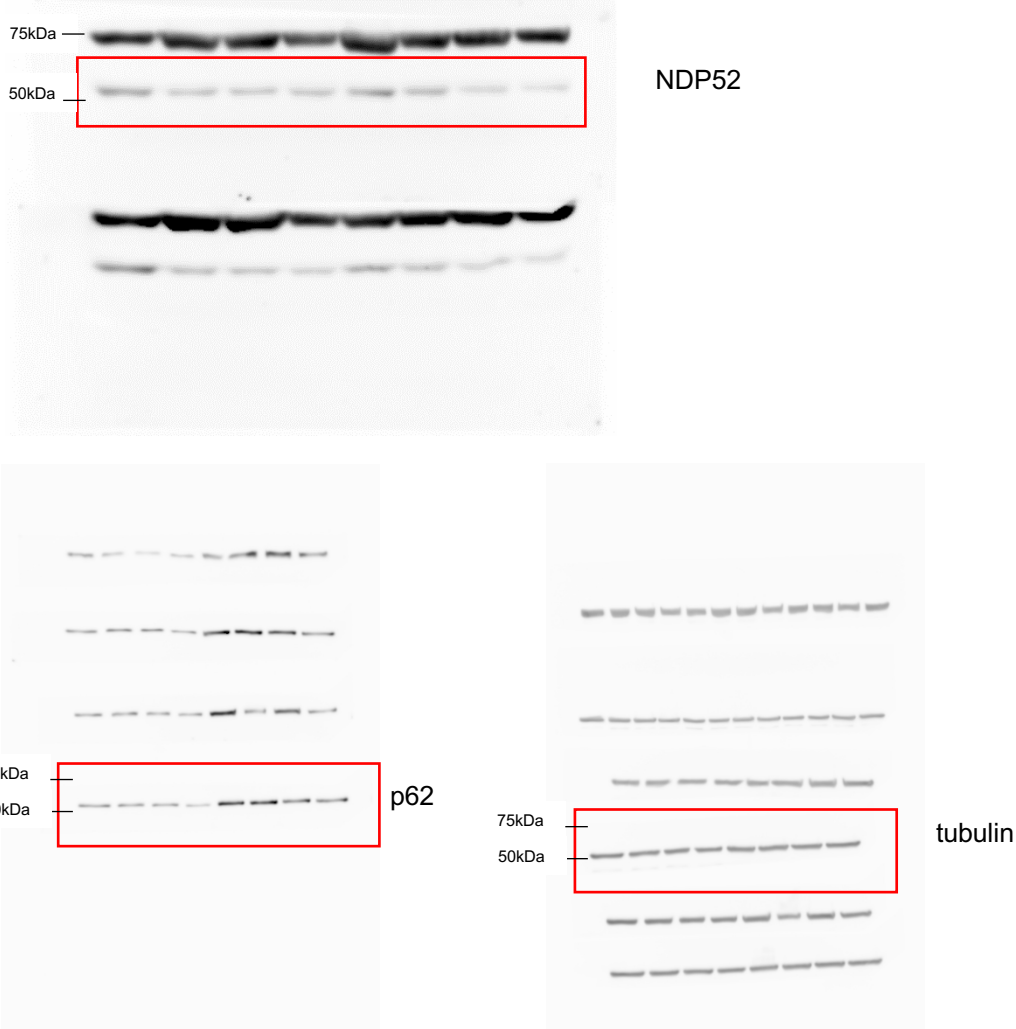

Supplement: Supplementary file 6 — Source Data for Figure 1 [file EMBJ-42-e111241-s007.zip › Figure 1.pdf]

# Figure 3

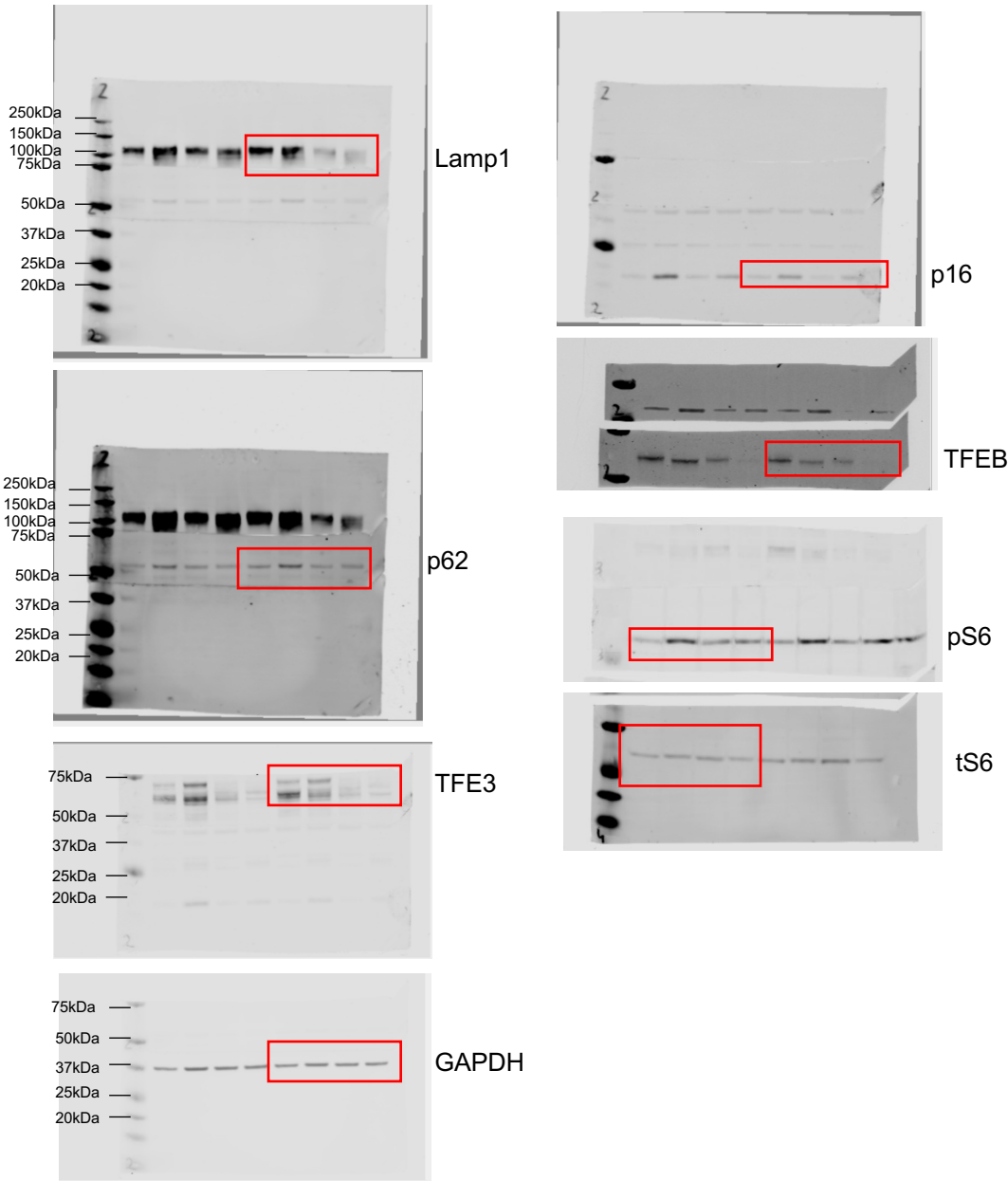

Supplement: Supplementary file 7 — Source Data for Figure 3 [file EMBJ-42-e111241-s003.zip › Figure 3.pdf]

Figure 4

Fig.4A

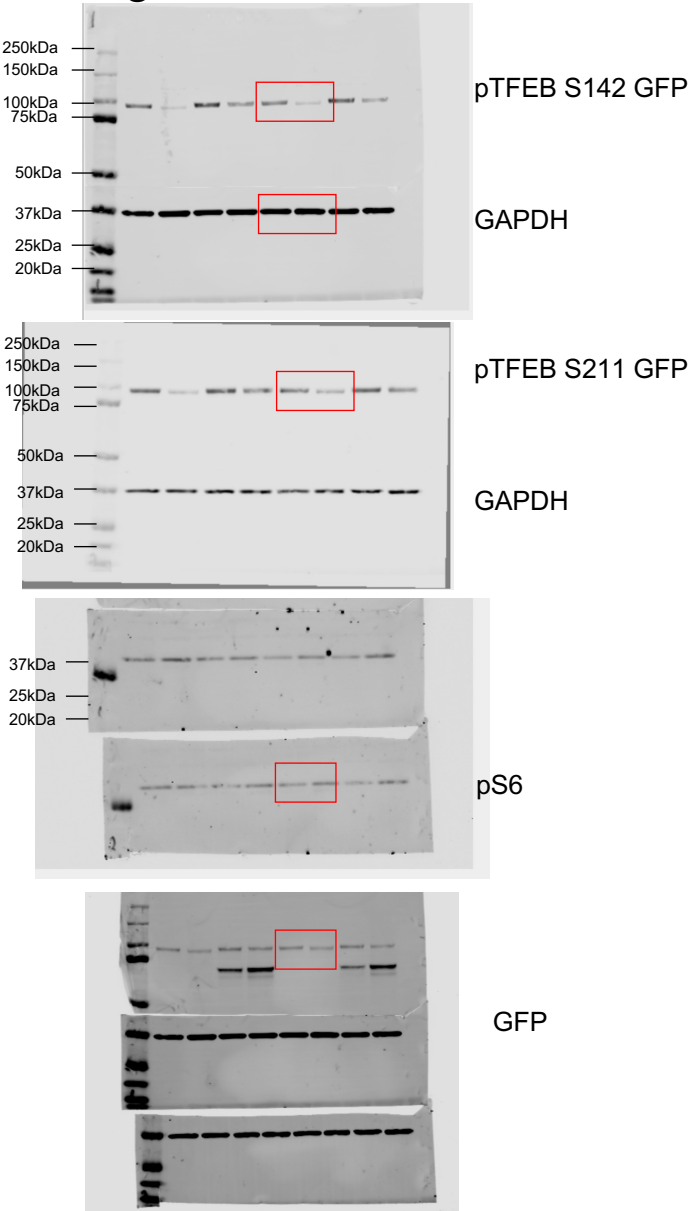

Figure 4 D

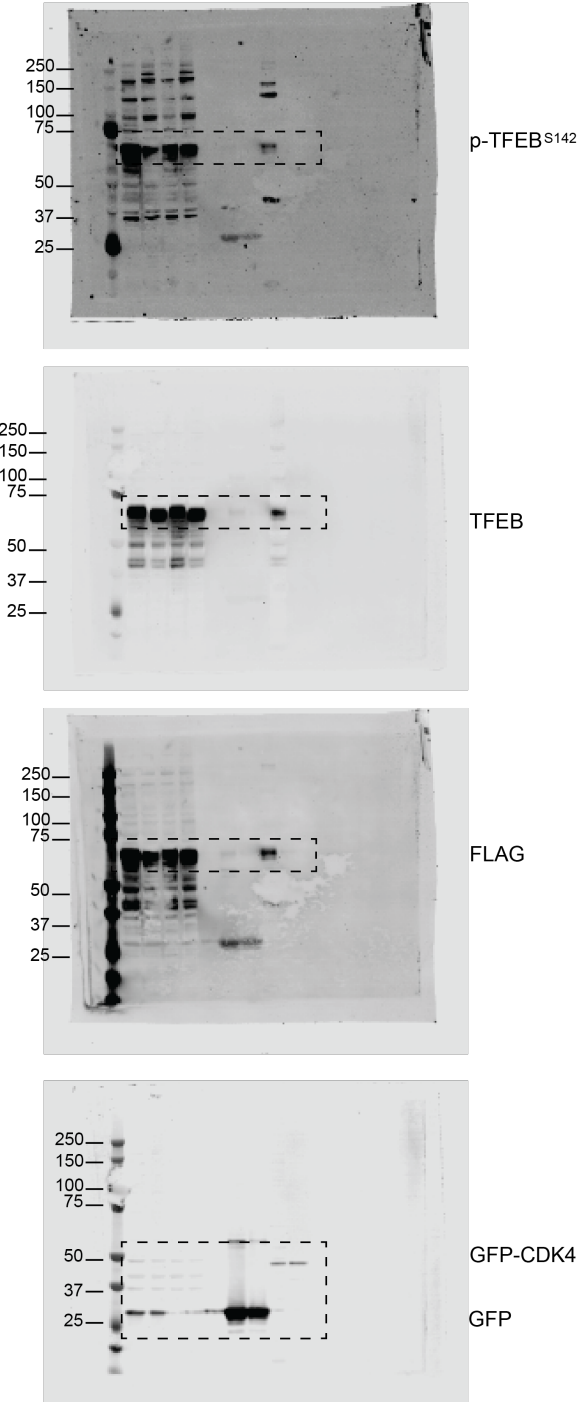

Fig.4H

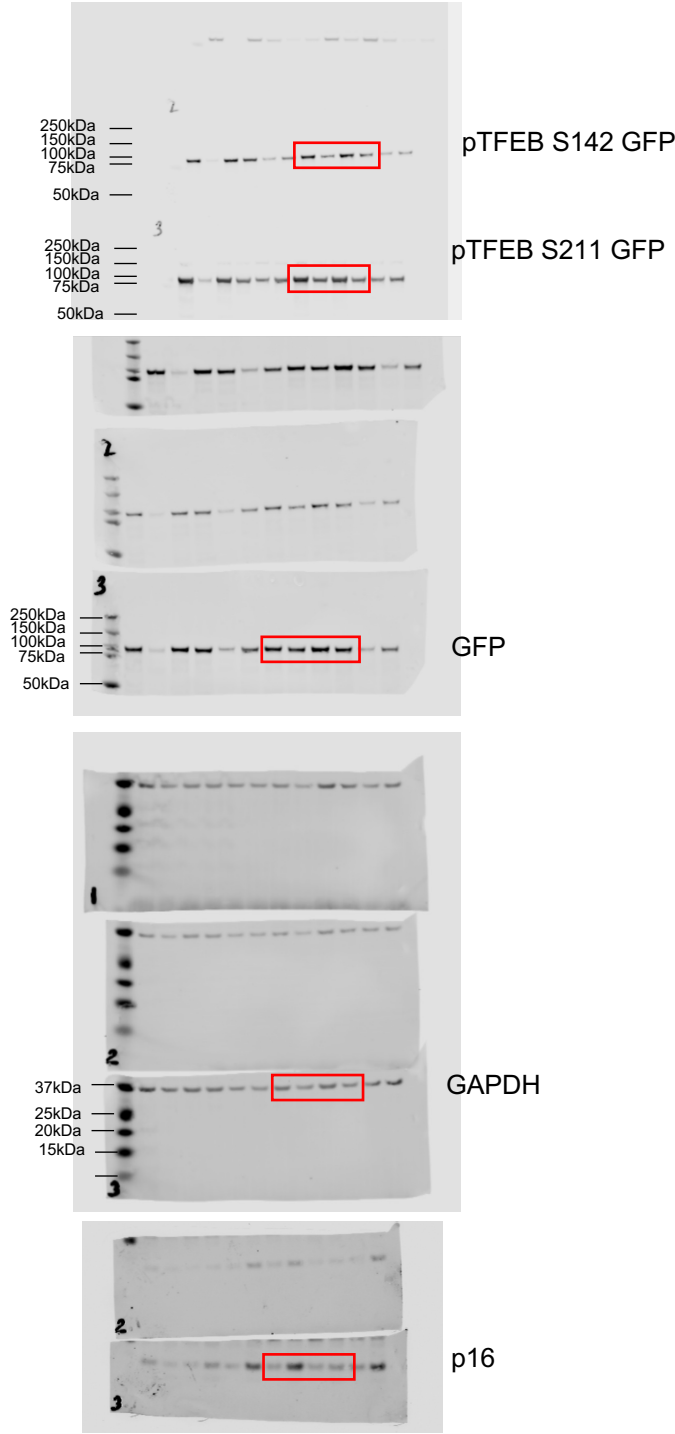

Fig.4J

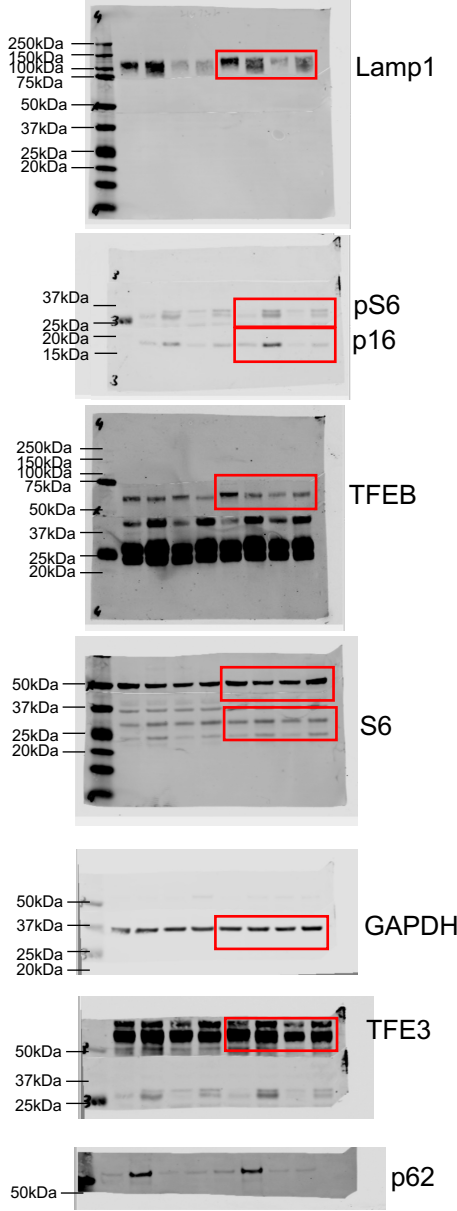

Supplement: Supplementary file 8 — Source Data for Figure 4 [file EMBJ-42-e111241-s002.zip › Figure 4.pdf]
