# Supplementary material for: TFEB‐dependent lysosome biogenesis is required for senescence
Source: EMBO J. 2023 Mar 27;42(9):e111241. doi: 10.15252/embj.2022111241 (PMC10152146; doi:10.15252/embj.2022111241)
Supplement: Supplementary file 9 [file EMBJ-42-e111241-s006.pdf]

THE EMBO JOURNAL

TFEB-dependent lysosome biogenesis is required for senescence

Bernadette Carroll, Rachel Curnock, Katy Yalci, Johan Palmfeldt, Marja Jäättelä, and Bin Liu
DOI: 10.15252/embj.2022111241

Corresponding author(s): Bernadette Carroll (bernadette.carroll@bristol.ac.uk)

Review Timeline:

Submission Date:	22nd Mar 22
Editorial Decision:	21st Apr 22
Revision Received:	22nd Sep 22
Editorial Decision:	19th Oct 22
Revision Received:	26th Jan 23
Editorial Decision:	1st Feb 23
Revision Received:	15th Feb 23
Accepted:	21st Feb 23

Editor: William Teale

Transaction Report:

(Note: With the exception of the correction of typographical or spelling errors that could be a source of ambiguity, letters and reports are not edited. Depending on transfer agreements, referee reports obtained elsewhere may or may not be included in this compilation. Referee reports are anonymous unless the Referee chooses to sign their reports.)

1st Editorial Decision 21st Apr 2022

Dear Dr Carroll,

Thank you again for the submission of your manuscript entitled "TFEB-dependent lysosome biogenesis is required for senescence". I have now received the referees' reports, which are copied to the bottom of this message.

At its heart, all referees agree that the manuscript is timely and the topic is important. However, the feedback was not unambiguously positive. The data that you present will need to be given further support if they are to be published in EMBO Journal.

I would like to invite you to address the comments of all referees in a revised version of the manuscript. The referees ask for a substantial amount of work; our usual revision time of three months is used as a guideline, not a deadline (this may be especially relevant as reviewer 3 asks for a series of experiments to be performed on naturally aged cells); manuscripts frequently take longer to revise. I will be available and happy to talk next week if you have any questions, I recommend that we go over our next steps and discuss the referees' comments further over Zoom.

I should add that it is The EMBO Journal policy to allow only a single major round of revision and that it is therefore important to resolve these concerns at this stage. I believe the concerns of the referees are reasonable and addressable, but please contact me if you have any questions, need further input on the referee comments or if you anticipate any problems in addressing any of their points. Please, follow the instructions below when preparing your manuscript for resubmission.

I would also like to point out that as a matter of policy, competing manuscripts published during this period will not be taken into consideration in our assessment of the novelty presented by your study ("scooping" protection). We have extended this 'scooping protection policy' beyond the usual 3 month revision timeline to cover the period required for a full revision to address the essential experimental issues. Please contact me if you see a paper with related content published elsewhere to discuss the appropriate course of action.

When preparing your letter of response to the referees' comments, please bear in mind that this will form part of the Review Process File, and will therefore be available online to the community. For more details on our Transparent Editorial Process, please visit our website: https://www.embopress.org/page/journal/14602075/authorguide#transparentprocess

Again, please contact me at any time during revision if you need any help or have further questions.

Thank you very much again for the opportunity to consider your work for publication. I look forward to your revision.

Best regards,

William Teale

William Teale, Ph.D.
Editor
The EMBO Journal

When submitting your revised manuscript, please carefully review the instructions below and include the following items:

1) a .docx formatted version of the manuscript text (including legends for main figures, EV figures and tables). Please make sure that the changes are highlighted to be clearly visible.

2) individual production quality figure files as .eps, .tif, .jpg (one file per figure).

3) a .docx formatted letter INCLUDING the reviewers' reports and your detailed point-by-point response to their comments. As part of the EMBO Press transparent editorial process, the point-by-point response is part of the Review Process File (RPF), which will be published alongside your paper.

4) a complete author checklist, which you can download from our author guidelines (https://wol-prod-cdn.literatumonline.com/pb-assets/embo-site/Author Checklist%20-%20EMBO%20J-1561436015657.xlsx). Please insert information in the checklist that is also reflected in the manuscript. The completed author checklist will also be part of the RPF.

5) Please note that all corresponding authors are required to supply an ORCID ID for their name upon submission of a revised manuscript.

6) We require a 'Data Availability' section after the Materials and Methods. Before submitting your revision, primary datasets produced in this study need to be deposited in an appropriate public database, and the accession numbers and database listed under 'Data Availability'. Please remember to provide a reviewer password if the datasets are not yet public (see https://www.embopress.org/page/journal/14602075/authorguide#datadeposition). If no data deposition in external databases is needed for this paper, please then state in this section: This study includes no data deposited in external repositories. Note that the Data Availability Section is restricted to new primary data that are part of this study.

Note - All links should resolve to a page where the data can be accessed.

7) When assembling figures, please refer to our figure preparation guideline in order to ensure proper formatting and readability in print as well as on screen:
http://bit.ly/EMBOPressFigurePreparationGuideline

Please remember: Digital image enhancement is acceptable practice, as long as it accurately represents the original data and conforms to community standards. If a figure has been subjected to significant electronic manipulation, this must be noted in the figure legend or in the 'Materials and Methods' section. The editors reserve the right to request original versions of figures and the original images that were used to assemble the figure.

8) For data quantification: please specify the name of the statistical test used to generate error bars and P values, the number (n) of independent experiments (specify technical or biological replicates) underlying each data point and the test used to calculate p-values in each figure legend. The figure legends should contain a basic description of n, P and the test applied. Graphs must include a description of the bars and the error bars (s.d., s.e.m.).

9) We would also encourage you to include the source data for figure panels that show essential data. Numerical data can be provided as individual .xls or .csv files (including a tab describing the data). For 'blots' or microscopy, uncropped images should be submitted (using a zip archive or a single pdf per main figure if multiple images need to be supplied for one panel). Additional information on source data and instruction on how to label the files are available at .

10) We replaced Supplementary Information with Expanded View (EV) Figures and Tables that are collapsible/expandable online (see examples in https://www.embopress.org/doi/10.15252/embj.201695874). A maximum of 5 EV Figures can be typeset. EV Figures should be cited as 'Figure EV1, Figure EV2" etc. in the text and their respective legends should be included in the main text after the legends of regular figures.

- For the figures that you do NOT wish to display as Expanded View figures, they should be bundled together with their legends in a single PDF file called *Appendix*, which should start with a short Table of Content. Appendix figures should be referred to in the main text as: "Appendix Figure S1, Appendix Figure S2" etc. See detailed instructions regarding expanded view here: .

- Additional Tables/Datasets should be labelled and referred to as Table EV1, Dataset EV1, etc. Legends have to be provided in a separate tab in case of .xls files. Alternatively, the legend can be supplied as a separate text file (README) and zipped together with the Table/Dataset file.

11) Our journal encourages inclusion of *data citations in the reference list* to directly cite datasets that were re-used and obtained from public databases. Data citations in the article text are distinct from normal bibliographical citations and should directly link to the database records from which the data can be accessed. In the main text, data citations are formatted as follows: "Data ref: Smith et al, 2001" or "Data ref: NCBI Sequence Read Archive PRJNA342805, 2017". In the Reference list, data citations must be labeled with "[DATASET]". A data reference must provide the database name, accession number/identifiers and a resolvable link to the landing page from which the data can be accessed at the end of the reference. Further instructions are available at .

Further instructions for preparing your revised manuscript:

Please make sure you upload a letter of response to the referees' comments together with the revised manuscript.

Please also check that the title and abstract of the manuscript are brief, yet explicit, even to non-specialists.

When assembling figures, please refer to our figure preparation guideline in order to ensure proper formatting and readability in print as well as on screen:
https://bit.ly/EMBOPressFigurePreparationGuideline
See also guidelines for figure legends: https://www.embopress.org/page/journal/14602075/authorguide#figureformat

IMPORTANT: When you send the revision we will require

- a point-by-point response to the referees' comments, with a detailed description of the changes made (as a word file).
- a word file of the manuscript text.
- individual production quality figure files (one file per figure)
- a complete author checklist, which you can download from our author guidelines (https://www.embopress.org/page/journal/14602075/authorguide).
- Expanded View files (replacing Supplementary Information)
Please see out instructions to authors
https://www.embopress.org/page/journal/14602075/authorguide#expandedview

Please remember: Digital image enhancement is acceptable practice, as long as it accurately represents the original data and conforms to community standards. If a figure has been subjected to significant electronic manipulation, this must be noted in the figure legend or in the 'Materials and Methods' section. The editors reserve the right to request original versions of figures and the original images that were used to assemble the figure.

Further information is available in our Guide For Authors: https://www.embopress.org/page/journal/14602075/authorguide

We realize that it is difficult to revise to a specific deadline. In the interest of protecting the conceptual advance provided by the work, we recommend a revision within 3 months (11th Jul 2022). Please discuss the revision progress ahead of this time with the editor if you require more time to complete the revisions. Use the link below to submit your revision:

https://emboj.msubmit.net/cgi-bin/main.plex

Further instructions for preparing your revised manuscript:

Please make sure you upload a letter of response to the referees' comments together with the revised manuscript.

Please also check that the title and abstract of the manuscript are brief, yet explicit, even to non-specialists.

When assembling figures, please refer to our figure preparation guideline in order to ensure proper formatting and readability in print as well as on screen:
https://bit.ly/EMBOPressFigurePreparationGuideline
See also guidelines for figure legends: https://www.embopress.org/page/journal/14602075/authorguide#figureformat

IMPORTANT: When you send the revision we will require
- a point-by-point response to the referees' comments, with a detailed description of the changes made (as a word file).
- a word file of the manuscript text.
- individual production quality figure files (one file per figure)
- a complete author checklist, which you can download from our author guidelines (https://www.embopress.org/page/journal/14602075/authorguide).
- Expanded View files (replacing Supplementary Information)
Please see out instructions to authors
https://www.embopress.org/page/journal/14602075/authorguide#expandedview

Please remember: Digital image enhancement is acceptable practice, as long as it accurately represents the original data and conforms to community standards. If a figure has been subjected to significant electronic manipulation, this must be noted in the figure legend or in the 'Materials and Methods' section. The editors reserve the right to request original versions of figures and the original images that were used to assemble the figure.

Further information is available in our Guide For Authors: https://www.embopress.org/page/journal/14602075/authorguide

We realize that it is difficult to revise to a specific deadline. In the interest of protecting the conceptual advance provided by the work, we recommend a revision within 3 months (20th Jul 2022). Please discuss the revision progress ahead of this time with the editor if you require more time to complete the revisions. Use the link below to submit your revision:

https://emboj.msubmit.net/cgi-bin/main.plex

Referee #1:

In the present manuscript, Curnock et al explore the role of TFEB/TFE3-dependent lysosomal biogenesis in senescence. The authors show that induction of senescence promotes impairment of lysosomal function. This results in the activation of TFEB and TFE3 transcription factors and subsequent induction of lysosomal-autophagy transcriptional programs, thus allowing senescent cells to maintain degradative capacity. Mechanistically, the authors propose that p16/INK4a-mediated inhibition of CDK4 is responsible for TFEB/TFE3 activation in senescent cells.
Although the manuscript is interesting in principle, there are several conceptual and technical concerns that need to be addressed. First, the functional consequence of lysosomal inhibition and TFEB/TFE3 activation during senescence is unclear. In addition, although the authors propose that the activation of TFEB and TFE3 is required senescence induction, they also show that depletion of TFEB/TFE3 per se is able to induce senescence. These results seem to be contradictory. Finally, how mechanistically TFEB and TFE3 are activated during senescence has not been properly addressed and requires further elucidation.
Specific points:

1. As mentioned above, the functional consequence of lysosomal inhibition and TFEB/TFE3 activation during senescence is unclear. Although the authors propose that activation of TFEB and TFE3 is required for induction of senescence, their own data suggest instead that depletion of TFEB/TFE3 per se is sufficient to induce senescence (Figures 3 and S3). How do the authors explain these contradictory results? And what is the relevance of TFEB/TFE3 activation during senescence?

2. The mechanism by which TFEB/TFE3 are activated during senescence is unclear. The data shown in Figure 4 (i.e. S142 phosphorylation, CDK4-TFEB interaction, p16 silencing), to support a role for p16/INK4a and CDK4 in TFEB/TFE3 activation, are weak and not convincing. Overall, from the data shown no conclusions can be drawn on the role of CDK4 in the activation of TFEB and TFE3 during senescence. As the authors pointed out, recent evidence suggests that TFEB and TFE3 are controlled by mTORC1 through a substrate-specific mechanism that relies on the activity of RagC/D GTPases (PMID: 32612235, 34253722, 35358174). Importantly, this mechanism is specifically elicited by stimuli that converge on the lysosome, such as lysosomal stress or lysosomal damage (PMID: 32989250, 34597140). Thus, it is surprising that the authors did not attempt to determine whether this pathway plays a role in TFEB activation during senescence. The authors should test whether the expression of constitutively active RagC rescues TFEB/TFE3 localization in senescent cells. Furthermore, they should also verify if the phosphorylation of TFEB S211, a specific mTORC1 phosphorylation site, is affected during senescence, and whether this phosphorylation can be rescued by the expression of active RagC.

3. Tamoxifen, which has been used by the authors to induce the expression of HRasV12 in the oncogene-induced senescence model, is a well-known activator TFEB (PMID: 34411438). Thus, the experiments using 4-OHT are misleading, as 4-OHT treatment per se is known to promote TFEB nuclear translocation even in control cells. The authors should avoid this treatment and use a doxycycline-inducible (or similar) system as a model of oncogene-induced senescence. The mechanistic data proposed in point 2, as well as the other experiments described in the manuscript, should be corroborated in this system and always tested in different models of senescence.

4. To claim that TFEB is transcriptionally active in senescent cells, the authors only analyzed 3 target genes (i.e. p62, Lamp1, CathB - Fig. 1a). Many more TFEB target genes should be analyzed to make such a claim. It is surprising that the authors performed RNA-seq analyses of senescent cells and never showed whether a global increase in lysosomal/autophagy genes is observed during senescence. The authors should either provide these data or explain why the activation of TFEB/TFE3 does not lead to a global increase of their transcriptional programs.

5. Quantifications or statistical analyses for most IF data are missing and should be provided upon analysis of numerous cells in different experiments.

6. Page 3: "...genes upregulated in senescence (Fig.1a) are well-known TFEB/TFE3-responsive genes with TFEB/TFE3-binding CLEAR (Co-ordinated Lysosomal Expression and Regulation) motifs". Here an important reference showing TFEB control of lysosomal genes is missing (PMID: 21617040)

7. Page 3: "...we observed that blocking the CRM1-dependent nuclear export of TFE3 (by treatment with leptomycin B (LMB)) lead to its accumulation over a two-hour time period in proliferating cells. Following the wash-out of LMB, TFE3 was released from the nucleus (Fig. 2b-c)". These observations had been already reported in two independent manuscripts (PMID: 30120233, 29992949). These two references should be cited in this context.

Referee #2:

Curnock et al. investigate mechanism and functional consequences of lysosomal biogenesis in senescent cells. By analyzing the proteome, catabolic activity and integrity of lysosomes, the authors provide evidence for lysosomal dysfunction in senescent cells. Investigating the increased lysosomal content of senescent cells in a range of senescence models, the authors demonstrate a striking nuclear accumulation of the transcription factors TFEB and TFE3, which are master regulators of lysosomal biogenesis. Knockdown of TFEB and TFE3 strongly impairs viability of senescent cells and decreases expression of

several senescence-associated proteins. Mechanistically, the authors suggest that TFEB and TFE3 accumulate in the nucleus of senescent cells due to impaired nuclear export through p16-mediated inhibition of CDK4, which normally promotes nuclear export of TFEB and TFE3.

Cellular senescence is closely associated with lysosomal biogenesis, but the underlying mechanisms have been insufficiently understood. The insights of the present study into dysregulated activity of TFEB and TFE3 is thus potentially an important advance in the field of cellular senescence, and which could also be highly relevant for organismal aging and development of senolytic drugs. Nuclear accumulation of TFEB and TFE3 is convincingly demonstrated, but several other conclusions are limited by technical caveats and missing controls. In particular, the mechanistic link between p16, CDK4 and TFEB/TFE3 activity is insufficiently developed. Therefore, the conclusions of the present study are conceptually intriguing but rather speculative at this point, and require substantial additional evidence.

Major concerns:

1. Fig. 1d, e, Suppl. Fig. 1d: While the transcriptional upregulation of lysosomal genes in senescent cells has been well documented (doi: 10.1101/gad.519709), the authors' approach to characterize the lysosomal proteome of senescent cells could yield important novel insights. Unfortunately, the proteomics data are presented on a superficial level, which strongly limits their relevance. I am surprised that the authors only report GO terms, which are difficult to interpret and sometimes contradictory (e.g. various lysosomal hydrolase/catabolism-related GO-terms are both in the upregulated and downregulated categories). It is essential to display changes in selected lysosomal proteins in the figures and provide the whole proteomics dataset in the supplement. To allow the reader to assess the quality of lysosomal enrichment (the FedEx method does not really isolate lysosomes), it would also be very helpful to provide western blot data demonstrating the extent of enrichment of lysosomal proteins, as well as depletion of contamination markers (other organelles, cytosolic proteins).

2. Fig. 1f-k: The analysis of lysosomal catabolic activity is problematic. Bodipy-Pepstatin A is a fluorescent conjugate of the cathepsin D inhibitor pepstatin A, which binds to cathepsin D and thereby assays its abundance, but not its activity (doi: 10.1016/s0165-022x(00)00048-8)! To quantify lysosomal proteolytic activity, DQ BSA (and Bodipy-Pepstatin A) is normalized to total lysotracker intensity, which is determined by both lysosomal abundance and lysosomal pH and thus an ambiguous measure. To circumvent this caveat, the authors could normalize DQ BSA to LAMP1 or LAMP2. Alternatively, the authors could quantify DQ BSA by microscopy and normalize to lysotracker area, which would have the additional benefit to illustrate the distribution of proteolytic activity across the lysosomal population. Finally, considering the dramatic increase in cell size that accompanies senescence, I am not sure whether normalization to cell number is appropriate. Critical functions of lysosomes such as turnover of damaged organelles/macromolecules scale with a cell's volume, and normalization to cell size (or area) might thus biologically be a meaningful alternative (or additional) analysis.

3. The mechanistic part concerning the model that nuclear export of TFEB/TFE3 is suppressed via p16-mediated inhibition of CDK4 is interesting but rather preliminary and requires experimental substantiation. Does CDK4 inhibition promote lysosomal biogenesis in proliferating cells? Does p16 shRNA specifically reduce the nuclear pool of TFE3, or does this also affect the cytoplasmic pool? Does blocking nuclear export restore nuclear TFEB/TFE3 levels in p16-depleted cells? Do transcriptional targets of TFEB/TFE3 change at the level of mRNA expression upon the perturbation of CDK4 or p16?

4. Related to the previous point, the authors state that 'we show that mTORC1-dependent phosphorylation of TFEB at serine 142 is unaltered in senescence'. I assume that the authors mean that TFEB p-S142, which can be phosphorylated by mTORC1, is unaltered in senescence. This is critical, because S142 is also phosphorylated by CDK4, and at least in proliferating cell this is the critical phospho-site through which CDK4 promotes nuclear export of TFEB (DOI: 10.1083/jcb.201911036). Of note, the authors omit CDK4 from the discussion of possible TFEB S142 kinases. I understand that the phosphorylation of TFEB and TFE3 at multiple sites and by several kinases makes an analysis of the relevant phosphorylation sites challenging. However, the authors should at least investigate the effect of CDK4 or mTORC1 inhibition on TFEB p-S142 in proliferating and senescent cells and examine whether TFEB S142A is nuclear or cytoplasmic under these conditions. If S142 is not the relevant phosphosite, other residues that have been identified as CDK4 targets could be analyzed similarly.

5. The authors conclude that TFEB/TFE3-dependent lysosomal biogenesis is required for senescence acquisition and survival. Conceptually, these are two distinct processes and it would be interesting to investigate them separately. To examine the relevance of TFEB/TFE3 specifically for senescence acquisition, maybe the authors could genetically deplete TFEB/TFE3 first and subsequently induce senescence?

6. For the genetic loss-of-function experiments, the authors use a single shRNA for each manipulation. Given the well-documented broad off-target effects of shRNAs, one would expect to use at least two independent shRNAs for each gene and control. Also, please indicate the specific sequences that are used as scrambled controls.

Minor points:
1. Fig. 1: The authors state that 'lysosomal protease-related functional terms were downregulated in the senescent lysosomal preps'. However, based on the qPCR and western blotting data (Fig. 1a, b), CTSB is at least as much increased as LAMP1 in senescent cells. Proteomics and western blotting data for specific cathepsins would be helpful to resolve this apparent

contradiction (see also Major Point 1).

2. Fig. 2: The authors state that 'cytoplasmic-nuclear flux of TFE3 is inhibited in senescence'. Which data support this conclusion? This hypothesis is distinct from the authors' suggestion that a block in nuclear export of TFE3/TFEB leads to their nuclear accumulation.

3. Fig. 3b, g: The authors state that knockdown of TFE3/TFEB reduces the levels of LAMP1. This decrease is not obvious on the shown western blot, and the immunofluorescence data suggest that the related LAMP2 still is strongly upregulated. It would be helpful to demonstrate this decrease more convincingly, and if possible provide additional support with functional assays for lysosomal activity.

4. Fig. S3: The figure legend is titled 'TFEB/TFE3 is both necessary and sufficient for senescence'. Maybe I missed this, but which data demonstrate sufficiency for senescence induction?

5. Fig. S4a: The authors interpret the shift in electrophoretic mobility of TFEB as indicative for decreased phosphorylation. This could easily be confirmed experimentally by phosphatase treatment of the control sample.

6. Please include scale bars in all images.

7. The organization of figure panels is sometimes a bit confusing.

8. The figure legends are sometimes difficult to read, because several different experiments and techniques are described together.

9. The statistical analyses are sometimes difficult to interpret. Please indicate for each experiment whether n refers to technical or biological replicates. How many images were quantified? With how many cells?

Referee #3:

Summary:
The manuscript by Curnock et al. describes experimental evidence that several models of senescence alters gene and protein expression of lysosomal genes and associated functions. Moreover, they provide evidence that individual lysosomes might be impaired in their function (pH, degradation, damage), but this is offset by an upregulation in total lysosomes, which is proposed to mitigate the loss in lysosomal quality and maintain catabolic activity in senescing cells. They then go on to show that senescence triggers nuclear translocation of TFEB and TFE3, transcription factors known to induce expression of lysosomal, autophagy, and immune-protective genes. They propose that TFEB/TFE3 activation in senescent cells is the mechanisms that upregulates lysosomal levels and offsets lysosome quality loss. They show that silencing of TFEB/TFE3 sensitizes senescent cells to death, suggesting that TFEB/TFE3 activation is protective during senescence. They further propose that this occurs independently of mTORC1, a known inhibitory regulator of TFEB/TFE3, and instead occurs via p16/INK4a-dependent inhibition of CDK4, which associates with and was previously shown to modulate TFEB. This is an interest manuscript and has some tantalizing observations, but it suffers from a few problems that need to be addressed before publication. If the authors can address most of the issues below, I would support publication of their work in this journal.

Major
1. First, I'll start by criticizing their figure layout. It is messy and confusing. This needs rethinking and reorganization, please! Scale bars are missing.
2. The authors are inconsistent with their quantification of their data. Some blots are quantified, some are not (Example Fig. 1B, Supl Fig. 1). Some images are, some are not (example, Fig. 1C, 1J, 2G). In all cases, all data should be quantified and statistically analysed.

3. The authors need a section in the Methods that is dedicated to statistical analysis, which should state the assumptions made and the tests used, and why. In fact, the authors used only t-tests even when they have more than two conditions being compared without stating why! Unless there is a specific statistical reason, they should employ tests like ANOVA and post-hoc tests, depending on the assumptions. Then, some graphs have statistical significance indicated as * or its variations, while other graphs state p value like $p<0.3$, which I assume they are trying to argue for a trend. This needs to be more explicitly stated. Moreover, violin plots do not define was is being shown (mean, range, STD?). Some experiments were done $n=2$ (Fig. 3c) or $n=1$ is shown but stats test is done (Fig. 4g). Overall, their statistical analysis is a major weakness, but one that should and can be resolved. In some cases, the authors should consider increasing their experimental repeats to increase robustness.

4. Lysotracker Deep Red (LysoT) is used to normalize DQ-BSA and BODIPY-Pepstatin signal. However, the authors show that pH is affected and that LysoT per cell is affected by their treatments. So, unless, I'm missing something, I don't understand how Lysotracker can be used to normalize the proteolysis probes - any effect could be due to pH, not degradation. If DQ-BSA or

Pepstatin are not normalized, what is the value? This needs to be revisited. DQ-BSA could be co-endocytosed with a dye like dextran to normalize for uptake and trafficking to lysosomes. As shown, there may be other interpretations of their data other than that lysosome proteolysis is impaired by senescence.

5. The co-IP between TFEB-GFP and CDK4 is not convincing without quantification and indication of repeats in the legend. To boost the confidence in this result, the authors should reverse the co-IP by pulling down CDK4 and probing for TFEB-GFP.

6. The most significant issue in my opinion is as follows. All treatments used to cause senescence are complex and can result in other events that promote TFEB or lysosome "aging". While the authors are to be commended for trying many approaches to induce senescence, this falls short of aging-related senescence. I think it is not unreasonable to test if generational aging of cells like MRC-5 (30-40 generations) induces TFEB translocation, affects lysosome function, and lysosome levels. I am cognizant that this takes time, but the authors could maintain these cells over multiple generations as they address other issues and then test this. This is a clear flaw that reduces the impact of their work towards aging senescence rather than induced senescence.

Minor/Non-essential suggestions
1. Authors focus on lysosome number/amount, but their images indicate a significant impact on lysosome position (or this appears to be the case based on their images). Lysosome position can impact function - the authors should quantify images to determine if lysosome distribution is also an affected of treatments.

Please note, all Figure references in this document refer to the revised manuscript. Reviewer comments are in black text and the author responses are in red.

****

Referee #1:

1. ….The functional consequence of lysosomal inhibition and TFEB/TFE3 activation during senescence is unclear. Although the authors propose that activation of TFEB and TFE3 is required for induction of senescence, their own data suggest instead that depletion of TFEB/TFE3 per se is sufficient to induce senescence (Figures 3 and S3). How do the authors explain these contradictory results? And what is the relevance of TFEB/TFE3 activation during senescence?

The findings are consistent with the published role of autophagy in senescence induction and subsequent survival. Autophagy has been shown to be required for the initial mitotic transition to senescence (PMID: 19279323), while seemingly contradictory, long-term inhibition of autophagy actually promotes senescence and other premature ageing phenotypes *in vivo* (PMID: 31949142). Furthermore, we have shown previously that inhibition of autophagy sensitises senescent cells to death (PMID: 28566325). The autophagy-lysosome pathway has been demonstrated to be important in senescence for generating amino acids and supporting mTORC1 activity (PMID: 28566325; 21512002), which drives a number of senescence phenotypes. We have now added more context and description to the manuscript text regarding the role of lysosomes in senescence.

Furthermore, in light of the reviewer's comment, and without an extensive, in-depth investigation, it is premature for us to make any statements about TFEB/TFE3 loss and senescence. We have therefore toned down this conclusion in the text, and simply say there is evidence of increased stress markers in cell with loss of TFEB/TFE3. Given these data though, we would hypothesise that long-term reduction in TFEB/TFE3 activity *in vivo* would promote senescence and premature ageing phenotypes and it is certainly something we will consider investigating in the future.

2. The mechanism by which TFEB/TFE3 are activated during senescence is unclear. The data shown in Figure 4 (i.e. S142 phosphorylation, CDK4-TFEB interaction, p16 silencing), to support a role for p16/INK4a and CDK4 in TFEB/TFE3 activation, are weak and not convincing. Overall, from the data shown no conclusions can be drawn on the role of CDK4 in the activation of TFEB and TFE3 during senescence. As the authors pointed out, recent evidence suggests that TFEB and TFE3 are controlled by mTORC1 through a substrate-specific mechanism that relies on the activity of RagC/D GTPases (PMID: 32612235, 34253722, 35358174). Importantly, this mechanism is specifically elicited by stimuli that converge on the lysosome, such as lysosomal stress or lysosomal damage (PMID: 32989250, 34597140). Thus, it is surprising that the authors did not attempt to determine whether this pathway plays a role in TFEB activation during senescence. The authors should test whether the expression of constitutively active RagC rescues TFEB/TFE3 localization in senescent cells. Furthermore, they should also verify if the phosphorylation of TFEB S211, a

specific mTORC1 phosphorylation site, is affected during senescence, and whether this phosphorylation can be rescued by the expression of active RagC.

We and others have shown that mTORC1 is constitutively active in senescence and localised to the lysosome (PMID: 28566325, 21512002). RagB and RagC are present on lysosomes (Fig. EV5B-D and PMID: 21512002) in senescence, and overexpression of DN-RagB did not affect lysosomes but did reduce mTORC1 localisation to the lysosome, with a resultant decrease translation of SASP factors (PMID: 21512002). We further show here that the expression of Rag A, B and RagC are not significantly altered in senescence (Fig. EV5B-E), and phosphorylation of TFEB S211 shows only a small reduction in senescence (Fig. 4A). As such, there is little evidence that the mTOR-Rag-TFEB axis is perturbed in senescence. We did however clone and express GFP-RagC^{75L} in primary fibroblasts, followed by induction of senescence as requested. We observed, in support of the published literature, that overexpression of active RagC can sequester TFE3 in the cytoplasm in senescent cells, and we see a significant decrease in p62-Lamp2 colocalisation. This provides further proof of principle that TFEB/TFE3 drive important phenotypes in senescence. Having generated additional evidence for a role of p16-CDK4 axis however, we currently favour this as the primary regulator of TFEB activity in senescence but acknowledge that given the heterogeneity of the senescence programme downstream of different inducers, it is very likely that additional mechanisms play an important role.

3. Tamoxifen, which has been used by the authors to induce the expression of HRasV12 in the oncogene-induced senescence model, is a well-known activator TFEB (PMID: 34411438). Thus, the experiments using 4-OHT are misleading, as 4-OHT treatment per se is known to promote TFEB nuclear translocation even in control cells. The authors should avoid this treatment and use a doxycycline-inducible (or similar) system as a model of oncogene-induced senescence. The mechanistic data proposed in point 2, as well as the other experiments described in the manuscript, should be corroborated in this system and always tested in different models of senescence.

The 4OHT-inducible Ras model of senescence is an established model in the field and has been used in a number of high impact publications (PMID: 21512002, 26280535, 3047484). Furthermore, we have characterised nuclear TFEB in multiple other types of senescence here. We therefore do not believe this is a confounding issue with our model. I appreciate the reviewers concern however and we have carried out a dose-dependent treatment curve of tamoxifen (we use 100nM versus 10µM in PMID: 34411438). We can confirm that the concentrations we use do not activate TFEB (Fig. 1 Rebuttal document). Data presented in

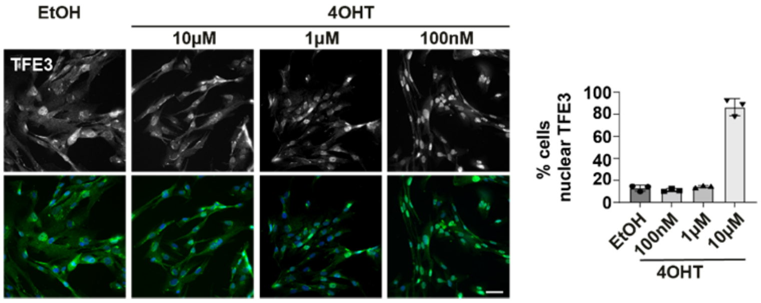

Figure 1 Rebuttal: Cells were treated with 4OHT at the indicated concentration for 6 days. Cells were fixed and immunostained for TFE3. % cells with nuclear TFE3 were quantified.

Figure's 3 and 4 have now been corroborated in additional senescence models.

4. To claim that TFEB is transcriptionally active in senescent cells, the authors only analyzed 3 target genes (i.e. p62, Lamp1, CathB - Fig. 1a). Many more TFEB target genes should be analyzed to make such a claim. It is surprising that the authors performed RNA-seq analyses of senescent cells and never showed whether a global increase in lysosomal/autophagy genes is observed during senescence. The authors should either provide these data or explain why the activation of TFEB/TFE3 does not lead to a global increase of their transcriptional programs.

We apologise for any confusion here. We did not carry out RNA-seq, but rather this has been done previously in this model of senescence. A global increase in both autophagy and lysosome genes was observed (PMID: 19279323). We simply validated a couple of hits here to support the narrative of Figure 1.

5. Quantifications or statistical analyses for most IF data are missing and should be provided upon analysis of numerous cells in different experiments.

Quantification and statistical analysis has been carried out for all panels in the main figures, and where appropriate in the supplementary figures.

6. Page 3: "...genes upregulated in senescence (Fig.1a) are well-known TFEB/TFE3-responsive genes with TFEB/TFE3-binding CLEAR (Co-ordinated Lysosomal Expression and Regulation) motifs". Here an important reference showing TFEB control of lysosomal genes is missing (PMID: 21617040)

This reference has been added

7. Page 3: "...we observed that blocking the CRM1-dependent nuclear export of TFE3 (by treatment with leptomycin B (LMB)) lead to its accumulation over a two-hour time period in proliferating cells. Following the wash-out of LMB, TFE3 was released from the nucleus (Fig. 2b-c)". These observations had been already reported in two independent manuscripts (PMID: 30120233, 29992949). These two references should be cited in this context.

These references have been added

****

Referee #2:

Curnock et al. investigate mechanism and functional consequences of lysosomal biogenesis in senescent cells. By analyzing the proteome, catabolic activity and integrity of lysosomes, the authors provide evidence for lysosomal dysfunction in senescent cells. Investigating the increased lysosomal content of senescent cells in a range of senescence models, the

authors demonstrate a striking nuclear accumulation of the transcription factors TFEB and TFE3, which are master regulators of lysosomal biogenesis. Knockdown of TFEB and TFE3 strongly impairs viability of senescent cells and decreases expression of several senescence-associated proteins. Mechanistically, the authors suggest that TFEB and TFE3 accumulate in the nucleus of senescent cells due to impaired nuclear export through p16-mediated inhibition of CDK4, which normally promotes nuclear export of TFEB and TFE3.

Cellular senescence is closely associated with lysosomal biogenesis, but the underlying mechanisms have been insufficiently understood. The insights of the present study into dysregulated activity of TFEB and TFE3 is thus potentially an important advance in the field of cellular senescence, and which could also be highly relevant for organismal aging and development of senolytic drugs. Nuclear accumulation of TFEB and TFE3 is convincingly demonstrated, but several other conclusions are limited by technical caveats and missing controls. In particular, the mechanistic link between p16, CDK4 and TFEB/TFE3 activity is insufficiently developed. Therefore, the conclusions of the present study are conceptually intriguing but rather speculative at this point, and require substantial additional evidence.

Major concerns:

1. Fig. 1d, e, Suppl. Fig. 1d: While the transcriptional upregulation of lysosomal genes in senescent cells has been well documented (doi: 10.1101/gad.519709), the authors' approach to characterize the lysosomal proteome of senescent cells could yield important novel insights. Unfortunately, the proteomics data are presented on a superficial level, which strongly limits their relevance. I am surprised that the authors only report GO terms, which are difficult to interpret and sometimes contradictory (e.g. various lysosomal hydrolase/catabolism-related GO-terms are both in the upregulated and downregulated categories). It is essential to display changes in selected lysosomal proteins in the figures and provide the whole proteomics dataset in the supplement. To allow the reader to assess the quality of lysosomal enrichment (the FedEx method does not really isolate lysosomes), it would also be very helpful to provide western blot data demonstrating the extent of enrichment of lysosomal proteins, as well as depletion of contamination markers (other organelles, cytosolic proteins).

We have now included a representative blot of the fedex method to demonstrate purity of the lysosomes isolated via this method (Fig. S1A). To assess the occupancy of lysosomal proteins in the prepared lysosomes all identified proteins were uploaded to DAVID functional annotation tool (https://david.ncifcrf.gov/). The top hit of the category of “Cellular Component of Uniprot database” was “Lysosome” with a p-value of 2.4×10^{-24} pointing to high enrichment of lysosomal proteins compared with the human genome used as background. We have further added a graph of selected lysosomal proteins, and presented their relative abundance in senescence, compared to proliferating controls (Figure S1D).

Furthermore, we have added a supplemental table containing the proteomics data of the lysosomal preparations. The proteomics data has been added to the online repository, PRIDE (PXD034358)

2. Fig. 1f-k: The analysis of lysosomal catabolic activity is problematic. Bodipy-Pepstatin A is

a fluorescent conjugate of the cathepsin D inhibitor pepstatin A, which binds to cathepsin D and thereby assays its abundance, but not its activity (doi: 10.1016/s0165-022x(00)00048-8)! To quantify lysosomal proteolytic activity, DQ BSA (and Bodipy-Pepstatin A) is normalized to total lysotracker intensity, which is determined by both lysosomal abundance and lysosomal pH and thus an ambiguous measure. To circumvent this caveat, the authors could normalize DQ BSA to LAMP1 or LAMP2. Alternatively, the authors could quantify DQ BSA by microscopy and normalize to lysotracker area, which would have the additional benefit to illustrate the distribution of proteolytic activity across the lysosomal population. Finally, considering the dramatic increase in cell size that accompanies senescence, I am not sure whether normalization to cell number is appropriate. Critical functions of lysosomes such as turnover of damaged organelles/macromolecules scale with a cell's volume, and normalization to cell size (or area) might thus biologically be a meaningful alternative (or additional) analysis.

We have repeated the flow cytometry data using Texas-Red BSA as a lysosomal loading control for DQ-BSA and Bodipy-PepstatinA. We have added an additional assay, the MagicRed Cathepsin B activity assay. We have also included confocal microscopy of DQ-BSA/TR-BSA and quantification. These modifications and experimental additions confirm our previous conclusions that senescence is associated with a significant increase in lysosome abundance, a trend towards increased lysosomal activity but the important observation that activity, per lysosome is significantly decreased in senescence.

We agree with the reviewer’s last point, and this has been an issue with using flow cytometry assays for any senescence experiments. It was for this reason that we are ‘normalising’ the lysosomal activity with a measure of lysosomal abundance because we were also uncomfortable with standard flow analysis, which is measured per cell. We believe that measuring lysosomal activity relative to abundance is a better analysis and does take into account the larger size of the cells. These data are also corroborated by the proteomics, that indicate the lysosomes in senescence appear essentially ‘dilute’ and that they are lower quality compared to controls.

3. The mechanistic part concerning the model that nuclear export of TFEB/TFE3 is suppressed via p16-mediated inhibition of CDK4 is interesting but rather preliminary and requires experimental substantiation.
Does CDK4 inhibition promote lysosomal biogenesis in proliferating cells?

We can confirm that CDK4 inhibition does induce TFEB nuclear translocation and lysosomal biogenesis (Fig. EV1D, Fig EV3D)

Does p16 shRNA specifically reduce the nuclear pool of TFE3, or does this also affect the cytoplasmic pool?

Knock-down of p16 does not affect total protein levels of TFEB or TFE3 (Fig. 4L, Fig. EV5P).

Does blocking nuclear export restore nuclear TFEB/TFE3 levels in p16-depleted cells?

We hope the reviewer will accept that we decided to carry out this experiment using starvation rather than nuclear export inhibition, to answer the question, does p16 restore

normal function to TFEB? Our previous publications have described how mTORC1 activity is constitutively active in senescence, and thus we were interested to identify whether restoration of normal TFEB-lysosome function may rescue these phenotypes. We can confirm that knock-down of p16 leads to a reduction in mTORC1 activity in senescence (Fig. 4L), and p16shRNA restored nutrient-dependent TFEB nuclear-cytoplasmic shuttling (Fig. EV6A).

Do transcriptional targets of TFEB/TFE3 change at the level of mRNA expression upon the perturbation of CDK4 or p16?

We attempted to isolate mRNA multiple times from cells treated with p16 shRNA but the quality was reproducibly poor. It is currently not clear what the underlying cause could be but as a result we were unable to carry out the qPCR. We have been able to show that shRNA of p16 rescues autophagy-lysosomes at the protein level (Fig. 4L-N).

4. Related to the previous point, the authors state that 'we show that mTORC1-dependent phosphorylation of TFEB at serine 142 is unaltered in senescence'. I assume that the authors mean that TFEB p-S142, which can be phosphorylated by mTORC1, is unaltered in senescence. This is critical, because S142 is also phosphorylated by CDK4, and at least in proliferating cell this is the critical phospho-site through which CDK4 promotes nuclear export of TFEB (DOI: 10.1083/jcb.201911036). Of note, the authors omit CDK4 from the discussion of possible TFEB S142 kinases. I understand that the phosphorylation of TFEB and TFE3 at multiple sites and by several kinases makes an analysis of the relevant phosphorylation sites challenging. However, the authors should at least investigate the effect of CDK4 or mTORC1 inhibition on TFEB p-S142 in proliferating and senescent cells and examine whether TFEB S142A is nuclear or cytoplasmic under these conditions. If S142 is not the relevant phosphosite, other residues that have been identified as CDK4 targets could be analyzed similarly.

We have carried out more thorough quantification of TFEB phosphorylation using Western blotting in proliferating and senescent cells and find that there is a significant reduction in phosphorylation of S142 (~50%) (Fig. 4A). These antibodies could not be validated by IF but confirmed by Western blotting from nuclear/cytoplasmic fractions in proliferating and senescent cells (Fig. EV5).

5. The authors conclude that TFEB/TFE3-dependent lysosomal biogenesis is required for senescence acquisition and survival. Conceptually, these are two distinct processes and it would be interesting to investigate them separately. To examine the relevance of TFEB/TFE3 specifically for senescence acquisition, maybe the authors could genetically deplete TFEB/TFE3 first and subsequently induce senescence?

Knock-down of TFEB/TFE3 prior to induction of senescence leads to widespread cell death. We have now included this data in the manuscript (Fig. 3A, also see Fig. EV4A, B). These data suggest that upregulation of catabolism is required for the initial stages of senescence acquisition.

6. For the genetic loss-of-function experiments, the authors use a single shRNA for each manipulation. Given the well-documented broad off-target effects of shRNAs, one would

expect to use at least two independent shRNAs for each gene and control. Also, please indicate the specific sequences that are used as scrambled controls.

We have now included an additional shRNA for TFEB, TFE3 and CDK2NA/p16 (See EV4-6). The results confirm that the original data were not due to off-target effects. The specific sequence for the scramble control has been included in the methods.

Minor points:
1. Fig. 1: The authors state that 'lysosomal protease-related functional terms were downregulated in the senescent lysosomal preps'. However, based on the qPCR and western blotting data (Fig. 1a, b), CTSB is at least as much increased as LAMP1 in senescent cells. Proteomics and western blotting data for specific cathepsins would be helpful to resolve this apparent contradiction (see also Major Point 1).

We have included blots from the lysosomal preps to demonstrate the purity of the preps. Furthermore, we have included a graph showing the relative abundance of a subset of well-known lysosomal proteins, including the cathepsins in 4OHT-treated cells (all are included in Supplementary Figure 1).

2. Fig. 2: The authors state that 'cytoplasmic-nuclear flux of TFE3 is inhibited in senescence'. Which data support this conclusion? This hypothesis is distinct from the authors' suggestion that a block in nuclear export of TFE3/TFEB leads to their nuclear accumulation.

Please see Figure 2, and nuclear TFEB/TFE3 upon CRM1 inhibition and washout. We have discussed this and are comfortable with our description and interpretation of these data as an inhibition of nuclear-cytoplasmic flux but are happy to change this to nuclear accumulation if the reviewer strongly recommends this.

3. Fig. 3b, g: The authors state that knockdown of TFE3/TFEB reduces the levels of LAMP1. This decrease is not obvious on the shown western blot, and the immunofluorescence data suggest that the related LAMP2 still is strongly upregulated. It would be helpful to demonstrate this decrease more convincingly, and if possible provide additional support with functional assays for lysosomal activity.

The blots have been updated with those from another experimental repeat, as well as a second shRNA and in another model of senescence. We have further included readouts of lysosomal function by confocal microscopy, to measure p62-Lamp2 colocalisation. We agree with the reviewer that the lysosomal phenotypes are not completely resolved. Because we have to do the shRNA transductions after induction of senescence, it may be that it takes more time to resolve those lysosomes that have been produced before shRNA of TFEB/TFE3.

4. Fig. S3: The figure legend is titled 'TFEB/TFE3 is both necessary and sufficient for senescence'. Maybe I missed this, but which data demonstrate sufficiency for senescence induction?

Please also see response to Reviewer 1's comment #1. We have removed this statement and toned down the conclusions that loss of TFEB/TFE3 induces senescence since we have

not comprehensively addressed this. Rather we have replaced this with a statement that loss of TFEB/TFE3 in young, proliferating cells induces markers of increased stress such as DNA damage foci.

5. Fig. S4a: The authors interpret the shift in electrophoretic mobility of TFEB as indicative for decreased phosphorylation. This could easily be confirmed experimentally by phosphatase treatment of the control sample.

We have analysed the phosphorylation of TFEB using phospho-specific TFEB antibodies and find that S142 is significantly reduced in senescence (Fig. 4A).

6. Please include scale bars in all images.

The original scale bar sizes have been increased to make them more visible.

7. The organization of figure panels is sometimes a bit confusing.

The figures have been re-organised, and use of additional EV and supplementary figures should improve the aesthetics and make it easier for the reader.

8. The figure legends are sometimes difficult to read, because several different experiments and techniques are described together.

The figure legends have been modified and simplified.

9. The statistical analyses are sometimes difficult to interpret. Please indicate for each experiment whether n refers to technical or biological replicates. How many images were quantified? With how many cells?

The figure legends have been modified to include all necessary information required to interpret the data.

****

Referee #3:

Summary:
The manuscript by Curnock et al. describes experimental evidence that several models of senescence alters gene and protein expression of lysosomal genes and associated functions. Moreover, they provide evidence that individual lysosomes might be impaired in their function (pH, degradation, damage), but this is offset by an upregulation in total lysosomes, which is proposed to mitigate the loss in lysosomal quality and maintain catabolic activity in senescing cells. They then go on to show that senescence triggers nuclear translocation of TFEB and TFE3, transcription factors known to induce expression of lysosomal, autophagy, and immune-protective genes. They propose that TFEB/TFE3 activation in senescent cells is the mechanisms that upregulates lysosomal levels and offsets lysosome quality loss. They show that silencing of TFEB/TFE3 sensitizes senescent cells to death, suggesting that TFEB/TFE3 activation is protective during senescence. They

further propose that this occurs independently of mTORC1, a known inhibitory regulator of TFEB/TFE3, and instead occurs via p16/INK4a-dependent inhibition of CDK4, which associates with and was previously shown to modulate TFEB. This is an interest manuscript and has some tantalizing observations, but it suffers from a few problems that need to be addressed before publication. If the authors can address most of the issues below, I would support publication of their work in this journal.

Major
1. First, I'll start by criticizing their figure layout. It is messy and confusing. This needs rethinking and reorganization, please! Scale bars are missing.

The original scale bar sizes have been increased to make them more visible. The figures have been re-organised, and use of additional EV and supplementary figures should improve the aesthetics and make it easier for the reader.

2. The authors are inconsistent with their quantification of their data. Some blots are quantified, some are not (Example Fig. 1B, Supl Fig. 1). Some images are, some are not (example, Fig. 1C, 1J, 2G). In all cases, all data should be quantified and statistically analysed.

All blots and images in the figures have been quantified and statistically analysed where applicable.

3. The authors need a section in the Methods that is dedicated to statistical analysis, which should state the assumptions made and the tests used, and why. In fact, the authors used only t-tests even when they have more than two conditions being compared without stating why! Unless there is a specific statistical reason, they should employ tests like ANOVA and post-hoc tests, depending on the assumptions. Then, some graphs have statistical significance indicated as * or its variations, while other graphs state p value like $p<0.3$, which I assume they are trying to argue for a trend. This needs to be more explicitly stated. Moreover, violin plots do not define was is being shown (mean, range, STD?). Some experiments were done $n=2$ (Fig. 3c) or $n=1$ is shown but stats test is done (Fig. 4g). Overall, their statistical analysis is a major weakness, but one that should and can be resolved. In some cases, the authors should consider increasing their experimental repeats to increase robustness.

All statistical analysis have been repeated, and are clearly stated in the legends.

4. Lysotracker Deep Red (LysoT) is used to normalize DQ-BSA and BODIPY-Pepstatin signal. However, the authors show that pH is affected and that LysoT per cell is affected by their treatments. So, unless, I'm missing something, I don't understand how Lysotracker can be used to normalize the proteolysis probes - any effect could be due to pH, not degradation. If DQ-BSA or Pepstatin are not normalized, what is the value? This needs to be revisited. DQ-BSA could be co-endocytosed with a dye like dextran to normalize for uptake and trafficking to lysosomes. As shown, there may be other interpretations of their data other than that lysosome proteolysis is impaired by senescence.

The non-normalised data for DQ-BSA and BODIPY-Pepstatin are included in Figure EV1E-G). We have repeated these assays with Texas-red BSA as a better control than LysoT. We have also included confocal microscopy (and quantification) of DQ-BSA/TR-BSA and an additional assay, MagicRed Cathepsin B.

5. The co-IP between TFEB-GFP and CDK4 is not convincing without quantification and indication of repeats in the legend. To boost the confidence in this result, the authors should reverse the co-IP by pulling down CDK4 and probing for TFEB-GFP.

Quantification of this IP has now been included (Fig. 4D,E and EV5F-I). We have subcloned TFEB and CDK4 to carry out the reverse IPs. Due to the fact that the nuclear envelope in senescent cells is more fragile than controls (loss of LaminB is a hallmark of senescence), we were unable to get reproducible data for the reverse IPs from nuclear fractions. Instead, we have repeated the experiments using whole cell lysates (Fig. 4D,E). These data confirm the previous findings included in the manuscript, that there is less CDK4-TFEB interaction in senescence.

6. The most significant issue in my opinion is as follows. All treatments used to cause senescence are complex and can result in other events that promote TFEB or lysosome "aging". While the authors are to be commended for trying many approaches to induce senescence, this falls short of aging-related senescence. I think it is not unreasonable to test if generational aging of cells like MRC-5 (30-40 generations) induces TFEB translocation, affects lysosome function, and lysosome levels. I am cognizant that this takes time, but the authors could maintain these cells over multiple generations as they address other issues and then test this. This is a clear flaw that reduces the impact of their work towards aging senescence rather than induced senescence.

We have now included data on TFEB translocation (Fig. EV3B), lysosomal abundance (EV1B) and lysosomal function (Fig. EV1H-J) from replicative senescent MRC5s. We can confirm that they demonstrate all of the same phenotypes as the more acute models of senescence i.e. an accumulation of less active lysosomes. We thank the reviewer for this comment because the replicative senescent model shows robust changes in the lysosome content and function, as well as nuclear accumulation of TFEB/TFE3 and thus going forward, we will certainly utilise this model more.

Minor/Non-essential suggestions

1. Authors focus on lysosome number/amount, but their images indicate a significant impact on lysosome position (or this appears to be the case based on their images). Lysosome position can impact function - the authors should quantify images to determine if lysosome distribution is also an affected of treatments.

This is an extremely interesting point and we can say that indeed, induction senescence leads a very tight clustering of lysosomes and colocalisation with the autophagy marker, p62 (Fig 3H, EV4G,M,S). The lysosomal clustering and p62-Lamp2 colocalisation are resolved by knock-down of p16 (Fig. 4 and EV5-6) or TFEB/TFE3 (Fig. 3, EV4) which is accompanied with a redistribution of lysosomes (not quantified). We have also previously seen that lysosomal distribution in response to nutrients is perturbed in senescence (Fig. 2 Rebuttal

document). We would like to investigate this latter point more comprehensively, so we have decided not to include this data in the current manuscript but include it in a follow-up manuscript.

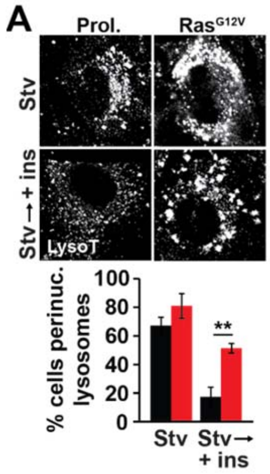

Figure 2 Rebuttal: Nutrient-dependent changes in lysosomal positioning is altered in senescence. Cells were starved of growth factors and amino acids (Stv), or starved and refed insulin (ins) for 30min. Live cell imaging of Lysotracker was carried out and analysis of lysosome velocity (not shown) and positioning was quantified.

1st Revision - Editorial Decision 19th Oct 2022

Dear Bernadette,

Thank you for the careful revisions you have made to your manuscript. As you will see in the re-review reports attached to the bottom of this mail, they have gone a long way to addressing the concerns of the referees.

However, there remains significant doubt over the extent to which the sequesteration of TFEB in the cytosol is driven by its phosphorylation by CDK4 (as opposed to by mTORC1). I agree with the reviewers 1 and 2 that, as a prominent mechanistic aspect of your study, this issue needs to be clarified before we can proceed towards publication of your work in EMBO Journal.

Therefore, given the extent to which you have satisfied the referees other concerns, and given your work's novelty and potential to generate a high level of interest, in this exceptional case I would like to invite you to focus on referee 1's concerns in a second round of revision.

When preparing your letter of response to the referees' comments, please bear in mind that this will form part of the Review Process File, and will therefore be available online to the community. For more details on our Transparent Editorial Process, please visit our website: https://www.embopress.org/page/journal/14602075/authorguide#transparentprocess

If you have any concerns, I would be happy to discuss your revisions in a Zoom call. Please send me an email if you would like to take this offer up.

When preparing your letter of response to the referees' comments, please bear in mind that this will form part of the Review Process File, and will therefore be available online to the community. For more details on our Transparent Editorial Process, please visit our website: https://www.embopress.org/page/journal/14602075/authorguide#transparentprocess

We generally allow three months as standard revision time. As a matter of policy, competing manuscripts published during this period will not negatively impact on our assessment of the conceptual advance presented by your study. However, we request that you contact the editor as soon as possible upon publication of any related work, to discuss how to proceed. Should you foresee a problem in meeting this three-month deadline, please let us know in advance and we may be able to grant an extension.

Thank you for the opportunity to consider your work for publication. I look forward to your revision.

Best wishes,

William

William Teale, PhD
Editor
The EMBO Journal
w.teale@embojournal.org

Instructions for preparing your revised manuscript:

Please make sure you upload a letter of response to the referees' comments together with the revised manuscript.

Please also check that the title and abstract of the manuscript are brief, yet explicit, even to non-specialists.

When assembling figures, please refer to our figure preparation guideline in order to ensure proper formatting and readability in print as well as on screen:
https://bit.ly/EMBOPressFigurePreparationGuideline
See also guidelines for figure legends: https://www.embopress.org/page/journal/14602075/authorguide#figureformat

At EMBO Press we ask authors to provide source data for the main and EV figures. Our source data coordinator will contact you to discuss which figure panels we would need source data for and will also provide you with helpful tips on how to upload and organize the files.

IMPORTANT: When you send the revision we will require
- a point-by-point response to the referees' comments, with a detailed description of the changes made (as a word file).
- a word file of the manuscript text.

- individual production quality figure files (one file per figure)
- a complete author checklist, which you can download from our author guidelines (https://www.embopress.org/page/journal/14602075/authorguide).
- Expanded View files (replacing Supplementary Information)
Please see out instructions to authors
https://www.embopress.org/page/journal/14602075/authorguide#expandedview

Please remember: Digital image enhancement is acceptable practice, as long as it accurately represents the original data and conforms to community standards. If a figure has been subjected to significant electronic manipulation, this must be noted in the figure legend or in the 'Materials and Methods' section. The editors reserve the right to request original versions of figures and the original images that were used to assemble the figure.

Further information is available in our Guide For Authors: https://www.embopress.org/page/journal/14602075/authorguide

We realize that it is difficult to revise to a specific deadline. In the interest of protecting the conceptual advance provided by the work, we recommend a revision within 3 months (17th Jan 2023). Please discuss the revision progress ahead of this time with the editor if you require more time to complete the revisions. Use the link below to submit your revision:

https://emboj.msubmit.net/cgi-bin/main.plex

Referee #1:

The authors addressed most of my previous concerns. However, there are a few key points that still require further elucidation:

1. Mechanism of TFEB activation during senescence:
The mechanism underlying TFEB de-phosphorylation during senescence, which is a very important aspect of the current manuscript, has not been addressed properly. The authors propose that CDK4 inhibition during senescence causes TFEB de-phosphorylation at S142 and subsequent nuclear translocation, whereas mTORC1 activity is not affected. However, the model proposed is not convincing for the following reasons:
a. The evidence that pharmacological inhibition of CDK4 causes TFEB nuclear translocation is correlative and does not indicate that CDK4 inhibition drives TFEB activation during senescence. The authors never showed that CDK4 is inactive in senescence. To this end, CDK4 activity should be analyzed in depth upon senescence induction (either by in vitro assays or by measuring the phosphorylation of known CDK4 substrates). Importantly, the authors should demonstrate that forcing CDK4 re-activation during senescence is sufficient to promote TFEB re-phosphorylation and cytosolic localization. As a matter of fact, the authors show that the expression of a constitutively active form of RagC (RagC S75L), which promotes the mTORC1-mediated phosphorylation of TFEB without affecting CDK4 activity, is sufficient to restore TFEB cytosolic localization during senescence. These data argue against a possible role for CDK4 in the modulation of TFEB and strongly suggest that loss of mTORC1-mediated phosphorylation of TFEB promotes its nuclear translocation during senescence (see point b below).
b. The authors state that the Rag-mTORC1-TFEB axis is intact because "lysosomal localization and protein expression levels of endogenous Rag GTPases are not perturbed in senescence (Fig EV5B-E), and consistent with previous reports, mTORC1 is active (Fig EV5D) (Carroll et al. 2017; Narita et al., 2011) and has been shown to localize to lysosomes (Carroll et al. 2017; Narita et al., 2011)". These data, however, do not exclude that mTORC1 activity towards TFEB is impaired during senescence. First, the evidence that lysosomal localization and protein expression levels of endogenous Rag GTPases are not perturbed in senescence provides no information about the activity of this pathway. For instance, nutrient availability, a major controller of mTORC1 activity via the Rag GTPases, does not affect localization and expression of Rag GTPases, rather it controls their activity (i.e. their nucleotide binding state). Thus, in the absence of nutrients, localization and protein expression levels of Rag GTPases are not perturbed, yet Rag GTPases are inactive and unable to mediate mTORC1 activation. Second, recent evidence has shown that, in the Rag GTPase dimer, the activation of RagA/B is required for mTORC1 lysosomal recruitment and phosphorylation of all mTORC1 substrates (e.g. TFEB/S6K/4E-BP1), whereas the activation status of RagC/D is crucial for the mTORC1-mediated phosphorylation of TFEB/TFE3 but does not affect mTORC1 activity towards S6K/4E-BP1 (PMID: 32612235, 35358174). Thus, stimuli that induce specific inactivation of RagC/D impair the mTORC1-mediated phosphorylation of TFEB without affecting mTORC1 lysosomal localization or its activity towards S6K/4E-BP1 (PMID: 32612235, 35358174, 32989250, 34597140, 34253722). The data in the present manuscript showing that the expression of a constitutively active form of RagC rescues TFEB subcellular localization (Fig EV4I,J) strongly suggests that RagC gets inactivated during senescence, thus promoting specific loss of mTORC1 activity towards TFEB. The evidence shown in this manuscript that senescence induction promotes lysosomal stress, strongly supports this scenario, as the same type of stress has been found as a major cue that promotes RagC inactivation (PMID: 32989250, 34597140). Interestingly, p16 knockdown seems to relieve the lysosomal phenotype observed upon 4-OHT treatment (Fig 4H and EV5L), suggesting that loss of p16 may prevent 4-OHT-mediated lysosomal stress and subsequent RagC inactivation. This could be tested by assessing whether senescence induction promotes loss of TFEB-RagC binding (a major readout of RagC activity) and whether p16 knockdown rescues such interaction. Finally, the

authors should test if the expression of constitutively active RagC, in addition to rescuing TFEB subcellular localization, also rescues S211/S142 phosphorylation, without affecting the activity of CDK4.
c. The authors state that S211 phosphorylation of TFEB only shows a 20% reduction during senescence, whereas S142 shows a 50% reduction, which supports, according to the authors, a possible impairment of CDK4/6 (an S142 kinase), rather than mTORC1 (an S211 kinase), during senescence. However, it has been previously shown that TFEB undergoes a hierarchical phosphorylation and that S142 phosphorylation is required for subsequent phosphorylation of TFEB at S211 (PMID: 30120233, 29764979). This implies that a reduction in S142 phosphorylation should also correlate with a similar reduction of S211. Thus, the differences observed by the authors between S142 and S211 phosphorylation are likely due to a different sensitivity of the two phosphoantibodies, rather than a selective inhibition of S142 phosphorylation. Otherwise, how can a TFEB molecule, de-phosphorylated at S142 and therefore nuclear, be phosphorylated on S211 by mTORC1 at the lysosome, as proposed by the authors? Please note that S142 is a promiscuous phosphorylation site and has been shown to be phosphorylated by several kinases, including mTORC1 (PMID: 21617040, 31733992, 22343943).
Finally, I am quite puzzled with the images shown in Figures EV4F,H. The authors show that CDK inhibitors (Fig EV4F) and 4-OHT (Fig EV4H) both impair the interaction between TFEB and CDK4. However, the same inhibitors, in the same experimental conditions, do not impair S142 phosphorylation, nor they promote an increase in the amount of nuclear TFEB (Figures EV4F,H). How do the authors explain these discrepancies? In my opinion these data are weak and are not sufficient to support a role for CDK4 in TFEB activation during senescence.

2. For many experiments, n is <3. In some important experiments, n=1. This needs to be fixed.
Moreover, for IF experiments, it is unclear what the n refers to (number of fields? Number of experiments? Number of cells?). To get meaningful statistical data, each IF experiment needs to be quantified by counting dozens or hundreds of cells in multiple fields (i.e. at least 5-6 fields per experiment). To better represent the variability observed in these experiments, results from individual fields or cells should be plotted in the graphs. Similar results need to be obtained in at least 3 different experiments.

3. Figure legends are not detailed enough to allow the reader to understand how the experiment described was performed.

Referee #2:

The revised manuscript, Curnock et al. present additional data that strengthen the author's conclusions. In particular, the authors perform additional assays for lysosomal function, include a second shRNA for genetic knockdown experiments and analyze the relationship between TFEB/TFE3 and p16/CDK4 in more detail. Moreover, the re-organization and presentation of figures and legends substantially increases their clarity and comprehensibility. I am generally satisfied with the additional experiments and in principle consider this an interesting manuscript for the readership of EMBO Journal. However, a few remaining issues should be addressed in the manuscript or, where applicable, with additional experiments.

1. The authors include TR-BSA as a lysosomal loading control to infer changes in lysosomal proteolytic capacity relative to lysosomal content. However, TR-BSA is a readout for the rate of endocytosis and only indirectly reflects lysosomal content. The comparison of DQ BSA / TR-BSA indicates that the fraction of degraded / endocytosed BSA decreases in senescent cells (which may be due to changes in endocytosis and/or lysosomal content). While this generally supports the author's narrative, if they want to make a statement on lysosomal activity relative to lysosomal content, they should quantify lysosomal content directly, using e.g. Lamp1/2 or quantifying lysosomal area.

2. The conclusion that lysosomes are more 'dilute' in senescent cells is a bit strange. Perhaps, a decrease in proteolytic capacity leads to accumulation of undigested proteins in lysosomes, causing an apparent relative decrease in the abundance of lysosomal proteins in the proteomics experiment? In general, the presentation of the proteomics data remain suboptimal. One normally would expect a volcano plot or a similar presentation that depicts information on change in abundance and significance for individual proteins. Such a graph would also allow an intuitive presentation of changes in lysosomal vs. non-lysosomal proteins. This is up to the authors to decide, but can only repeat that the proteomics data are difficult to appreciate in their current presentation.

3. I still find it difficult to follow the author's argument that cytoplasmic-nuclear flux of TFE3 is inhibited in senescence. The authors show that TFE3 is constitutively nuclear in senescent cells - conceivably, TFE3 doesn't show increased cytoplasmic-nuclear flux upon starvation, simply because it already localizes to the nucleus. Maybe this is a misunderstanding due to ambiguous wording? Cytoplasmic-nuclear flux implies a directional flux from cytosol to nucleus. If the authors refer to inhibition of flux between cytoplasm and nucleus, without specifying directionality, they should word this more precisely.

4. The data on a role of CDK4-mediated TFEB S142 phosphorylation remain correlative. If the authors want to draw any mechanistic conclusions on the relevance of this phospho-site, they should compare subcellular localization of TFEB and TFEB S142A during senescence, inhibition of CDK4 and mTORC1.

Referee #3:

The authors have addressed my concerns. This work is an extraordinary feat. The work should be published without further delay as it will be of great interest to scientists interested in lysosome biology, cellular adaptation, cellular senescence and aging across a number of biological functions like neurobiology, immunity, and cardiovascular function.

Dear William,

Thank you for the comments on our revised manuscript, we are pleased that our additional experiments have gone a long way to gain the Reviewers' support for publication. We thank Reviewers 1 and 2 for their additional comments on the manuscript. We concede that the specific mechanism responsible for TFEB/TFE3 nuclear accumulation in senescence has not been fully established. Instead, there is evidence that multiple pathways may contribute, including dysregulation of the CDK4-TFEB axis and RagC-TFEB axis, leading to hypophosphorylation of TFEB/TFE3. We have modified the abstract, results and discussion to reflect this, and have included a graphical abstract summarising our findings (see below).

We are confident that the focus of the manuscript, 'TFEB/TFE3-dependent lysosome biogenesis is required for senescence' will still provide an important addition to the field. We have characterised one of the gold standard markers for senescence and have established for the first time that nuclear accumulation of TFEB/TFE3 could be considered a new hallmark of senescence.

We hope the Reviewers will support these changes and publication of the manuscript.

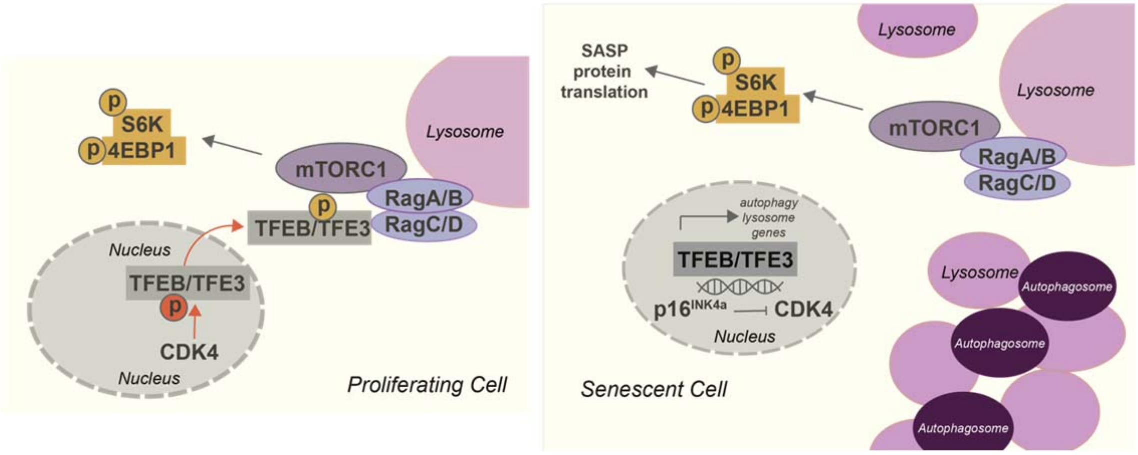

Graphical abstract: *In proliferating cells, TFEB-dependent expression of autophagy-lysosome genes is regulated by multiple phosphorylation events that regulate the subcellular localisation of TFEB/TFE3. Cellular senescence is associated with an increase in the autophagy-lysosome pathway at the mRNA and protein level. We demonstrate that lysosomes in senescence are less efficient; they have increased pH and show signs of damage. Our data indicate that compensatory upregulation of lysosome biogenesis is driven by TFEB/TFE3. Experimental evidence suggests that dysregulation of multiple pathways may contribute to the hypo-phosphorylation and constitutive nuclear localisation of TFEB/TFE3 in the nucleus, including CDK4 and mTORC1.*

Specific Comments

Reviewer 1

The mechanism underlying TFEB de-phosphorylation during senescence, which is a very important aspect of the current manuscript, has not been addressed properly. The authors propose that CDK4 inhibition during senescence causes TFEB de-phosphorylation at S142 and subsequent nuclear translocation, whereas mTORC1 activity is not affected. However, the model proposed is not convincing for the following reasons:

a. The evidence that pharmacological inhibition of CDK4 causes TFEB nuclear translocation is correlative and does not indicate that CDK4 inhibition drives TFEB activation during senescence. The authors never showed that CDK4 is inactive in senescence. To this end, CDK4 activity should be analyzed in depth upon senescence induction (either by in vitro assays or by measuring the phosphorylation of known CDK4 substrates). Importantly, the authors should demonstrate that forcing CDK4 re-activation during senescence is sufficient to promote TFEB re-phosphorylation and cytosolic localization.

We believe that we have perhaps not been clear in the text about the senescence programme. The classic definition of cellular senescence is 'an irreversible exit from the cell cycle'. Downstream of multiple stressors, senescence is induced and maintained by the upregulation of cyclin-dependent kinase inhibitors, most notably $p21^{CIP}$ and $p16^{INK4a}$ which directly interact and inhibit the cyclin-dependent kinases, CDK4/6. The inactivation of cyclin-dependent kinases is considered a defining feature of senescence which is why we did not feel the need to demonstrate this in the manuscript. We have now added a blot demonstrating the CDK4 substrate pRb is reduced in senescence. We utilised shRNA targeting $p16^{INK4a}$ to relieve CDK4 inhibition and essentially to re-activate it, which we have now confirmed by demonstrating an increase in phosphorylation of Rb (Suppl. Fig 4I,J).

As a matter of fact, the authors show that the expression of a constitutively active form of RagC (RagC S75L), which promotes the mTORC1-mediated phosphorylation of TFEB without affecting CDK4 activity, is sufficient to restore TFEB cytosolic localization during senescence. These data argue against a possible role for CDK4 in the modulation of TFEB and strongly suggest that loss of mTORC1-mediated phosphorylation of TFEB promotes its nuclear translocation during senescence (see point b below).

b. The authors state that the Rag-mTORC1-TFEB axis is intact because "lysosomal localization and protein expression levels of endogenous Rag GTPases are not perturbed in senescence (Fig EV5B-E), and consistent with previous reports, mTORC1 is active (Fig EV5D) (Carroll et al. 2017; Narita et al., 2011) and has been shown to localize to lysosomes (Carroll et al. 2017; Narita et al., 2011)". These data, however, do not exclude that mTORC1 activity towards TFEB is impaired during senescence. First, the evidence that lysosomal localization and protein expression levels of endogenous Rag GTPases are not perturbed in senescence provides no information about the activity of this pathway. For instance, nutrient availability, a major controller of mTORC1 activity via the Rag GTPases, does not affect localization and expression of Rag GTPases, rather it controls their activity (i.e. their nucleotide binding state).

Thus, in the absence of nutrients, localization and protein expression levels of Rag GTPases are not perturbed, yet Rag GTPases are inactive and unable to mediate mTORC1 activation. Second, recent evidence has shown that, in the Rag GTPase dimer, the activation of RagA/B is required for mTORC1 lysosomal recruitment and phosphorylation of all mTORC1 substrates (e.g. TFEB/S6K/4E-BP1), whereas the activation status of RagC/D is crucial for the mTORC1-mediated phosphorylation of TFEB/TFE3 but does not affect mTORC1 activity towards S6K/4E-BP1 (PMID: 32612235, 35358174). Thus, stimuli that induce specific inactivation of RagC/D impair the mTORC1-mediated phosphorylation of TFEB without affecting mTORC1 lysosomal localization or its activity towards S6K/4E-BP1 (PMID: 32612235, 35358174, 32989250, 34597140, 34253722). The data in the present manuscript showing that the expression of a constitutively active form of RagC rescues TFEB subcellular localization (Fig EV4I,J) strongly suggests that RagC gets inactivated during senescence, thus promoting specific loss of mTORC1 activity towards TFEB. The evidence

shown in this manuscript that senescence induction promotes lysosomal stress, strongly supports this scenario, as the same type of stress has been found as a major cue that promotes RagC inactivation (PMID: 32989250, 34597140). Interestingly, p16 knockdown seems to relieve the lysosomal phenotype observed upon 4-OHT treatment (Fig 4H and EV5L), suggesting that loss of p16 may prevent 4-OHT-mediated lysosomal stress and subsequent RagC inactivation.

This could be tested by assessing whether senescence induction promotes loss of TFEB-RagC binding (a major readout of RagC activity) and whether p16 knockdown rescues such interaction.

Finally, the authors should test if the expression of constitutively active RagC, in addition to rescuing TFEB subcellular localization, also rescues S211/S142 phosphorylation, without affecting the activity of CDK4.

We agree with the Reviewer that the nucleotide loading (or cycling) of RagC could be perturbed in senescence with little impact on the co-localisation of mTOR or Rags with lysosomes. And as the Reviewer indicates, the rescue of TFEB localisation upon overexpression of active RagC supports this hypothesis. We have therefore modified the manuscript text to make it explicit that RagC inactivation may act as the key driver of nuclear TFEB translocation in senescence.

We would request not carry out the suggested experiment (RagC-TFEB IP in p16shRNA-treated cells) as we are concerned that they will be extremely technically challenging in our model (given that it will involve the simultaneous viral transduction of a 2 transgenes and shRNA). However, as suggested, we have assessed the phosphorylation of TFEB/TFE3 and Rb (downstream of CDK4) in active RagC overexpressing cells. We can confirm there is no effect of RagC overexpression on CDK4 activity (as measured by phosphorylation of Rb) and shown in this rebuttal (Fig.1). Intriguingly, the phosphorylation of TFEB is not rescued in RagC-expressing cells, despite a rescue of the subcellular localisation (modified version of manuscript Fig EV6F-H). It is currently unclear why either overexpression of RagC or p16shRNA are both able to robustly rescue the subcellular localisation of TFEB/TFE3 without rescuing the phosphorylation, but suggests the dysregulation of multiple pathways contributes to TFEB/TFE3 activation in senescence. As mentioned above, we have explicitly outlined that the mechanism of TFEB/TFE3 activation in senescence remains to be fully elucidated.

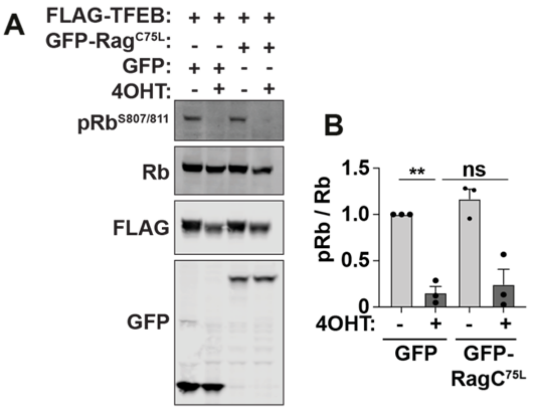

Rebuttal Fig.1: RagC^{75L} overexpression does not affect CDK4 activity in control or senescent cells. ***A.*** Cells were transduced with GFP or GFP-RagC^{75L} in EtOH or 4OHT conditions and immunoblotted for the CDK4 substrate, pRB. ***B.*** Blots were quantified. (n=3) All graphs show individual data points, mean and error bars represent standard deviation. All data analysed by one-way ANOVA with Tukey's multiple comparison test (* <0.05, ** <0.01, *** <0.001)

c. The authors state that S211 phosphorylation of TFEB only shows a 20% reduction during senescence, whereas S142 shows a 50% reduction, which supports, according to the authors, a possible impairment of CDK4/6 (an S142 kinase), rather than mTORC1 (an S211 kinase), during senescence. However, it has been previously shown that TFEB undergoes a hierarchical phosphorylation and that S142 phosphorylation is required for subsequent phosphorylation of TFEB at S211 (PMID: 30120233, 29764979). This implies that a reduction in S142 phosphorylation should also correlate with a similar reduction of S211. Thus, the differences observed by the authors between S142 and S211 phosphorylation are likely due to a different sensitivity of the two phosphoantibodies, rather than a selective inhibition of S142 phosphorylation. Otherwise, how can a TFEB molecule, de-phosphorylated at S142 and therefore nuclear, be phosphorylated on S211 by mTORC1 at the lysosome, as proposed by the authors? Please note that S142 is a promiscuous phosphorylation site and has been shown to be phosphorylated by several kinases, including mTORC1 (PMID: 21617040, 31733992, 22343943).

As someone with extensive expertise on TFEB, we concede the Reviewer is more knowledgeable re. the reagents to measure TFEB phosphorylation. We have changed the text to state that TFEB shows significant dephosphorylation at both S142 and S211.

Finally, I am quite puzzled with the images shown in Figures EV4F,H. The authors show that CDK inhibitors (Fig EV4F) and 4-OHT (Fig EV4H) both impair the interaction between TFEB and CDK4. However, the same inhibitors, in the same experimental conditions, do not impair S142 phosphorylation, nor they promote an increase in the amount of nuclear TFEB (Figures EV4F,H). How do the authors explain these discrepancies? In my opinion these data are weak and are not sufficient to support a role for CDK4 in TFEB activation during senescence.

The data referred to here were reliant on nuclear fractionation which in senescence is extremely unreliable. This is because there are fundamental differences in the nuclear compartment of control versus senescent cells, where a loss of LaminB1 is a hallmark of the latter. As a result, isolation of nuclear fractions is technically more difficult, and a lot of material is lost upon sequential washes. We have therefore removed these data to avoid any confusion.

2. For many experiments, n is <3. In some important experiments, n=1. This needs to be fixed.

Moreover, for IF experiments, it is unclear what the n refers to (number of fields? Number of

experiments? Number of cells?). To get meaningful statistical data, each IF experiment needs to be quantified by counting dozens or hundreds of cells in multiple fields (i.e. at least 5-6 fields per experiment). To better represent the variability observed in these experiments, results from individual fields or cells should be plotted in the graphs. Similar results need to be obtained in at least 3 different experiments.

We have modified all of the figure legends to include more specific details about image analysis and make it clear that indeed our analyses are based on dozens of cells from multiple fields per experiment, all repeated experimentally.

There are a number of experiments n=2 but these are primarily limited to validation experiments (with second shRNAs) or IPs. We have made sure that more variable experiments, in particular the confocal microscopy/co-localisation experiments have been repeated at least 3, and in most cases 4 times.

3. Figure legends are not detailed enough to allow the reader to understand how the experiment described was performed.

We have carefully gone through the legends and where necessary, have added more detail.

Reviewer 2

The revised manuscript, Curnock et al. present additional data that strengthen the author's conclusions. In particular, the authors perform additional assays for lysosomal function, include a second shRNA for genetic knockdown experiments and analyze the relationship between TFEB/TFE3 and p16/CDK4 in more detail. Moreover, the re-organization and presentation of figures and legends substantially increases their clarity and comprehensibility. I am generally satisfied with the additional experiments and in principle consider this an interesting manuscript for the readership of EMBO Journal. However, a few remaining issues should be addressed in the manuscript or, where applicable, with additional experiments.

1. The authors include TR-BSA as a lysosomal loading control to infer changes in lysosomal proteolytic capacity relative to lysosomal content. However, TR-BSA is a readout for the rate of endocytosis and only indirectly reflects lysosomal content. The comparison of DQ BSA / TR-BSA indicates that the fraction of degraded / endocytosed BSA decreases in senescent cells (which may be due to changes in endocytosis and/or lysosomal content). While this generally supports the author's narrative, if they want to make a statement on lysosomal activity relative to lysosomal content, they should quantify lysosomal content directly, using e.g. Lamp1/2 or quantifying lysosomal area.

We have added a caveat to the results section to describe exactly what DQ BSA/ TR-BSA represents.

2. The conclusion that lysosomes are more 'dilute' in senescent cells is a bit strange. Perhaps, a decrease in proteolytic capacity leads to accumulation of undigested proteins in lysosomes, causing an apparent relative decrease in the abundance of lysosomal proteins in the proteomics experiment? In general, the presentation of the proteomics data remain suboptimal. One normally would expect a volcano plot or a similar presentation that depicts information on change in abundance and significance for individual proteins. Such a graph would also allow an intuitive presentation of changes in lysosomal vs. non-lysosomal

proteins. This is up to the authors to decide, but can only repeat that the proteomics data are difficult to appreciate in their current presentation.

We thank the Reviewer for their input on the presentation of this data. We have sought advice from colleagues and we are content that the relative abundance graph, showing that many lysosomal proteins are decreased in senescence demonstrates the important findings from this dataset.

3. I still find it difficult to follow the author's argument that cytoplasmic-nuclear flux of TFE3 is inhibited in senescence. The authors show that TFE3 is constitutively nuclear in senescent cells - conceivably, TFE3 doesn't show increased cytoplasmic-nuclear flux upon starvation, simply because it already localizes to the nucleus. Maybe this is a misunderstanding due to ambiguous wording? Cytoplasmic-nuclear flux implies a directional flux from cytosol to nucleus. If the authors refer to inhibition of flux between cytoplasm and nucleus, without specifying directionality, they should word this more precisely.

We have modified the wording to make it more precise and avoid any confusion.

4. The data on a role of CDK4-mediated TFEB S142 phosphorylation remain correlative. If the authors want to draw any mechanistic conclusions on the relevance of this phospho-site, they should compare subcellular localization of TFEB and TFEB S142A during senescence, inhibition of CDK4 and mTORC1.

We agree with the Reviewer (and Reviewer 1) that the mechanism of TFEB/TFE3 activation in senescence has not been fully elucidated. We have modified the text to reflect this and in particular, have highlighted the potential role of RagC-mTORC1-TFEB axis.

2nd Revision - Editorial Decision 1st Feb 2023

Dear Berni,

Thank you for submitting the revised version of your manuscript, which takes into account the concerns of referees 1 and 2. Referee 1 was particularly careful to note that your data about the involvement of CDK4 in the nuclear-localisation of TFEB during cell senescence do not rule out the possibility of it being under the influence of the RagC-mTOR regulatory axis. Thank you for incorporating this hypothesis throughout the manuscript, and discussing your data in the context of contributions from diverse pathways converging on the regulation of TFEB localization. Before I can formally accept your manuscript for publication, however, there are some remaining editorial points which need to be addressed. In this regard would you please:

• Rename the Conflict of Interest Section as the "DISCLOSURE AND COMPETING INTERESTS STATEMENT" according to the instructions on our website,
• remove the AC/CRediT section from the manuscript text
• rename Supplemental Table 1 as 'Dataset EV1' with the corresponding callout in the main text. The legend should be uploaded as a separate tab of the Excel file,
• assemble Supplementary Figures in one Appendix file with a table of contents (with page numbers); please rename these files as Appendix Figure S1-S4 with the appropriate callouts in the main text,
• remove error bars wherever n=2 (Fig EV1, Fig EV4J, Supp Fig 2E and G, Supp Fig 3B-D, H-J, Fig 4E, Fig EV5C, E, G and K, Fig EV6A). Please also see further comments on this in the data edited MS file (.doc),
• check zoom boxes are correctly positioned in Fig 4F, Fig 4H and Fig EV4
• ensure dataset PXD034358 is made publicly available
• check the author email address katy.yalci@bristol.ac.uk is accessible.

We require the publication of source data for electrophoretic gels and blots, with the aim of making primary data more accessible and transparent to the reader. Please therefore provide me with a PDF file per figure that contains the original, uncropped and unprocessed scans of all or key gels used in the figures. The PDF files should be labeled with the appropriate figure/panel number, and should have molecular weight markers; further annotation could be useful but is not essential. The PDF files will be published online with the article as supplementary "Source Data" files. Source Data can also include Excel tables to accompany your graphs. We anticipate that their inclusion will make your work more discoverable and usable to scientists in the future. Hannah Sonntag will contact you separately about the Source Data for this manuscript.

We include a synopsis of the paper (see http://emboj.embopress.org/). Please provide me with a general summary statement and 3-5 bullet points that capture the key findings of the paper.
We also need a summary figure for the synopsis. The size should be 550 wide by [200-400] high (pixels). You can also use something from the figures if that is easier.

EMBO Press is an editorially independent publishing platform for the development of EMBO scientific publications.

Best wishes,

William

William Teale, PhD
Editor
The EMBO Journal
w.teale@embojournal.org

Use the link below to submit your revision:

https://emboj.msubmit.net/cgi-bin/main.plex

All editorial and formatting issues were resolved by the authors.

3rd Revision - Editorial Decision 21st Feb 2023

Dear Berni,

I am pleased to inform you that your manuscript has been accepted for publication in the EMBO Journal.

Congratulations on some really exciting science!

Please note that it is EMBO Journal policy for the transcript of the editorial process (containing referee reports and your response letter) to be published as an online supplement to each paper. If you do NOT want this, you will need to inform the Editorial Office via email immediately. More information is available here:
https://www.embopress.org/page/journal/14602075/authorguide#transparentprocess

Your manuscript will be processed for publication in the journal by EMBO Press. Manuscripts in the PDF and electronic editions of The EMBO Journal will be copy edited, and you will be provided with page proofs prior to publication. Please note that supplementary information is not included in the proofs.

You will be contacted by Wiley Author Services to complete licensing and payment information. The required 'Page Charges Authorization Form' is available here: https://www.embopress.org/pb-assets/embo-site/tej_apc.pdf - please download and complete the form and return to embopressproduction@wiley.com

EMBO Press participates in many Publish and Read agreements that allow authors to publish Open Access with reduced/no publication charges. Check your eligibility: https://authorservices.wiley.com/author-resources/Journal-Authors/open-access/affiliation-policies-payments/index.html

Should you be planning a Press Release on your article, please get in contact with embojournal@wiley.com as early as possible, in order to coordinate publication and release dates.

If you have any questions, please do not hesitate to call or email the Editorial Office. Thank you for your contribution to The EMBO Journal.

Best wishes,

William

William Teale, PhD
Editor
The EMBO Journal
w.teale@embojournal.org

** Click here to be directed to your login page: https://emboj.msubmit.net

EMBO Press Author Checklist

Corresponding Author Name: Bernadette Carroll	
Journal Submitted to: EMBO J	
Manuscript Number: EMBOJ-2022-111241	

USEFUL LINKS FOR COMPLETING THIS FORM
The EMBO Journal - Author Guidelines
EMBO Reports - Author Guidelines
Molecular Systems Biology - Author Guidelines
EMBO Molecular Medicine - Author Guidelines

Reporting Checklist for Life Science Articles (updated January
This checklist is adapted from Materials Design Analysis Reporting (MDAR) Checklist for Authors. MDAR establishes a minimum set of requirements in transparent reporting in the life sciences (see Statement of Task: 10.31222/osf.io/9sm4x). Please follow the journal's guidelines in preparing your
Please note that a copy of this checklist will be published alongside your article.

Abridged guidelines for figures
1. Data
The data shown in figures should satisfy the following conditions:

- the data were obtained and processed according to the field's best practice and are presented to reflect the results of the experiments in an accurate and unbiased manner.
- ideally, figure panels should include only measurements that are directly comparable to each other and obtained with the same assay.
- plots include clearly labeled error bars for independent experiments and sample sizes. Unless justified, error bars should not be shown for technical
- if n<5, the individual data points from each experiment should be plotted. Any statistical test employed should be justified.
- Source Data should be included to report the data underlying figures according to the guidelines set out in the authorship guidelines on Data

2. Captions
Each figure caption should contain the following information, for each panel where they are relevant:

- a specification of the experimental system investigated (eg cell line, species name).
- the assay(s) and method(s) used to carry out the reported observations and measurements.
- an explicit mention of the biological and chemical entity(ies) that are being measured.
- an explicit mention of the biological and chemical entity(ies) that are altered/varied/perturbed in a controlled manner.
- the exact sample size (n) for each experimental group/condition, given as a number, not a range;
- a description of the sample collection allowing the reader to understand whether the samples represent technical or biological replicates (including how many animals, litters, cultures, etc.).
- a statement of how many times the experiment shown was independently replicated in the laboratory.
- definitions of statistical methods and measures:
  - common tests, such as t-test (please specify whether paired vs. unpaired), simple χ2 tests, Wilcoxon and Mann-Whitney tests, can be unambiguously identified by name only, but more complex techniques should be described in the methods section;
  - are tests one-sided or two-sided?
  - are there adjustments for multiple comparisons?
  - exact statistical test results, e.g., P values = x but not P values < x;
  - definition of 'center values' as median or average;
  - definition of error bars as s.d. or s.e.m.

Please complete ALL of the questions below.
Select "Not Applicable" only when the requested information is not relevant for your study.

Materials

Newly Created Materials	Information included in the manuscript?	In which section is the information available? (Reagents and Tools Table, Materials and Methods, Figures, Data Availability Section)
New materials and reagents need to be available; do any restrictions apply?	Not Applicable	

Antibodies	Information included in the manuscript?	In which section is the information available? (Reagents and Tools Table, Materials and Methods, Figures, Data Availability Section)
For **antibodies** provide the following information:<br>- Commercial antibodies: RRID (if possible) or supplier name, catalogue number and or/clone number<br>- Non-commercial: RRID or citation	Yes	Materials and Methods

DNA and RNA sequences	Information included in the manuscript?	In which section is the information available? (Reagents and Tools Table, Materials and Methods, Figures, Data Availability Section)
**Short novel DNA or RNA including primers, probes**: provide the sequences.	Yes	Materials and Methods

Cell materials	Information included in the manuscript?	In which section is the information available? (Reagents and Tools Table, Materials and Methods, Figures, Data Availability Section)
**Cell lines:** Provide species information, strain. Provide accession number in repository **OR** supplier name, catalog number, clone number, and/**OR** RRID.	Yes	Materials and Methods
**Primary cultures:** Provide species, strain, sex of origin, genetic modification status.	Yes	Materials and Methods
Report if the cell lines were recently **authenticated** (e.g., by STR profiling) and tested for mycoplasma contamination.	Not Applicable	

Experimental animals	Information included in the manuscript?	In which section is the information available? (Reagents and Tools Table, Materials and Methods, Figures, Data Availability Section)
**Laboratory animals or Model organisms:** Provide species, strain, sex, age, genetic modification status. Provide accession number in repository **OR** supplier name, catalog number, clone number, **OR** RRID.	Not Applicable	
**Animal observed in or captured from the field:** Provide species, sex, and age where possible**.**	Not Applicable	
Please detail **housing and husbandry conditions**.	Not Applicable	

Plants and microbes	Information included in the manuscript?	In which section is the information available? (Reagents and Tools Table, Materials and Methods, Figures, Data Availability Section)
**Plants:** provide species and strain, ecotype and cultivar where relevant, unique accession number if available, and source (including location for collected wild specimens).	Not Applicable	
**Microbes:** provide species and strain, unique accession number if available, and source.	Not Applicable	

Human research participants	Information included in the manuscript?	In which section is the information available? (Reagents and Tools Table, Materials and Methods, Figures, Data Availability Section)
If collected and within the bounds of privacy constraints report on age, sex and gender or ethnicity for all study participants.	Not Applicable	

Core facilities	Information included in the manuscript?	In which section is the information available? (Reagents and Tools Table, Materials and Methods, Figures, Data Availability Section)
If your work benefited from core facilities, was their service mentioned in the acknowledgments section?	Yes	Acknowledgements

Design

Study protocol	Information included in the manuscript?	In which section is the information available? (Reagents and Tools Table, Materials and Methods, Figures, Data Availability Section)
If study protocol has been **pre-registered, provide DOI in the manuscript**. For clinical trials, provide the trial registration number **OR** cite DOI.	Not Applicable	
Report the **clinical trial registration number** (at ClinicalTrials.gov or equivalent), where applicable.	Not Applicable	

Laboratory protocol	Information included in the manuscript?	In which section is the information available? (Reagents and Tools Table, Materials and Methods, Figures, Data Availability Section)
Provide DOI OR other citation details if **external detailed step-by-step protocols** are available.	Yes	Materials and Methods

Experimental study design and statistics	Information included in the manuscript?	In which section is the information available? (Reagents and Tools Table, Materials and Methods, Figures, Data Availability Section)
Include a statement about **sample size** estimate even if no statistical methods were used.	Yes	Figure legends
Were any steps taken to minimize the effects of subjective bias when allocating animals/samples to treatment (e.g. **randomization procedure**)? If yes, have they been described?	Not Applicable	Materials and Methods
Include a statement about **blinding** even if no blinding was done.	Yes	Materials and Methods
Describe **inclusion/exclusion criteria** if samples or animals were excluded from the analysis. Were the criteria pre-established?<br><br>If sample or data points were omitted from analysis, report if this was due to attrition or intentional exclusion and provide justification.	Not Applicable	
For every figure, are **statistical tests** justified as appropriate? Do the data meet the assumptions of the tests (e.g., normal distribution)? Describe any methods used to assess it. Is there an estimate of variation within each group of data? Is the variance similar between the groups that are being statistically compared?	Yes	Figure legends

Sample definition and in-laboratory replication	Information included in the manuscript?	In which section is the information available? (Reagents and Tools Table, Materials and Methods, Figures, Data Availability Section)
In the figure legends: state number of times the experiment was **replicated** in laboratory.	Yes	Figure legends
In the figure legends: define whether data describe **technical or biological replicates**.	Yes	Figure legends

Ethics

Ethics	Information included in the manuscript?	In which section is the information available? (Reagents and Tools Table, Materials and Methods, Figures, Data Availability Section)
Studies involving **human participants**: State details of **authority granting ethics approval** (IRB or equivalent committee(s), provide reference number for approval.	Not Applicable	
Studies involving **human participants**: Include a statement confirming that **informed consent** was obtained from all subjects and that the experiments conformed to the principles set out in the WMA Declaration of Helsinki and the Department of Health and Human Services Belmont Report.	Not Applicable	
Studies involving **human participants:** For publication of **patient photos**, include a statement confirming that consent to publish was obtained.	Not Applicable	
Studies involving experimental **animals**: State details of **authority granting ethics approva**l (IRB or equivalent committee(s), provide reference number for approval. Include a statement of compliance with ethical regulations.	Not Applicable	
Studies involving **specimen and field samples:** State if relevant **permits** obtained, provide details of authority approving study; if none were required, explain why.	Not Applicable	

Dual Use Research of Concern (DURC)	Information included in the manuscript?	In which section is the information available? (Reagents and Tools Table, Materials and Methods, Figures, Data Availability Section)
Could your study fall under dual use research restrictions? Please check biosecurity documents and list of **select agents and toxins** (CDC): https://www.selectagents.gov/sat/list.htm	Not Applicable	
If you used a select agent, is the security level of the lab appropriate and reported in the manuscript?	Not Applicable	
If a study is subject to dual use research of concern regulations, is the name of the **authority granting approval and reference number** for the regulatory approval provided in the manuscript?	Not Applicable	

Reporting

The MDAR framework recommends adoption of discipline-specific guidelines, established and endorsed through community initiatives. Journals have their own policy about requiring specific guidelines and recommendations to complement MDAR.

Adherence to community standards	Information included in the manuscript?	In which section is the information available? (Reagents and Tools Table, Materials and Methods, Figures, Data Availability Section)
State if relevant guidelines or checklists (e.g., **ICMJE, MIBBI, ARRIVE, PRISMA**) have been followed or provided.	Not Applicable	
For **tumor marker prognostic studies,** we recommend that you follow the **REMARK** reporting guidelines (see link list at top right). See author guidelines, under 'Reporting Guidelines'. Please confirm you have followed these guidelines.	Not Applicable	
For **phase II and III randomized controlled trials**, please refer to the **CONSORT** flow diagram (see link list at top right) and submit the CONSORT checklist (see link list at top right) with your submission. See author guidelines, under 'Reporting Guidelines'. Please confirm you have submitted this list.	Not Applicable	

Data Availability

Data availability	Information included in the manuscript?	In which section is the information available? (Reagents and Tools Table, Materials and Methods, Figures, Data Availability Section)
Have **primary datasets** been deposited according to the journal's guidelines (see 'Data Deposition' section) and the respective accession numbers provided in the Data Availability Section?	Yes	Data availability section
Were **human clinical and genomic datasets** deposited in a public access-controlled repository in accordance to ethical obligations to the patients and to the applicable consent agreement?	Not Applicable	
Are **computational models** that are central and integral to a study available without restrictions in a machine-readable form? Were the relevant accession numbers or links provided?	Not Applicable	
If publicly available data were reused, provide the respective **data citations in the reference list**.	Not Applicable	
